# Development of risk reduction behavioral counseling for Ebola virus disease survivors enrolled in the Sierra Leone Ebola Virus Persistence Study, 2015-2016

**DOI:** 10.1371/journal.pntd.0005827

**Published:** 2017-09-11

**Authors:** Neetu Abad, Tasneem Malik, Archchun Ariyarajah, Patricia Ongpin, Matthew Hogben, Suzanna L. R. McDonald, Jaclyn Marrinan, Thomas Massaquoi, Anna Thorson, Elizabeth Ervin, Kyle Bernstein, Christine Ross, William J. Liu, Karen Kroeger, Kara N. Durski, Nathalie Broutet, Barbara Knust, Gibrilla F. Deen, on behalf of the Sierra Leone Ebola Virus Persistence Study Group

**Affiliations:** 1 Social & Behavioral Research and Evaluation Branch, Division of Sexually Transmitted Disease Prevention, National Center for HIV/AIDS, Viral Hepatitis, STD, & TB Prevention, Centers for Disease Control and Prevention, Atlanta, Georgia, United States of America; 2 Office of Global Activities, Division of Sexually Transmitted Disease Prevention, National Center for HIV/AIDS, Viral Hepatitis, STD, & TB Prevention, Centers for Disease Control and Prevention, Atlanta, Georgia, United States of America; 3 Comprehensive Care & Support for EVD Survivors, EVD Research, World Health Organization, Freetown, Sierra Leone; 4 Strategic information, Joint United Nations Programme on HIV/AIDS, Freetown, Sierra Leone; 5 Military Hospital 34, Sierra Leone Ministry of Defence, Freetown, Sierra Leone; 6 Department of Reproductive Health and Research, Family, Women's and Children's Health, World Health Organization, Geneva, Switzerland; 7 Viral Special Pathogens Branch, Division of High Consequence Pathogens and Pathology, National Center for Emerging and Zoonotic Infectious Diseases, Centers for Disease Control and Prevention, Atlanta, Georgia, United States of America; 8 Epidemiology and Statistics Branch, Division of Sexually Transmitted Disease Prevention, National Center for HIV/AIDS, Viral Hepatitis, STD, & TB Prevention, Centers for Disease Control and Prevention, Atlanta, Georgia, United States of America; 9 HIV Care and Treatment Branch, Division of Global HIV/AIDS and Tuberculosis, Center for Global Health, Centers for Disease Control and Prevention, Atlanta, Georgia, United States of America; 10 National Institute for Viral Disease Control and Prevention, Chinese Center for Disease Control and Prevention, Beijing, People's Republic of China; 11 Sierra Leone-China Friendship Biological Safety Laboratory, Chinese Center for Disease Control and Prevention, Freetown, Sierra Leone; 12 Pandemic and Epidemic Disease Department, Outbreaks and Health Emergencies, World Health Organization, Geneva, Switzerland; 13 Connaught Hospital; Sierra Leone Ministry of Health and Sanitation, Freetown, Sierra Leone; The University of Kansas, UNITED STATES

## Abstract

**Background:**

During the 2014–2016 West Africa Ebola Virus Disease (EVD) epidemic, the public health community had concerns that sexual transmission of the Ebola virus (EBOV) from EVD survivors was a risk, due to EBOV persistence in body fluids of EVD survivors, particularly semen. The Sierra Leone Ebola Virus Persistence Study was initiated to investigate this risk by assessing EBOV persistence in numerous body fluids of EVD survivors and providing risk reduction counseling based on test results for semen, vaginal fluid, menstrual blood, urine, rectal fluid, sweat, tears, saliva, and breast milk. This publication describes implementation of the counseling protocol and the key lessons learned.

**Methodology/Principal findings:**

The Ebola Virus Persistence Risk Reduction Behavioral Counseling Protocol was developed from a framework used to prevent transmission of HIV and other sexually transmitted infections. The framework helped to identify barriers to risk reduction and facilitated the development of a personalized risk-reduction plan, particularly around condom use and abstinence. Pre-test and post-test counseling sessions included risk reduction guidance, and post-test counseling was based on the participants’ individual test results. The behavioral counseling protocol enabled study staff to translate the study’s body fluid test results into individualized information for study participants.

**Conclusions/Significance:**

The Ebola Virus Persistence Risk Reduction Behavioral Counseling Protocol provided guidance to mitigate the risk of EBOV transmission from EVD survivors. It has since been shared with and adapted by other EVD survivor body fluid testing programs and studies in Ebola-affected countries.

## Author summary

The 2014–2016 West Africa Ebola Virus Disease (EVD) epidemic was large and widespread, affecting thousands of people across Guinea, Liberia, and Sierra Leone. Prior to this epidemic, there were limited data on persistence of Ebola virus in body fluids of EVD survivors and the potential risk that viral persistence may pose for Ebola virus transmission, including possible sexual transmission. This paper documents the development and implementation of a behavioral counseling protocol to facilitate adoption of risk reduction behaviors among male and female EVD survivors enrolled in the Sierra Leone Ebola Virus Persistence Study. This behavioral counseling protocol, composed of pre-test, delivery of results, and post-test counseling, enabled study staff to translate the study’s body fluid test results into individualized information and preventive action for study participants. Risk reduction behavioral counseling became an important EVD epidemic control measure.

## Introduction

The 2014–2016 West Africa Ebola Virus Disease (EVD) epidemic was large and widespread, with at least 28,616 probable and suspected cases of EVD and 11,310 deaths across Guinea, Liberia, and Sierra Leone [1]. Limited studies during outbreaks prior to 2014 showed evidence of persistent Ebola virus (EBOV) in semen [2]. In the 1995 Kikwit outbreak in the Democratic Republic of Congo, viable virus in semen was detected 82 days post symptom onset and EBOV ribonucleic acid (RNA) was detected 101 days post symptom onset [3]. Less evidence was found for short-term EBOV persistence in vaginal and rectal fluids as well as urine and sweat [2]. In West Africa, EVD survivors who recovered from disease were initially advised by the World Health Organization (WHO) to abstain from sex or to use condoms for at least three months after discharge from an Ebola Treatment Unit (ETU) [4]. As the number of individuals who survived EVD in West Africa grew into the thousands, questions were raised regarding EBOV persistence in semen and in other body fluids of EVD survivors, and potential implications of viral persistence on residual risk of EBOV transmission from EVD survivors beyond the original three month recommendations [2, 5, 6].

Given limited pre-existing data, the potential for EBOV to persist in survivors was identified as an important research topic early in the epidemic. There was also a programmatic need to develop methods to test body fluids of EVD survivors and counsel them on risk reduction practices. This need became even more clear when a woman from Liberia tested positive for EBOV infection, and epidemiologic investigation found that her only exposure was unprotected vaginal intercourse with a male EVD survivor whose semen tested positive for EBOV RNA by real time reverse transcriptase polymerase chain reaction (qRT-PCR) 199 days after he first became symptomatic with EVD [7, 8]. Sequencing of the RNA from the semen of the male EVD survivor closely matched the sequence recovered from the female patient’s blood, providing further evidence for male-to-female sexual transmission of EBOV long into convalescence [8].

### Sexual behavior in Sierra Leone

Prior to the EVD epidemic, the Demographic Health Survey conducted in 2013 in Sierra Leone showed that condom use was low, with 5% of female and 13% of male respondents reporting having used a condom in the past 12 months. Twenty-five percent of men and 6% of women reported having two or more sex partners in the last 12 months, and 23% of men and 5% of women reported concurrent sexual partnerships. Multiple and concurrent sexual partnerships were highest among older married men with low education [9]. Human Immunodeficiency Virus (HIV) prevalence was 1.5% among adults aged 15–49 years [9]. There is limited information regarding the impact of the EVD epidemic on sexual risk behavior, but adolescent girls in Sierra Leone reported more unplanned pregnancies and engagement in transactional sex in the nine months during the epidemic when public schools were closed [10]. In addition, EVD survivors reported stigma and feelings of bereavement similar to those experienced by people living with HIV, possibly negatively affecting both their quality of life and intimate relationships, and their motivation to seek healthcare and other services [11–13]. The impact of the EVD epidemic on the sexual behavior of EVD survivors has not yet been fully explored.

### Sierra Leone Ebola Virus Persistence Study

In May 2015, the Sierra Leone Ebola Virus Persistence Study was launched to investigate EBOV persistence in the body fluids of EVD survivors in Sierra Leone [5]. The study consisted of two phases. The first phase assessed EBOV persistence in semen of 100 adult male EVD survivors, and the second phase assessed EBOV persistence in semen and additional body fluids (vaginal fluid, menstrual blood, urine, rectal fluid, sweat, saliva, tears, and breast milk as applicable by sex) in 120 male and 120 female EVD survivors. Male and female EVD survivors living with HIV were also invited to participate in the study to characterize EBOV persistence among EVD survivors living with HIV. The study took place in two sites: Military Hospital 34 (an urban facility in Freetown, Western District) and Lungi Government Hospital (a semi-rural facility in Lungi, Port Loko District). In this paper, we discuss the development and implementation of the Ebola Virus Persistence Risk Reduction Behavioral Counseling Protocol (henceforth referred to as the behavioral counseling protocol) used in the study. The test results from this study will be published separately and the overall study design has been described elsewhere [5].

## Materials and methods

The objectives of the behavioral counseling protocol within the study were to: (1) provide participants simple explanations of qRT-PCR and virus isolation testing and deliver individual test results; and (2) encourage participants to engage in risk reduction behavioral practices corresponding to their individual qRT-PCR test results until the participant received two consecutive negative qRT-PCR test results. Counselors also referred participants to available EVD survivor services in the community when necessary.

### Ethics statement

Although the authors note the importance of acquiring information on the persistence of EBOV in various body fluids within the pediatric population, for ethical reasons, this study was limited to adults aged 18 years or older. All participants provided written informed consent at the first study visit. The study was approved by the Sierra Leone Ethics and Scientific Review Committee and the WHO Ethical Review Committee (No. RPC736).

### Development of the behavioral counseling protocol

We sought to reduce sexual exposure to EBOV by behavior change strategies such as abstinence, increased condom use, and choice of partners. To do this, we developed a behavioral counseling protocol adapted from the Project RESPECT *Brief Counseling* intervention, an individual face-to-face counseling model that has been proven effective at reducing both new sexually transmitted infections (STIs) and risky behaviors in randomized controlled trials [14]. RESPECT’s *Brief Counseling* was implemented in the context of Human Immunodeficiency Virus (HIV) testing, and included two 20-minute counseling sessions (i.e. pretest/posttest) in which the counselor supported risk reduction behaviors by increasing the client’s perception of personal risks, emphasizing self-efficacy and personalized goal setting through identifying concrete, incremental and achievable risk-reduction steps that limited sexual HIV/STI exposure. STI clinic patients were asked to describe their own sexual risk behaviors, and misconceptions about risk were clarified. Counselors supported clients in identifying personal barriers to risk reduction and possible ways to overcome them, and helped clients to identify and negotiate behavioral risk reduction steps relevant to their personal risk behaviors. RESPECT’s Brief Intervention model has been previously adapted in behavior change interventions aimed at reducing STIs and pregnancy in vulnerable populations residing in high HIV prevalence settings [15, 16, 17, 18]. The intervention appears to be particularly effective among those with limited exposure to HIV/STI prevention guidance [15, 16, 17, 18]. In sum, the rationale for using a RESPECT model for reducing sexual EBOV exposure was that the approach (1) has demonstrated efficacy at reducing behaviors relevant to sexual EBOV transmission (e.g., increasing abstinence, increasing condom use, reducing risky sexual partnerships), (2) uses a flexible approach that meets the client at his or her own understanding, (3) has been shown to be effective using counselors that are trained in adherence to the model but do not have advanced degrees in counseling, and (4) has been successfully adapted in many international settings with varying cultural contexts.

The adaptation of Project RESPECT for male and female EVD survivors enrolled in the Sierra Leone Ebola Virus Persistence Study required several changes to HIV/STI prevention behavioral guidance. These changes were made in consultation with HIV service providers operating in Sierra Leone [e.g. Sierra Leone National AIDS Control Programme, National AIDS Secretariat, Dignity Association, Joint United Nations Programme on HIV/AIDS (UNAIDS)].

### Protocol visit flow

**Fig 1:**
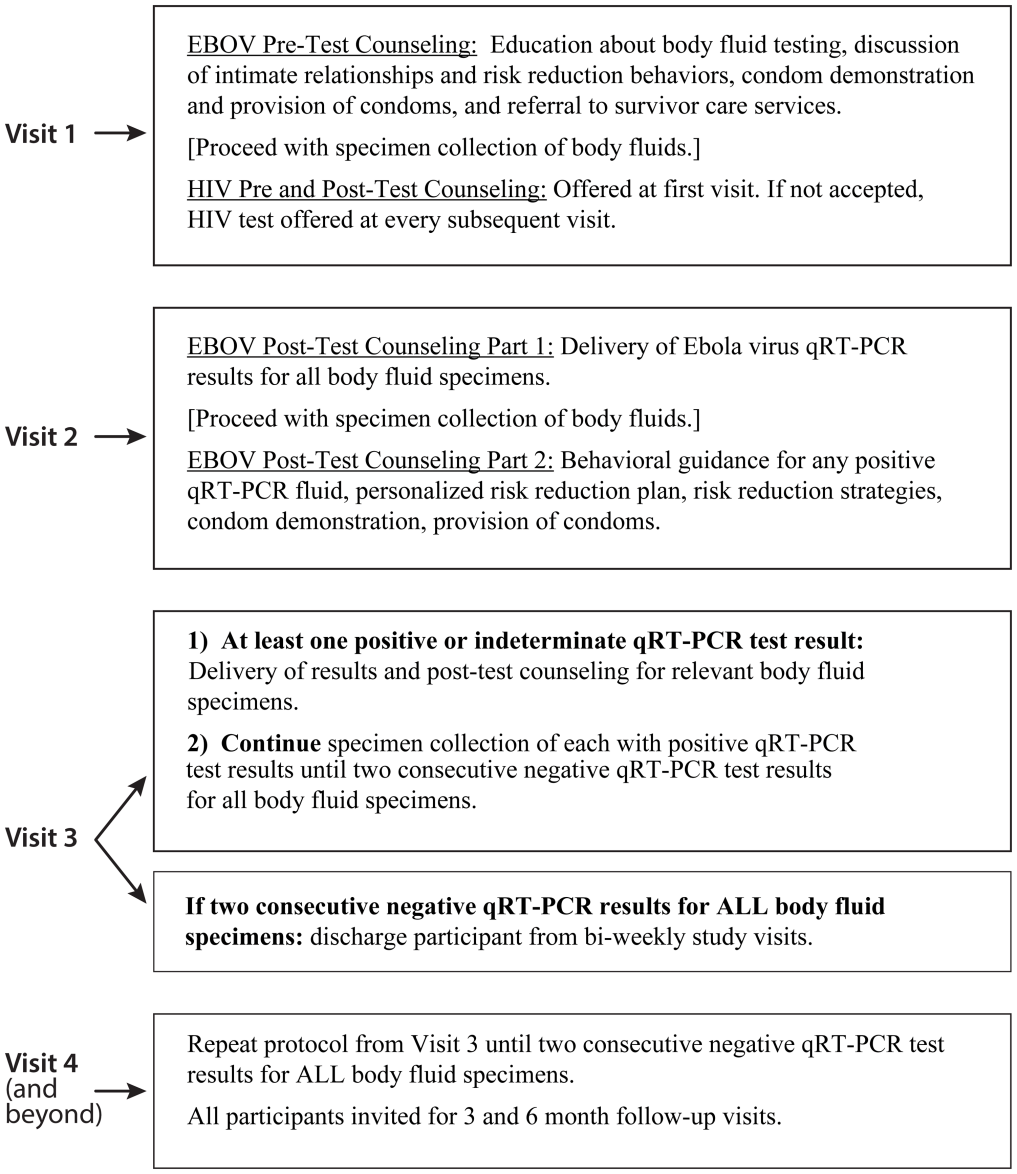
Visit flow chart. The Ebola Virus Persistence Risk Reduction Behavioral Counseling Protocol visit flow chart. **(A) Visit 1** Description of EBOV pre-test counseling, and HIV pre and post-test counseling procedures. **(B)Visit 2** Description of EBOV post-test counseling part 1 and 2 procedures. **(C) Visit 3** Description of procedures if at least one positive, indeterminate, or no interpretation qRT-PCR test results was found, or if all body fluid specimens were found qRT-PCR negative. **(D) Visit 4 and Beyond:** Description of repeat visits until two consecutive negative qRT-PCR test results for all body fluid specimens.

The standard operating procedure for the behavioral counseling protocol included EBOV persistence pre-and post-test counseling as well as HIV pre-and post-test counseling. EBOV persistence pre-test counseling is an introduction to EBOV testing and risk reduction advice prior to testing, while post-test counseling is a delivery of tailored guidance based on qRT-PCR test results. Fig 1 shows the typical participant visit flow.

#### First visit

During the first visit, participants were first seen by a nurse who described the study, obtained informed consent, and facilitated the demographic, behavioral, and clinical questionnaire. After specimen collection, each participant visited a counselor for pre-test counseling. In this session, counselors educated participants about qRT-PCR and virus isolation testing methods; asked participants about their sexual behavior, intimate relationships, and life stressors including experiences with stigma; gave a male and female condom demonstration; and referred to survivor care services if needed. Rapid HIV testing and counseling was offered to each participant at their first visit after specimen collection, and, if they did not initially consent, at every visit thereafter.

#### Second visit

During visit 2, held two weeks after initial specimen collection at the first visit, counselors and participants met for post-test counseling. In the first part of post-test counseling, counselors delivered the prior visit’s qRT-PCR test results for each specimen: positive, negative, indeterminate, or no interpretation (poor specimen quality) [5]. Instead of describing the results as positive, negative, indeterminate, or no interpretation, counselors explained that pieces of Ebola virus genetic material were detected (seen), not detected (not seen), or could not be determined or cannot be interpreted (cannot tell). These terms were used to differentiate EBOV persistence test results from the ‘positive’ or ‘negative’ test results delivered to patients awaiting confirmation of an EVD diagnosis in ETUs. This change was suggested by study staff and representatives from the EVD survivor community, who expressed concern that a participant might interpret a “positive” test result to imply that he or she was ill with acute EVD again. Positive qRT-PCR test results were explained as “pieces of Ebola virus were seen” in order to describe that specific sequences of virus RNA were detected but did not necessarily mean that infectious virus was present in the given specimen.

Following the delivery of test results for specimens collected during the previous visit, participants provided a second set of specimens for qRT-PCR testing and then visited the counselor for further post-test counseling. This order was used to prevent delays in study flow. During the second part of post-test counseling, counselors reviewed test results, delivered behavioral guidance for any positive qRT-PCR fluid, developed a personalized risk reduction plan and negotiated risk reduction strategies, gave a condom demonstration, and distributed condoms.

#### Third (and as applicable, any other follow-up) visit(s)

Participants continued to provide specimens and receive post-test counseling for all test results. Study visits continued until participants received two consecutive negative qRT-PCR test results on all body fluids, at which time participants were discharged from bi-weekly study visits, and asked to come back for a follow-up visit with specimen collection after three and six months. The counseling provided at these latter follow-up visits followed the same messaging as described above. Positive virus isolation results were delivered when they became available, which occurred several months after the initial specimen was collected, as these specimens were sent to the United States for testing. If virus isolation results were positive, participants were told that “the virus found in your specimen when it was tested was alive and possibly infectious”.

At all sessions, counselors asked participants to report any medical, psychosocial, or other issues they may have been experiencing for referral to community-based services where available. Additionally, if a participant could not provide a specimen at any visit, the counselor discussed specimen collection strategies and scheduled another visit for the participant [19].

### Behavioral guidance for all body fluids

Table 1 shows the behavioral risk reduction guidance corresponding to each body fluid tested. In the event of a positive qRT-PCR test result, relevant guidance was delivered to the participant in order to reduce transmission risk. All standard operating procedures associated with the delivery of guidance at pre-test and post-test counseling can be found in the full Ebola virus persistence study behavioral counseling protocol here: S1 Protocol.

**Table 1:** Behavioral guidance for positive qRT-PCR test results on body fluid specimens.

	Semen	Vaginal fluid	Menstrual blood	Urine	Rectal fluid	Sweat	Tears	Saliva	Breast milk
**Timing of guidance delivery**									
• EBOV persistence pre-test counseling	✓								
• EBOV persistence post-test counselling	✓	✓	✓	✓	✓	✓	✓	✓	✓
**Sexual risk reduction guidance**									
• Abstain from manual, oral, vaginal or anal sex. If you have sex, use a male or female condom.	✓	✓	✓	✓	✓				
• Abstain only						✓			
• Abstain from kissing and oral sex only								✓	
• Avoid kissing and touching of breasts during sex								✓	✓
**Sexual risk reduction guidance if sexual activity occurs**									
• After sex, masturbation, or any genital contact, wash and dry or thoroughly and wipe genital and anal area with a clean towel or tissue but do not use the same towel or tissue on both areas	✓	✓	✓	✓	✓				
• Safely throw away tissues with body fluids, and/or used condom somewhere where other people cannot touch it, like a covered bin, pit latrine, or burn pit. For towels, wash in hot water before reusing	✓	✓	✓	✓	✓				
• After sex or any genital contact, wash hands thoroughly with soap and water	✓	✓	✓	✓	✓				
• No sexual risk reduction guidance							✓		
**General infection prevention and control (IPC) guidance**									
• Wash hands often with soap and water	✓	✓	✓	✓	✓	✓	✓	✓	✓
• Try not to touch your eyes, nose, and mouth with unwashed hands.	✓	✓	✓	✓	✓	✓	✓	✓	✓
• After urinating or defecating, wash hands well with soap and water.	✓	✓	✓	✓	✓				
• Cover your mouth and nose when sneezing or coughing.								✓	
• Do not share personal items such as razors, toothbrushes or eating utensils with others						✓		✓	
• Use clean tissues or towels to wipe body, hands, mouth, eyes						✓	✓	✓	✓
**Breastfeeding guidance**									
• Do not breastfeed until pieces of the Ebola virus are not detected in two consecutive specimens									✓

^✓^ Indicates guidance delivered for positive qRT-PCR test results for that particular body fluid. Participants were counseled to continue following the guidance until they tested qRT-PCR negative twice consecutively.

As prior evidence showed that EBOV could persist in semen [2], male EVD survivors were encouraged at pre-test counseling to use condoms or engage in abstinence for the prevention of EBOV transmission as well as prevention of HIV/STI transmission and unwanted pregnancy. Given limited or no prior evidence for EBOV persistence in vaginal fluids, menstrual blood, urine, rectal fluids, sweat, saliva, tears, and breast milk, precautionary guidance was not provided at pre-test counseling for any of these body fluids. Women were advised at pre-test counseling to use condoms or abstain from sexual intercourse to prevent HIV/STI acquisition and unwanted pregnancy [2]. Female participants were given sexual risk reduction guidance to prevent EBOV transmission only in the event of a positive qRT-PCR test result.

Sexual risk reduction behavioral guidance was tailored for each type of body fluid. Participants were given different guidance for fluids that posed a greater risk for sexual transmission of EBOV due to contact during sexual activity (semen, vaginal fluids, menstrual blood, rectal fluid, urine, sweat, saliva) as compared to fluids for which there was likely to be less contact during sexual activity (breast milk, tears) [2]. Sexual risk reduction guidance was tailored to participants’ religious and cultural practices, particularly around genital washing. For example, counselors reported that Muslim participants preferred to wash the genital area or entire body after sexual intercourse, while Christian participants preferred to wipe genital areas with tissues after sexual intercourse.

For non-sexual risk reduction behavioral guidance, the study team reviewed general infection prevention and control (IPC) guidelines for other pathogens that are spread through contact with infectious body fluids such as viral meningitis and hepatitis B (http://www.cdc.gov/meningitis/viral.html). This guidance was modified for a low resource setting, taking into consideration limited access to clean water and sustained power sources, and lack of flushing toilets, bleach, or other materials and practices used in standard IPC protocols.

Special consideration was given to shaping guidance for female EVD survivors. A focus group with female EVD survivors was held prior to the enrollment of women in the second phase of the study. Participants discussed experience with stigma due to their status as an EVD survivor, fear of resuming sexual activity, and concerns that their partner would not accept condom use. Information from this focus group was shared with study counselors as preparation for discussions with participants regarding specimen collection, sexual activity, and condom use among female participants. Given high rates of sexual violence among women in Sierra Leone [9], a referral pathway for intimate partner violence (IPV) was established in accordance with IPV service provision guidelines from the government of Sierra Leone. Additionally, if a breast milk specimen of a breastfeeding female survivor were to test positive for EBOV, she would be immediately asked to switch to replacement feeding using ready-to-use infant formula provided free of charge with support from UNICEF, and receive ongoing counseling and support from Sierra Leone MoHS Food and Nutrition Directorate staff or a trained nurse.

### Counselor recruitment & training

Given the complex nature of the behavioral guidance for all body fluids, particularly for semen and other intimate fluids, counselors with a background in HIV/STI and/or mental health nursing with previous experience with the EVD epidemic were recruited for the study. At Military 34 Hospital, one female HIV/STI nurse and one male mental health nurse were chosen. At Lungi Government Hospital, one woman with a background in HIV/STI nursing and one woman with a background in midwifery were chosen.

For the first phase of the study, we developed a four-day training package that focused on the science of EBOV transmission and diagnostic testing methods, how to effectively discuss sexual behavior and risk reduction, and common mental health/psychosocial issues reported by EVD survivors. We held an additional four day-training focused on HIV testing and counseling. For the second phase of the study, we added a four-day training to address behavioral guidance for additional body fluids, and to assess for IPV among female study participants in accordance with national and international guidelines [20]. These trainings were primarily geared towards study counselors but all study staff were invited to attend trainings relevant to their roles.

The counselors used role-playing to practice using counseling scripts that had been translated into Krio language and then back-translated for the first phase of the study. The Sierra Leone National AIDS Control Programme, Dignity Association, and UNAIDS provided training on HIV testing and counseling, condom demonstrations, and stigma and discrimination. During and after the training sessions, suggestions from the counselors were incorporated in the counseling scripts and behavioral guidance.

## Discussion

The development and implementation of a novel Ebola Virus Persistence Risk Reduction Behavioral Counseling Protocol as part of the Sierra Leone Ebola Virus Persistence Study was an important part of translating semen testing science into individualized, preventive guidance for study participants. It presented a unique opportunity to provide health education and test-based risk reduction services to EVD survivors at a time when these services were not available in a national programmatic setting. Baseline data from the first phase of the study demonstrated that 49% of phase 1 participants had positive baseline qRT-PCR results, and that EBOV RNA could persist in the semen of male EVD survivor participants for at least 270 days (9 months) post-symptom onset [5]. Results from subsequent phases of the study will be published separately. Although we had limited pre-existing information regarding the infectiousness and transmission potential of qRT-PCR positive body fluids, the behavioral counseling protocol became a very important tool to translate laboratory test results into direct guidance for the study participants to potentially prevent EBOV transmission.

EVD survivors in West Africa have reported experiencing a myriad of difficulties including stigma from family, friends, and community members, loss of employment, housing, and social networks, and numerous mental or physical health sequelae [11, 13, 21, 22, 23, 24]. The behavioral counseling protocol was developed to provide EVD survivor participants a safe place to discuss often complex concerns regarding EBOV persistence, semen testing, and other issues related to EVD survivorship. It is possible that positive experiences with the behavioral counseling protocol contributed to participants’ willingness to maintain continued participation in semen testing activities, in addition to transportation compensation, access to referral medical care, and receipt of critical health information.

### Health and risk communication

We observed great receptivity to condom demonstrations, with many participants informing research staff that they had not participated in a condom demonstration prior to joining the study. Given the low national rates of condom use among men and women in Sierra Leone [9], exposure to condoms via the behavioral counseling protocol may eventually be associated with gains in reproductive health and STI prevention in participants.

Communicating the risk of sexual transmission of EBOV to study participants was challenging as there was limited scientific evidence regarding the length of persistence and the infectiousness of positive qRT-PCR body fluids. In this study, we observed that communicating uncertainty in a transparent way appeared to facilitate trust between participants and the research study. For example, following the report of an EVD survivor in the United Kingdom who relapsed with EVD meningitis [22], the behavioral counseling protocol was adapted to ensure that participants were aware of possible severe relapses. Future development of similar counseling protocols may consider embracing scientific transparency as a trust-building communication tool.

In the course of the study, we identified a need to clearly and simply explain the detection method of the qRT-PCR assay to participants. We developed a “mango tree” analogy comparing detection of EBOV-specific RNA primers (short target sequences) in the body fluids of Ebola survivors via qRT-PCR testing to trying to detect a whole mango tree (the intact, viable Ebola virus) by being able to detect only the mango fruits or the leaves (the target RNA primers for EBOV). Detecting only fruits or leaves, one cannot determine whether the tree is indeed intact and alive just as qRT-PCR testing can only determine whether the target RNA primers are detected or undetected in a body fluid specimen, and not whether the virus is infectious. Detecting only one of the two target RNA sequences (an indeterminate test result), could also be explained using this analogy, and also to explain possible variation in test results using different qRT-PCR assays with differing target sequences. This addition to the counseling script was very helpful for study participants to conceptualize the test, the different results they encountered, and the associated counseling messages.

A flexible protocol was also instrumental in optimizing participant flow during the study. For example, some participants preferred to visit the counselor prior to specimen collection for more detailed instructions on specimen collection processes or for extra encouragement to continue with the testing process. Talking points were developed as reference materials for study counselors as the original scripts were lengthy. Because we believed that participants should receive their previous test results before deciding whether to donate specimens for testing at the current visit, there was a delay between delivery of test results and post-test counseling and explanation of guidance. Future iterations of similar counseling protocols may be able to concurrently deliver testing results and post-test counseling if a rapid diagnostic test for detection of Ebola RNA in the body fluids of Ebola survivors becomes available.

### Health systems strengthening

The EVD epidemic had a devastating impact on the economic and social ties of communities across Sierra Leone [12, 21, 24]. EVD survivors commonly reported medical and mental health issues post-convalescence for which there was limited assistance in Sierra Leone [25]. These needs were far greater than the services the study could offer. This was particularly true for semen testing, as there were far more EVD survivors in Sierra Leone than could be enrolled in the EBOV persistence study. To this end, the Government of Sierra Leone initiated a comprehensive program for EVD survivors (CPES) in October 2015 that aimed to provide services for EVD survivors in Sierra Leone including counseling, semen testing, eye care, myalgia, and treatment for other EVD sequelae [26].

The mental health infrastructure of Sierra Leone is underdeveloped. There is limited capacity to identify and treat mental health disorders in the general public or EBOV-affected populations [12]. When the study was initiated, counseling staff were less familiar with behavioral counseling methods that emphasized dialogue regarding participants’ experiences with EVD survivorship, stigma, and intimate relationships. Learning these skills helped study counselors better assist participants who received multiple positive qRT-PCR test results to maintain participation in semen testing and openly discuss feelings of frustration and anxiety.

### HIV testing & counseling uptake

After observing initial high rates of HIV testing uptake in the first phase of the study, acceptance of HIV testing declined among study participants during the first visit. We hypothesize that participants may have felt anxious about their EBOV qRT-PCR tests and did not want to learn their HIV test results at the same time. We re-trained study counselors on HIV testing and counseling procedures and encouraged them to offer HIV tests at follow-up study visits and observed a rise in uptake of HIV testing. More formative research is needed to determine how best to incorporate both HIV and EBOV testing and counseling in future EVD epidemics.

### Limitations

One limitation of our behavioral counseling protocol is that, due to time constraints during study visits, we did not employ couples’ counseling. Future development of similar EBOV persistence behavioral counseling protocols may consider the inclusion of couples’ counseling to facilitate correct and consistent condom use, particularly for those participants who repeatedly test positive [27]. Consideration could also be given on how best to recruit, enroll, and counsel EVD survivors who are men who have sex with men (MSM), and who may be reluctant to discuss their sexual behavior in an environment where same sex behavior is criminalized [9], as well as survivors who may object to body fluid testing due to religious objections to masturbation or other specimen donation procedure.

Another limitation of the protocol is that we developed and implemented risk reduction guidance when relatively little was known about the persistence of Ebola RNA in the body fluids of survivors, or how qRT-PCR results related to the risk of transmitting Ebola to others. Since the implementation of this behavioral counseling protocol, additional data on the persistence of Ebola RNA in semen has become available from multiple Ebola-affected countries [28–31]. These data show Ebola RNA persisting in semen for lengthy periods of time for some Ebola survivors, and further illustrate the critical role that semen testing and behavioral counseling can play in Ebola epidemic control [32]. Future development of similar behavioral counseling protocols should consider these additional data in adapting behavioral guidance for Ebola survivors, such as collecting body fluid specimens more frequently and increasing the number of consecutive negative tests needed before testing ceases following the receipt of positive test results.

### Conclusion

A behavioral counseling protocol that pairs test results with risk reduction behavioral guidance might help mitigate transmission risks associated with body fluids of EVD survivors in which EBOV has been detected. Risk reduction behavioral counseling is rapidly becoming an integral part of addressing the sexual transmission risk in this EVD epidemic and in filovirus outbreaks moving forward; its utility will likely also be swiftly demonstrated for other pathogens where virus persistence in body fluids may pose a risk for continued transmission from survivors [32]. A qualitative evaluation assessing the impact of the behavioral counseling protocol on study participants as well as staff perceptions of the protocol has been performed to add to lessons learned presented in this manuscript.

As of July 2016, the Ebola Virus Persistence Risk Reduction Behavioral Counseling Protocol has been used for more than 220 male and 120 female participants. The protocol has been adapted for use by other body fluid testing programs for EVD survivors, including the Government of Sierra Leone’s CPES (Alpren, C., personal communication) and Liberia’s Men’s Health Screening Program [29]. Lessons learned from implementation of the behavioral counseling protocol in the Sierra Leone Ebola Virus Persistence Study have also been shared to advise the operations of these semen testing programs [29]. We hope that our experience implementing this behavioral counseling protocol in the midst of an EVD epidemic in Sierra Leone can inform similar future efforts so that robust and effective services can be provided to EVD survivors.

## Supporting information

S1 Checklist STROBE checklist. (DOC)

S1 Protocol Ebola Virus Persistence Risk Reduction Behavioral Counseling Protocol. (DOCX)

## References

[1] World Health Organization. Situation Report Ebola 2015. 10 June 2016.

[2] Thorson A, Formenty P, Lofthouse C, Broutet N. Systematic review of the literature on viral persistence and sexual transmission from recovered Ebola survivors: evidence and recommendations. BMJ Open. 2016;6(1). 10.1136/bmjopen-2015-008859 PMC4716240 26743699

[3] Rodriguez LL, De Roo A, Guimard Y, Trappier SG, Sanchez A, Bressler D, et al Persistence and genetic stability of Ebola virus during the outbreak in Kikwit, Democratic Republic of the Congo, 1995. The Journal of infectious diseases. 1999;179 Suppl 1:S170–6. 9988181 10.1086/514291

[4] World Health Organization. Ebola Virus Disease Fact Sheet. 2016.

[5] Deen GF, Knust B, Broutet N, Sesay FR, Formenty P, Ross C, et al Ebola RNA Persistence in Semen of Ebola Virus Disease Survivors—Preliminary Report. New England Journal of Medicine. 2015 10. 10.1056/NEJMoa1511410 PMC5798881 26465681

[6] Fischer WA, Wohl DA. Confronting Ebola as a Sexually Transmitted Infection. Clinical Infectious Diseases. 2016. 10.1093/cid/ciw123 PMC4845792 26936667

[7] Christie A, Davies-Wayne GJ, Cordier-Lasalle T, Blackley DJ, Laney AS, Williams DE, et al Possible sexual transmission of Ebola virus—Liberia, 2015. MMWR Morbidity and mortality weekly report. 2015;64(17):479–81. 25950255 PMC4584553

[8] Mate SE, Kugelman JR, Nyenswah TG, Ladner JT, Wiley MR, Cordier-Lassalle T, et al Molecular Evidence of Sexual Transmission of Ebola Virus. New England Journal of Medicine. 2015;373(25):2448–54. doi: 10.1056/NEJMoa1509773 26465384 10.1056/NEJMoa1509773 PMC4711355

[9] International I. Sierra Leone: Demographic and Health Survey 2013. 2013.

[10] UNFPA. Rapid Assessment of Ebola impact on reproductive health services and service seeking behavior in Sierra Leone. 2015.

[11] Davtyan M, Brown B, Folayan MO. Addressing Ebola-related Stigma: Lessons Learned from HIV/AIDS. Global Health Action. 2014;7. 10.3402/gha.v7.26058 PMC4225220 25382685

[12] Shultz JM, Baingana F, Neria Y. The 2014 ebola outbreak and mental health: Current status and recommended response. JAMA. 2015;313(6):567–8. doi: 10.1001/jama.2014.17934 25532102 10.1001/jama.2014.17934

[13] Van Bortel T, Basnayake A, Wurie F, Jambai M, Koroma AS, Muana AT, et al Psychosocial effects of an Ebola outbreak at individual, community and international levels. Bulletin of the World Health Organization. 2016;94(3):210–4. doi: 10.2471/BLT.15.158543 26966332 10.2471/BLT.15.158543 PMC4773931

[14] Kamb ML, Fishbein M, Douglas JM Jr, et al Efficacy of risk-reduction counseling to prevent human immunodeficiency virus and sexually transmitted diseases: A randomized controlled trial. JAMA. 1998;280(13):1161–7. 9777816 10.1001/jama.280.13.1161

[15] Fishbein M. T T, Kanfer FH., Becker M., Middlestadt SE., Eichler A. Factors influencing behavior and behavior change. Mahwah New Jersey: Erlbaum; 2001.

[16] Fisher JD. F W, editor. Theoretical approaches to individual-level change in HIV risk behavior New York: Kluwer; 2000.

[17] Jemmott JB, Jemmott LS, O’Leary A, Ngwane Z, Icard LD, Heeren GA, et al Cluster-Randomized Controlled Trial of an HIV/Sexually Transmitted Infection Risk-Reduction Intervention for South African Men. American Journal of Public Health. 2014;(104):467–73. doi: 10.2105/AJPH.2013.301578 24432923 10.2105/AJPH.2013.301578 PMC3953794

[18] Wariki WM, Ota E, Mori R, Koyanagi A, Hori N, Shibuya K. Behavioral interventions to reduce the transmission of HIV infection among sex workers and their clients in low- and middle-income countries. The Cochrane database of systematic reviews. 2012. 10.1002/14651858.CD005272.pub3 PMC11345029 22336811

[19] Deen GF. Implementation of a Research Study to Examine Persistence of Ebola Virus Zaire in Body Fluids of Ebola Virus Disease Survivors in Sierra Leone: Methodology and Lessons Learned. PLOS Neglected Tropical Diseases. 2017. 10.1371/journal.pntd.0005723 PMC5593174 28892501

[20] Responding to intimate partner violence and sexual violence against women. World Health Organization 2013. 24354041

[21] Vetter P, Kaiser L, Schibler M, Ciglenecki I, Bausch DG. Sequelae of Ebola virus disease: the emergency within the emergency. The Lancet Infectious Diseases. 2016. 10.1016/S1473-3099(16)00077-3 27020309

[22] Jacobs M, Rodger A, Bell DJ, Bhagani S, Cropley I, Filipe A, et al Late Ebola virus relapse causing meningoencephalitis: a case report. The Lancet. 2016. 10.1016/S0140-6736(16)30386-5 PMC4967715 27209148

[23] Rabelo I, Lee V, Fallah M, Massaquoi M, Evlampidou I, Crestani R, Decroo T, Van den Bergh R, Severy N. Psychological distress among Ebola survivors discharged from an Ebola Treatment Unit in Monrovia, Liberia- A qualitative study. Frontiers in Public Health. 2016; 4: 142 doi: 10.3389/fpubh.2016.00142 27458576 10.3389/fpubh.2016.00142 PMC4931229

[24] Reardon S. Ebola's mental-health wounds linger in Africa. Nature. 2015;519(7541):13–4. doi: 10.1038/519013a 25739606 10.1038/519013a

[25] Mattia JG, Vandy MJ, Chang JC, Platt DE, Dierberg K, Bausch DG, et al Early clinical sequelae of Ebola virus disease in Sierra Leone: a cross-sectional study. The Lancet Infectious Diseases. 2016(3):331–8. doi: 10.1016/S1473-3099(15)00489-2 26725449 10.1016/S1473-3099(15)00489-2 PMC5617647

[26] Cham K. Sierra Leone announces semen testing programme. Africa Review. 2015 October 7.

[27] Burton J, Darbes LA, Operario D. Couples-focused behavioral interventions for prevention of HIV: Systematic review of the state of evidence. AIDS and behavior. 2010;14(1):1–10. doi: 10.1007/s10461-008-9471-4 18843530 10.1007/s10461-008-9471-4 PMC3640442

[28] Soka M, Choi M, Baller A, White S, Rogers E, Purpura L, et al Preventing sexual transmission of Ebola virus: Preliminary findings from the first National Semen Testing and Counseling Program for Ebola Survivors-Liberia. LANCET Global Health. 2016.

[29] Keita AK, Toure A, Sow MS, Raoul H, Magassouba N, Delaporte E, et al Extraordinary long-term and fluctuating persistence of Ebola virus RNA in semen of survivors in Guinea: implications for public health. Clin Microbiol Infect. 2016. 10.1016/j.cmi.2016.11.005 27866862

[30] Sissoko D, Duraffour S, Kerber R, Kolie JS, Beavogui AH, Camara A-M, et al Persistence and clearance of Ebola virus RNA from seminal fluid of Ebola virus disease survivors: a longitudinal analysis and modelling study. The Lancet Global Health. 2017;5(1):e80–e8. doi: 10.1016/S2214-109X(16)30243-1 27955791 10.1016/S2214-109X(16)30243-1

[31] Fischer WA, Wohl DA. Confronting Ebola as a Sexually Transmitted Infection. Clinical Infectious Diseases. 2016. 10.1093/cid/ciw123 PMC4845792 26936667

[32] Oster AM. B J, Styker JE., et al Interim Guidelines for Prevention of Sexual Transmission of Zika Virus- United States. MMWR. 2016:120–1. doi: 10.15585/mmwr.mm6505e1 26866485 10.15585/mmwr.mm6505e1

