# Supplementary material for: Development of risk reduction behavioral counseling for Ebola virus disease survivors enrolled in the Sierra Leone Ebola Virus Persistence Study, 2015-2016
Source: PLoS Negl Trop Dis. 2017 Sep 11;11(9):e0005827. doi: 10.1371/journal.pntd.0005827 (PMC5593175; doi:10.1371/journal.pntd.0005827)
Supplement: S1 Checklist — (DOC) [file pntd.0005827.s001.doc]

STROBE Statement—Checklist of items that should be included in reports of ***cohort studies***

|  | Item No | Recommendation |
| --- | --- | --- |
| **Title and abstract** | 1 | (*a*) Indicate the study’s design with a commonly used term in the title or the abstract  **SEE PAGE 5-6 LINE 111-121.**  The study consisted of two phases: the first phase assessed EBOV persistence in semen of 100 adult male EVD survivors; the second phase assessed EBOV persistence in semen and additional body fluids (vaginal fluid, menstrual blood, urine, rectal fluid, sweat, saliva, tears, and breast milk as applicable by sex) in 120 male and 120 female EVD survivors. Male and female EVD survivors living with HIV were also invited to participate in the study to characterize EBOV persistence among EVD survivors living with HIV. The study took place in two sites: Military Hospital 34 (an urban facility in Freetown, Western District) and Lungi Government Hospital (a semi-rural facility in Lungi, Port Loko District). In this paper, we discuss the development and implementation of the Ebola Virus Persistence Risk Reduction Behavioral Counseling Protocol (henceforth referred to as the behavioral counseling protocol) used in the study. |
|  |  | (*b*) Provide in the abstract an informative and balanced summary of what was done and what was found  **SEE PAGE 2 LINE 42-53**  **Methodology/Principal Findings:** The Ebola Virus Persistence Risk Reduction Behavioral Counseling Protocol was developed from a framework used to prevent transmission of HIV and other sexually transmitted infections. The framework helped to identify barriers to risk reduction and facilitated the development of a personalized risk-reduction plan, particularly around condom use or abstinence. Pre-test and post-test counseling sessions included risk reduction guidance, and post-test counseling was based on the participants’ individual test results. The behavioral counseling protocol enabled study staff to translate the study’s body fluid test results into individualized information for study participants.  **Conclusions/Significance:** The Ebola Virus Persistence Risk Reduction Behavioral Counseling Protocol provided guidance to mitigate the risk of EBOV transmission from EVD survivors. It has since been shared with and adapted by other EVD survivor body fluid testing programs and studies in Ebola affected countries. |
| Introduction | | |
| Background/rationale | 2 | Explain the scientific background and rationale for the investigation being reported  **SEE PAGE 4 LINE 74-94**  Limited studies during four outbreaks prior to 2014 showed evidence of persistent Ebola virus (EBOV) in semen [2]. In the 1995 Kikwit outbreak in the Democratic Republic of Congo, viable virus in semen was detected 82 days post symptom onset and EBOV ribonucleic acid (RNA) was detected 101 days post symptom onset [3]. Less evidence was found for short-term EBOV persistence in vaginal and rectal fluids as well as urine and sweat [2]. In West Africa, EVD survivors who recovered from disease were initially advised by the World Health Organization (WHO) to abstain from sex or to use condoms for three months after discharge from an Ebola Treatment Unit (ETU) [4]. As the number of individuals who survived EVD in West Africa grew into the thousands, questions were raised regarding EBOV persistence in semen and in other body fluids of EVD survivors and potential implications of viral persistence on residual risk of EBOV transmission from EVD survivors beyond the original three month recommendations [2, 5, 6].  Given limited pre-existing data, the potential for EBOV to persist in survivors was identified as an important research topic early in the epidemic. There was also a programmatic need to develop methods to test body fluids of EVD survivors and counsel them on risk reduction practices. This need became even more clear when a woman from Liberia tested positive for EBOV infection, and epidemiologic investigation found that her only exposure was unprotected vaginal intercourse with a male EVD survivor whose semen tested positive for EBOV RNA by real time reverse transcriptase polymerase chain reaction (qRT-PCR) 199 days after he first became symptomatic with EVD [7, 8]. Sequencing of the RNA from the semen of the male EVD survivor closely matched the sequence recovered from the female patient’s blood, providing further evidence for male-to-female sexual transmission of EBOV long into convalescence [8]. |
| Objectives | 3 | State specific objectives, including any pre-specified hypotheses  **SEE PAGE 6 LINE 123-128**  The objectives of the behavioral counseling protocol within the study were to: (1) provide participants simple explanations of qRT-PCR and virus isolation testing and deliver individual test results; and (2) encourage participants to engage in risk reduction behavioral practices corresponding to their individual qRT-PCR test results until the participant received two consecutive negative qRT-PCR test results. Counselors also referred participants to available EVD survivor services in the community when necessary. |
| Methods | | |
| Study design | 4 | Present key elements of study design early in the paper  **SEE PAGE 5-6 LINE 111-121**  The study consisted of two phases: the first phase assessed EBOV persistence in semen of 100 adult male EVD survivors; the second phase assessed EBOV persistence in semen and additional body fluids (vaginal fluid, menstrual blood, urine, rectal fluid, sweat, saliva, tears, and breast milk as applicable by sex) in 120 male and 120 female EVD survivors. Male and female EVD survivors living with HIV were also invited to participate in the study to characterize EBOV persistence among EVD survivors living with HIV. The study took place in two sites: Military Hospital 34 (an urban facility in Freetown, Western District) and Lungi Government Hospital (a semi-rural facility in Lungi, Port Loko District). In this paper, we discuss the development and implementation of the Ebola Virus Persistence Risk Reduction Behavioral Counseling Protocol (henceforth referred to as the behavioral counseling protocol) used in the study.  **PAGE 7 LINE 164-167:** The standard operating procedure for the behavioral counseling protocol included EBOV persistence pre-and post-test counseling as well as HIV pre-and post-test counseling. EBOV persistence pre-test counseling is an introduction to EBOV testing and risk reduction advice prior to testing, while post-test counseling is a delivery of tailored guidance based on qRT-PCR test results.  **Please see Methods (Pages 6-15)** |
| Setting | 5 | Describe the setting, locations, and relevant dates, including periods of recruitment, exposure, follow-up, and data collection  **SEE PAGE 5, LINE 116-118**  The study took place in two sites: Military Hospital 34 (an urban facility in Freetown, Western District) and Lungi Government Hospital (a semi-rural facility in Lungi, Port Loko District).  **PAGE 7, Figure 1: Visit flow chart for Ebola Virus Persistence Risk Reduction Behavioral Counseling Protocol** |
| Participants | 6 | (*a*) Give the eligibility criteria, and the sources and methods of selection of participants. Describe methods of follow-up  **SEE PAGE 6, LINE 121**  The overall study design has been described elsewhere [5].  **Eligibility information in Implementation Paper:**  **SEE PAGE 11 LINE 237-241, (eligibility)**  **‘**EVD survivors aged 18 years or older, who held an ETU discharge certificate and photo identification were eligible for recruitment. Preferentially recruited survivors were those either most recently discharged, or least recently discharged from an ETU (i.e. recent and long term, not mid-term survivors), as well as any pregnant or lactating women. For the targeted recruitment of people living with HIV, the Network for HIV Positives in Sierra Leone was engaged, through collaboration with UNAIDS.**’**  **PAGE 11-13, LINE 242 – 282 (follow-up)**  **‘See Table 1 (Components of the different study visits); line 246 ’**  **LINE 248-9**  ‘Participants were discharged when semen/all body fluids tested qRT-PCR negative for EBOV twice consecutively during the initial set of visits, and once during the three and six month follow-up after initial discharge.’ |
|  |  | (*b*) For matched studies, give matching criteria and number of exposed and unexposed  **NOT APPLICABLE FOR THIS STUDY** |
| Variables | 7 | Clearly define all outcomes, exposures, predictors, potential confounders, and effect modifiers. Give diagnostic criteria, if applicable  **NOT APPLICABLE FOR THIS PAPER** |
| Data sources/ measurement | 8* | For each variable of interest, give sources of data and details of methods of assessment (measurement). Describe comparability of assessment methods if there is more than one group  **NOT APPLICABLE FOR THIS PAPER** |
| Bias | 9 | Describe any efforts to address potential sources of bias  **NOT APPLICABLE FOR THIS PAPER, WILL BE DISCUSSED IN SUBSEQUENT ‘RESULTS PAPERS’** |
| Study size | 10 | Explain how the study size was arrived at  **NOT APPLICABLE FOR THIS PAPER, WILL BE DISCUSSED IN SUBSEQUENT ‘RESULTS PAPERS’** |
| Quantitative variables | 11 | Explain how quantitative variables were handled in the analyses. If applicable, describe which groupings were chosen and why  **NOT APPLICABLE FOR THIS PAPER, WILL BE DISCUSSED IN SUBSEQUENT ‘RESULTS PAPERS’** |
| Statistical methods  **NOT APPLICABLE**  **FOR THIS PAPER, WILL BE DISCUSSED IN SUBSEQUENT ‘RESULTS PAPERS’** | 12 | (*a*) Describe all statistical methods, including those used to control for confounding |
|  |  | (*b*) Describe any methods used to examine subgroups and interactions |
|  |  | (*c*) Explain how missing data were addressed |
|  |  | (*d*) If applicable, explain how loss to follow-up was addressed |
|  |  | (*e*) Describe any sensitivity analyses |
| Results | | |
| Participants  **NOT APPLICABLE**  **FOR THIS PAPER, WILL BE DISCUSSED IN SUBSEQUENT ‘RESULTS PAPERS’** | 13* | (a) Report numbers of individuals at each stage of study—eg numbers potentially eligible, examined for eligibility, confirmed eligible, included in the study, completing follow-up, and analysed |
|  |  | (b) Give reasons for non-participation at each stage |
|  |  | (c) Consider use of a flow diagram |
| Descriptive data  **NOT APPLICABLE**  **FOR THIS PAPER, WILL BE DISCUSSED IN SUBSEQUENT ‘RESULTS PAPERS’** | 14* | (a) Give characteristics of study participants (eg demographic, clinical, social) and information on exposures and potential confounders |
|  |  | (b) Indicate number of participants with missing data for each variable of interest |
|  |  | (c) Summarise follow-up time (eg, average and total amount) |
| Outcome data **NA** | 15* | Report numbers of outcome events or summary measures over time |
| Main results  **NOT APPLICABLE**  **FOR THIS PAPER, WILL BE DISCUSSED IN SUBSEQUENT ‘RESULTS PAPERS’** | 16 | (*a*) Give unadjusted estimates and, if applicable, confounder-adjusted estimates and their precision (eg, 95% confidence interval). Make clear which confounders were adjusted for and why they were included |
|  |  | (*b*) Report category boundaries when continuous variables were categorized |
|  |  | (*c*) If relevant, consider translating estimates of relative risk into absolute risk for a meaningful time period |
| Other analyses  **NOT APPLICABLE** | 17 | Report other analyses done—eg analyses of subgroups and interactions, and sensitivity analyses |
| Discussion | | |
| Key results **NA** | 18 | Summarise key results with reference to study objectives |
| Limitations  **NOT APPLICABLE**  **FOR THIS PAPER, WILL BE DISCUSSED IN SUBSEQUENT ‘RESULTS PAPERS’** | 19 | Discuss limitations of the study, taking into account sources of potential bias or imprecision. Discuss both direction and magnitude of any potential bias |
| Interpretation  **NOT APPLICABLE**  **FOR THIS PAPER, WILL BE DISCUSSED IN SUBSEQUENT ‘RESULTS PAPERS’** | 20 | Give a cautious overall interpretation of results considering objectives, limitations, multiplicity of analyses, results from similar studies, and other relevant evidence |
| Generalisability  **NOT APPLICABLE** | 21 | Discuss the generalisability (external validity) of the study results |
| Other information | | |
| Funding | 22 | Give the source of funding and the role of the funders for the present study and, if applicable, for the original study on which the present article is based  **Disclaimer**  The funders had no role in study design, data collection and analysis, decision to publish, or preparation of the manuscript.  Funding provided by the World Health Organization, the United States Centers for Disease Control and Prevention, the Chinese Center for Disease Control and Prevention, and the Paul G. Allen Family Foundation (<http://www.pgafamilyfoundation.org/>). The World Health Organization gratefully acknowledges the financial contribution of the WHO's Ebola Response Programme, The Paul G. Allen Family Foundation and the UNDP-UNFPA-UNICEF-WHO-World Bank Special Programme of Research, Development and Research Training in Human Reproduction (HRP) in support of the Sierra Leone Ebola Virus Persistence Study. |

**Note:** An Explanation and Elaboration article discusses each checklist item and gives methodological background and published examples of transparent reporting. The STROBE checklist is best used in conjunction with this article (freely available on the Web sites of PLoS Medicine at http://www.plosmedicine.org/, Annals of Internal Medicine at http://www.annals.org/, and Epidemiology at http://www.epidem.com/). Information on the STROBE Initiative is available at http://www.strobe-statement.org.
