# Supplementary material for: Development of risk reduction behavioral counseling for Ebola virus disease survivors enrolled in the Sierra Leone Ebola Virus Persistence Study, 2015-2016
Source: PLoS Negl Trop Dis. 2017 Sep 11;11(9):e0005827. doi: 10.1371/journal.pntd.0005827 (PMC5593175; doi:10.1371/journal.pntd.0005827)
Supplement: S1 Protocol — (DOCX) [file pntd.0005827.s002.docx]

**sierra leone**

**ebola virus persistence study**

Ebola virus persistence risk Reduction Behavioral Counseling protocol

Table of Contents

[SEMEN 4](#_Toc489978327)

[SOP for Ebola Virus Persistence Study - Pre-test Counseling Session 4](#_Toc489978328)

[SEMEN 8](#_Toc489978329)

[SOP for Ebola Virus Persistence Study - Delivery of Test Results 8](#_Toc489978330)

[SEMEN 11](#_Toc489978331)

[SOP for Ebola Virus Persistence Study – Post-test Counselling 11](#_Toc489978332)

[VAGINAL FLUID**,** RECTAL FLUID**,** URINE**,** MENSTRUAL BLOOD 16](#_Toc489978333)

[SWEAT, TEARS, SALIVA, & BREAST MILK 16](#_Toc489978334)

[SOP for Ebola Virus Persistence Study - Pre-test Counseling Session 16](#_Toc489978335)

[VAGINAL FLUID**,** RECTAL FLUID**,** URINE**,** MENSTRUAL BLOOD 20](#_Toc489978336)

[SWEAT, TEARS, SALIVA, & BREAST MILK^1^ 20](#_Toc489978337)

[SOP for Ebola Virus Persistence Study – Delivery of Test Results 20](#_Toc489978338)

[VAGINAL FLUID**,** RECTAL FLUID**,** 24](#_Toc489978339)

[URINE & MENSTRUAL BLOOD^1^ 24](#_Toc489978340)

[SOP for Ebola Virus Persistence Study - Post-test Counselling 24](#_Toc489978341)

[SWEAT 32](#_Toc489978342)

[SOP for Ebola Virus Persistence Study - Post-test Counselling 32](#_Toc489978343)

[SALIVA 40](#_Toc489978344)

[SOP for Ebola Virus Persistence Study - Post-test Counselling 40](#_Toc489978345)

[TEARS 49](#_Toc489978346)

[SOP for Ebola Virus Persistence Study - Post-test Counselling 49](#_Toc489978347)

[BREAST MILK 54](#_Toc489978348)

[SOP for Ebola Virus Persistence Study - Post-test Counselling 54](#_Toc489978349)

[Appendix 1: IPC Guidance 62](#_Toc489978350)

[Appendix 2: Mango Tree Analogy for qRT-PCR Results 65](#_Toc489978351)

[Appendix 3: Condom Demonstration 66](#_Toc489978352)

[Appendix 4: HIV Testing^1^ 68](#_Toc489978353)

[HIV Pre-test Counseling for EVD Survivors 68](#_Toc489978354)

[HIV Post-test Counseling for EVD Survivors 68](#_Toc489978355)

[Appendix 5: Delivery of Pregnancy Test Results 73](#_Toc489978356)

[Appendix 6: BP100 Distribution for Pregnant and Lactation Women 74](#_Toc489978357)

[Appendix 7: Intimate Partner Violence (IPV) Script 76](#_Toc489978358)

[Appendix 8: Domestic Violence Referral Pathways 78](#_Toc489978359)

[Sierra Leone National Referral Protocol on Gender Based Violence 78](#_Toc489978360)

[Appendix 9: Delivery of Virus Isolation Results 81](#_Toc489978361)

[Appendix 10: Talking Points for Male Participants 84](#_Toc489978362)

[Appendix 11: Talking Points for Female Participants 95](#_Toc489978363)

SEMEN

# SOP for Ebola Virus Persistence Study - Pre-test Counseling Session

*[Counselors deliver script in native language]*

**Outline:**

1. Introduction of session
2. Discussion about sexual behavior and explanation of guidance
3. Negotiation of risk reduction
4. Condom demonstration
5. HIV testing
6. Referral to services
7. Concluding message

**Step 1: Introduction of Session**

Hello, my name is [*counselor’s name*]. Welcome [*patient’s name*] and thank you for coming in today to participate in the Sierra Leone Ebola Virus Persistence Study. I am a counselor working with Ebola survivors.

- What language do you prefer to speak in?

First, we are so happy that you have recovered from Ebola. It takes a strong person to survive Ebola.

- How are you doing since you have recovered?

[*If participant expresses suffering from Ebola*]: I am sorry for the pain you have experienced because of Ebola. Your experience sounds like it was difficult.

The purpose of our meeting today is so that I can give you information about the testing we will do to detect if pieces of the Ebola virus are in your semen. We will also talk about things you can do in your life to reduce the risk of passing Ebola to a person you have sex with. All the information that you and I discuss will be kept confidential. That means that we will not share your name or any information about you with people who are not on the research team. If you have any questions during our meeting, please don’t hesitate to ask me. If you have questions later, you can contact [*counselor name and contact information*]. You can also contact the study leader [*PI name and contact information*]. You can choose not to answer any question I ask.

We know that discussing sex can be uncomfortable. However, it is important to discuss sex with you so we can better understand how to help you and your sex partners stay safe. Your contribution to this study is important.

- Do you have any questions about Ebola or Ebola in your semen?

[*Pause to answer questions about Ebola, semen, etc.*]

If you do not wish to continue with the study, you can drop out at any time. If you choose to drop out, you can still use all Ebola survivor services here and at other Ebola survivor clinics. If you wish to withdraw from the study and not provide any additional samples, you will still have the opportunity to speak further about the test results and measures that can reduce the risk of transmitting Ebola to other people with our study team.

The purpose of our meeting today is so that I can give you information about the testing we will do to detect if pieces of the Ebola virus are in your semen.

The test that we are going to do is called RT-PCR. It will detect if there are pieces of the Ebola virus in your semen. It will not tell us if this virus can be detected or not.

[*Use the mango tree analogy to explain how the RT-PCR test works. See Appendix 2*].

When you get two test results in a row where pieces of the Ebola virus are not detected in your semen, you will be discharged from the study.

To make sure that you understand, let us look at a few examples of test results together.

**Example 1**

| **Visit** | **Test result** |
| --- | --- |
| 1 | Not detected |
| 2 | Detected |
| 3 | Not detected |

- If these are the test results, should the participant give another sample or is the participant discharged?

The correct answer is that the participant must give another sample because the two test results which were “not detected” were not in a row.

**Example 2**

| **Visit** | **Test result** |
| --- | --- |
| 1 | Detected |
| 2 | Not detected |
| 3 | Not detected |

- If these are the test results, should the participant give another sample or is the participant discharged?

The correct answer is that the participant is discharged from the study because we have two test results which were “not detected” in a row.

- Do you understand what we mean when we say we are looking for two test results in a row where pieces of the Ebola virus are not detected?

As we discussed, it might be possible for you to pass Ebola to a person you have sex with. Let’s talk more about that now.

**Step 2: Discussion about sexual behavior and explanation of guidance**

[*The purpose of these questions is to begin a conversation to understand the participant’s sexual risk behavior*]

I want to remind you that you do not have to answer anything you do not want to answer. Everything you say today will be confidential.

- What brought you in today to get your semen tested?
- Are you married, single, divorced, or separated?
- Since recovering from Ebola, have you had sex (manual, oral, vaginal, or anal)? Since recovering from Ebola, have you masturbated?
  - *[Please refer to IPC guidance in Appendix 1 and provide all of the guidance in the table for pre-test counselling for semen]*
- If you had sex after recovering from Ebola, were you able to use a condom all the times you had sex? If not, why?
- Do you know how to use a condom?
- What makes it difficult for you to use a condom?
- Have you ever used a condom before?
  - [*If yes*]: What is the difference between the times you used a condom and the times you didn’t use a condom?
- Is it more difficult to use condoms with some sexual partners than others? If yes, why?
- Does your religion allow you to use a condom?
- Does alcohol or other drugs make it difficult to use a condom?
- Where do you get your condoms?
- Do you have problems getting condoms?
- Are you and your partner trying to have a baby?
  - [*If yes*]: Until you have two test results in a row where pieces of the Ebola virus are not detected, we advise you not to try to have a baby.

**Step 3: Negotiation of risk reduction**

[*The purpose of this section is to ask the participant if they can be abstinent or use condoms until they get two test results in a row where pieces of the Ebola virus are not detected*].

It is good to think about safe sex. As we discussed, until you receive two test results in a row where pieces of the Ebola virus are not detected, it is safest for you to not have sex or if you decide to have sex, use a condom.

- Which option do you prefer?

[*If the participant has a history of not using condoms*]:

From what you have told me, there have been times where you have not used a condom. While we wait to find out if pieces of the Ebola virus are detected in your semen:

- Do you think you will be able to use a condom every time you have sex?
- Would you consider not having sex if you don’t want to use a condom?
- What makes it difficult for you to use a condom?
- Do you have any ideas how you can make it easier for you to use a condom?

Changing your behavior takes time and practice. If you are able to use a condom when you have sex, you will do something good for yourself and your sexual partner. Using a condom will keep the risk of passing Ebola to your sexual partner low.

**Step 4: Condom demonstration**

I will now perform a condom demonstration so that you know how to properly use a condom if you decide to have sex.

[*Perform condom demonstration and give participant condoms to take home. See Appendix 3*].

**Step 5: HIV testing:**

As part of this study, we are offering HIV testing as well today. All test results are confidential and this testing is completely optional.

*[Please refer to Appendix 4]*

**Step 6: Referral to Services:**

*[If the participant raises any medical concerns during the session, fill in the survivor clinic referral form and alert the receptionist to link the participant to the survivor clinic for further care].*

*[If necessary, refer the participant to mental health nurse]*

**Step 7: Concluding Message:**

There are a very small number of survivors who became very sick after they recovered from Ebola. Therefore, if you develop a fever, diarrhea, nausea/vomiting, please call 117 or contact [*doctor1*] or [*doctor2*], the medical doctors at [*hospital/clinic name*], by calling the study cell phone. This phone number is on your study ID card [*study mobile phone number*]. I can show you now where this number is found. The study team is here to help you connect to the right care.

Thank you for participating in this study. We are so happy that you recovered from Ebola. Being sick with Ebola must have been very difficult. We thank you for doing your part to help us better understand the virus.

SEMEN

# SOP for Ebola Virus Persistence Study - Delivery of Test Results

*[Counselors deliver script in native language]*

**Outline:**

1. Greetings
2. Delivery and Explanation of Test Results

| 1. RT-PCR **detected** test result |
| --- |
| 1. **First** RT-PCR **not detected** test result |
| 1. RT-PCR **indeterminate** test result |
| 1. RT-PCR **no interpretation** test result |
| 1. **Second** RT-PCR **not detected** test result |

1. Discussion of Continued Study Participation
2. Concluding comments

**Step 1: Greetings**

Hello, my name is [*name of counselor*], and I am going to deliver the results of the RT-PCR test we did on the semen sample that you submitted on [*date of sample collection*].

**Step 2: Delivery and Explanation of Test Results**

**Step 2A: RT-PCR detected test result**

The RT-PCR test detected pieces of the Ebola virus in the sample that you provided. Let me explain a little bit more about what your test results mean. It is possible that you may be able to pass Ebola to a person who has contact with your semen through activity like manual, oral, vaginal, and anal sex. So, we ask that you provide another sample to test today. When you receive two test results in a row where pieces of the Ebola virus are not detected, we can say that pieces of the Ebola virus were not found in your semen and you will be able to resume usual sexual activity. However, we still recommend the use of condoms to protect yourself from HIV, other STIs and unwanted pregnancy.

**Step 2B: First RT-PCR not detected test result**

The RT-PCR test did not detect pieces of the Ebola virus in the sample that you provided. Let me explain a little bit more about what your test results mean. We did not find pieces of the Ebola virus in your semen, however, we want to make certain that the test results are correct and there are no pieces of the Ebola virus in your semen. So, we ask that you provide another sample to test today. When you receive two test results in a row where pieces of the Ebola virus are not detected, we can say that pieces of the Ebola virus were not found in your semen and you will be able to resume usual sexual activity. However, we still recommend the use of condoms to protect yourself from HIV, other STIs and unwanted pregnancy.

**Step 2C: RT-PCR indeterminate test result**

The RT-PCR test could not tell if pieces of the Ebola virus could be detected in your semen because it was on the border between detected and not detected. So, we ask that you provide another sample to test today. When you receive two test results in a row where pieces of the Ebola virus are not detected, we can say that pieces of the Ebola virus were not found in your semen and you will be able to resume usual sexual activity. However, we still recommend the use of condoms to protect yourself from HIV, other STIs and unwanted pregnancy.

**Step 2D: RT-PCR no interpretation test result**

The RT-PCR test could not tell if pieces of the Ebola virus could be detected in the semen sample that you provided because the quality of the sample made it difficult to test. So, we ask that you provide another sample to test today. When you receive two test results in a row where pieces of the Ebola virus are not detected, we can say that pieces of the Ebola virus were not found in your semen and you will be able to resume usual sexual activity. However, we still recommend the use of condoms to protect yourself from HIV, other STIs and unwanted pregnancy.

**Step 2E: Second RT-PCR not detected test result**

The RT-PCR test did not detect pieces of the Ebola virus in the sample that you provided. Let me explain a little bit more about what your test results mean. We have now found evidence two times in a row that pieces of the Ebola virus cannot be detected in your semen. You no longer need to provide semen samples until your 3 and 6-month follow-up visits. You can now engage in usual sexual behavior activity. We would like to remind you that condoms can still be used to protect yourself from HIV, STIs, and unwanted pregnancy. *[Skip to step 4]*

**Step 3: Discussion of Continued Study Participation**

When you have two “not detected” test results in a row from all the body fluids we will collect and test, we will stop collecting your body fluids. It may be possible that the virus could still be present in the body even if the laboratory cannot detect it in your body fluids. There have been a very small number of survivors who became seriously ill after they recovered from Ebola. Therefore, if you develop a fever, diarrhea, nausea/vomiting, please call 117 or contact [*doctor 1*] or [*doctor 2*] by calling the study cell phone. This number is on your study ID card. I can show you now where this number is found [*show the participant the study cell phone number on the study ID card*]. The study team is here to support you and to connect you to the right care.

Later today, you and I will have an opportunity to speak further about these test results and things you can do to reduce the risk of passing Ebola to a person with whom you have sex.

Right now, I can take you to the nurse so you can provide another sample today for us to test. You will get the results of this test at your follow-up appointment in two weeks. We will keep testing you until we get two test results in a row where pieces of the Ebola virus are not detected. Do you feel comfortable continuing?

*[If yes]:*

*[Skip to Step 4]*

*[If no]:*

- *[Ask why the participant does not want to provide a sample. Discuss the importance of knowing whether pieces of the Ebola virus are in or not in his semen].*
- *[Counselor decides whether mental health referral is needed (see: SOP for Medical/Mental Health Referrals)*].
- [*If referral is refused, ask*]:
  - Can I follow up with you in a few days to see how you are doing?
  - *[Record contact information and call the participant within three days for wellness check-up].*
  - Wellness check-up phone call script:
    - How are you feeling? How are you feeling about the test results you received?
    - How are you feeling about providing an additional sample for us to submit for RT-PCR testing so we can make sure that pieces of the Ebola virus are not in your semen?
    - We have asked two people who have survived Ebola (1 male, 1 female) to talk to participants. Would you like to talk to them about how you are feeling? Can I give them your contact information?

**Step 4: Concluding comments**

[*If participant is discharged*]:

Thank you for your participation in this very important study. We are grateful for your services and believe that you have helped to improve the lives of other Ebola survivors.

[*If participant is continuing in the study*]:

Thank you for continuing your participation. I will now take you to the nurse to continue with the study.

SEMEN

# SOP for Ebola Virus Persistence Study – Post-test Counselling

*[Counselors deliver script in native language]*

**Outline:**

1. Greetings
2. Follow-up conversation about sexual health

| 1. RT-PCR **detected** test result |
| --- |
| 1. **First** RT-PCR **not detected** test result |
| 1. RT-PCR **indeterminate** test result |
| 1. RT-PCR **no interpretation** test result |

1. Negotiate sexual risk reduction steps
2. Negotiate risk reduction
3. Condom demonstration
4. HIV Testing
5. Referral to services
6. Concluding message

**Step 1: Greetings**

Hello, and thank you for coming to this counseling session.

Let us talk a bit about things you can do to keep you and people you have sex with safe as we wait for your next test results. You do not have to answer any question that you do not want to answer. Everything you tell me today is completely confidential. If you feel uncomfortable at any time, just let me know and we will move on. We are here to support you.

Let us talk about things you can do in your life to reduce the risk of passing Ebola to a sexual partner.

- Are you married, single, divorced, or separated? Has anything changed in your relationship since your last visit?
- Have you had sex with anyone since you came in for your last visit? Do you plan to have sex with anyone soon?

**Step 2: Follow-up conversation about sexual health**

**Step 2A: RT-PCR detected test result**

*[If yes]*:

Thank you for telling me. Because we detected pieces of Ebola in your semen, we strongly recommend that you do not have sex (manual, oral, vaginal, and anal) until you have two test results in a row where pieces of the Ebola virus are not detected in your semen. If you want to have sex, we ask that you use a condom. This will make the chance of spreading Ebola as low as possible. What do you think about this?

*[If no]*:

Thank you for telling me. Because we detected pieces of the Ebola virus in your semen, we strongly recommend that you do not have sex (manual, oral, vaginal, and anal) until you receive two test results in a row where pieces of the Ebola virus are not detected in your semen. Not having sex is the safest thing you can do right now. Do you think it will be possible for you to not have sex or if you have sex, to use condoms in the future? What do you think about this?

*[Please provide all appropriate IPC guidance according to Appendix 1]*

**Step 2B: First RT-PCR not detected test result**

*[If yes]*:

Thank you for telling me. Even though your test result today did not detect pieces of the Ebola virus were detected in your semen at the time you were tested, we strongly recommend that you do not have sex (manual, oral, vaginal, and anal) until you receive two test results in a row where pieces of the Ebola virus are not detected in your semen. If you want to have sex, we ask that you use condoms. This will make sure that the chance of spreading Ebola will be as low as possible. What do you think about this?

*[If no]*:

Thank you for telling me. Even though your test result today did not detect pieces of the Ebola virus were detected in your semen at the time you were tested, we strongly recommend that you do not have sex (manual, oral, vaginal, and anal) today until you receive two test results in a row where pieces of the Ebola virus are not detected in your semen. Not having sex is the safest thing you can do right now. Do you think it will be possible for you to not have sex or if you have sex, to use condoms in the future? What do you think about this?

*[Please provide all appropriate IPC guidance according to Appendix 1]*

**Step 2C: RT-PCR indeterminate test result**

*[If yes]*:

Thank you for telling me. We strongly recommend that you do not have sex (manual, oral, vaginal, and anal) until you have two test results in a row where pieces of the Ebola virus are not detected in your semen. If you want to have sex, we ask that you use condoms. This will make sure that the chance of spreading Ebola will be as low as possible. What do you think about this?

*[If no]*:

Thank you for telling me. We strongly recommend that you do not have sex (manual, oral, vaginal, and anal) until you receive two test results in a row where pieces of the Ebola virus are not detected in your semen. Not having sex is the safest thing you can do right now. Do you think it will be possible for you to not have sex or if you have sex, to use condoms in the future? What do you think about this?

*[Please provide all appropriate IPC guidance according to Appendix 1]*

**Step 2D: RT-PCR no interpretation test result**

*[If yes]*:

Thank you for telling me. We strongly recommend that you do not have sex (manual, oral, vaginal, and anal) until you have two test results in a row where pieces of the Ebola virus are not detected in your semen. If you want to have sex, we ask that you use condoms. This will make sure that the chance of spreading Ebola will be as low as possible. What do you think about this?

*[If no]*:

Thank you for telling me. We strongly recommend that you do not have sex (manual, oral, vaginal, and anal) until you receive two test results in a row where pieces of the Ebola virus are not detected in your semen. Not having sex is the safest thing you can do right now. Do you think it will be possible for you to not have sex or if you have sex, to use condoms in the future? What do you think about this?

*[Please provide all appropriate IPC guidance according to Appendix 1]*

**Step 3: Negotiate Sexual Risk Reduction Steps**

Let’s talk a bit about your life since the last time we talked. I want to remind you that you do not have to answer anything you do not want to answer. Everything you say today will remain confidential.

*[If you had sex]*:

- Who did you have sex with?
- Were you able to use a condom all of the times you had sex? If not, what happened?
- What is your religion? Does your religion allow you to use a condom?
- Tell me about the things that made it more challenging for you to use a condom.
- What was the difference between the times you used a condom and the times you didn’t?
- How did alcohol and other drugs affect your decision to have sex without a condom?
- What was it about some partners that made it more difficult to use a condom?
- Did you have access to condoms?

**Step 4: Negotiate Risk Reduction**

**If the participant did not have sex (low risk):**

It is great that you chose to wait for your test results before you had sex.

Do you think you will be able to not have sex until you get the next test results? If you are planning to have sex, do you think you will be able to use a condom during sex?

- What might get in the way of you using a condom?
- How could you plan for that challenge and work around it?
- How would you feel if you could complete this step?

**If the participant had sex but did not use a condom (high risk):**

It is great that you are thinking about safe sex. From what you have told me, there were some times when you did not use a condom.

- While we are waiting for your next test results, do you think you will be able to use a condom during sex?
  - Would you consider not having sex at all if you don’t want to use a condom?
  - Do you feel comfortable using a condom? How can we help make you more comfortable?
  - What might get in the way of you using a condom?
  - How could you plan for that challenge and work around it?
  - How would you feel if you could complete this step?

**If the participant had sex and used a condom (low risk):**

It is great that you used a condom to practice safe sex. You have helped to protect your partner.

- While we are waiting for your next test results, do you think you will be able to keep using condoms during sex?
- What might get in the way of you using a condom?
- How could you plan for that challenge and work around it?
- How would you feel if you could complete this step?

Changing behavior takes time and practice. You will have done something good for yourself and for this Ebola fight by trying out this step.

**Step 5: Condom Demonstration**

*[Offer condom demonstration to participant. Please refer to Appendix 3]*

*[Give each participant 35 condoms]*

**Step 6: HIV testing**

As part of this study, we are offering HIV testing as well today. All results are confidential and this testing is completely optional. Would you like to hear more about the HIV test today?

*[If no, tell them the test will be available next time if they change their mind].*

*[If yes, please refer to Appendix 4]]:*

**Step 7: Referral to Services**

*[If the participant raises any medical concerns during the session, fill in the survivor clinic referral form and alert the receptionist to link the participant to the survivor clinic for further care].*

*[If is necessary, refer the participant to mental health nurse].*

**Step 8: Concluding Message:**

Thank you for participating in this study. We are so happy that you recovered from Ebola. Being sick with Ebola must have been very difficult, and we thank you for doing your part to help us better understand the virus. Do you have any questions for us at this time?

VAGINAL FLUID**,** RECTAL FLUID**,** URINE**,** MENSTRUAL BLOOD

SWEAT, TEARS, SALIVA, & BREAST MILK^[[1]](#footnote-1)^

# SOP for Ebola Virus Persistence Study - Pre-test Counseling Session

*[Counselors deliver script in native language]*

**Outline:**

1. Introduction of session
2. Explanation of guidance
3. Condom demonstration
4. HIV testing
5. Delivery of pregnancy test results (if test performed)
6. BP100 distribution (for pregnant and lactating women)
7. Referral to services
8. Concluding message

**Step 1: Introduction of session**

Hello, my name is [*counselor’s name*]. Welcome [*patient’s name*] and thank you for coming in today to participate in the Sierra Leone Ebola Virus Persistence Study. I am a counselor working with Ebola survivors.

- What language do you prefer to speak in?

First, we are so happy that you have recovered from Ebola. It takes a strong person to survive Ebola. How are you doing since you have recovered?

*[If participant expresses suffering after recovering from Ebola, you can say]*: I am sorry for the pain you have experienced because of Ebola. Your experience sounds like it was difficult.

Let me explain why we are here today. We want to find out more about how the Ebola virus has affected people who have recovered from it. We especially want to know if Ebola can live in different fluids in a person’s body after they have recovered from the disease.

You do not have to answer any question that you do not want to answer. Everything you tell me today is completely confidential. If you feel uncomfortable at any time, just let me know and we will move on. We are here to support you.

Your contribution to this study is important and we are very grateful that you have come in today to talk about your participation in this study.

- Do you have any questions about Ebola before we go on?

*[Pause to answer any questions]*

The purpose of our meeting today is so that I can give you information about the testing we will do to detect if Ebola virus is in your body fluids.

All the information discussed will be kept confidential, that means that we will not share your name or any information about you with people not on the research team. If you have any questions during our meeting, please don’t hesitate to ask me. If you have questions later, you can contact [*counselor name and contact information*]. You can also contact the study leader [*PI name and contact information*]. You can choose not to answer any question I ask. If you do not wish to continue with the study, you can drop out at any time. If you choose to drop out you can still use all survivor services here and at other survivor clinics. If you wish to withdraw from the study and not provide any additional samples, you will still have the opportunity to speak further about the test results and measures that can reduce the risk of transmitting Ebola to other persons with our study team.

The purpose of our meeting today is so that I can give you information about the testing we will do to detect if pieces of the Ebola virus are in your *[body fluid]*.

The test that we are going to do is called RT-PCR. It will detect if there are pieces of the Ebola virus in your *[body fluid]*. It will not tell us if this virus can be detected or not.

[*Use the mango tree analogy to explain how the RT-PCR test works. See Appendix 2*].

When you get two test results in a row where pieces of the Ebola virus are not detected in your *[body fluid]*, you will be discharged from the study.

To make sure that you understand, let us look at a few examples of test results together.

**Example 1**

| **Visit** | **Test result** |
| --- | --- |
| 1 | Not detected |
| 2 | Detected |
| 3 | Not detected |

- If these are the test results, should the participant give another sample or is the participant discharged?

The correct answer is that the participant must give another sample because the two test results which were “not detected” were not in a row.

**Example 2**

| **Visit** | **Test result** |
| --- | --- |
| 1 | Detected |
| 2 | Not detected |
| 3 | Not detected |

- If these are the test results, should the participant give another sample or is the participant discharged?

The correct answer is that the participant is discharged from the study because we have two test results which were “not detected” in a row.

- Do you understand what we mean when we say we are looking for two test results in a row where pieces of the Ebola virus are not detected?

**Step 2: Explanation of guidance:**

In this study, we will use a laboratory test to look for pieces of Ebola virus in the different body fluids. We will be asking you to provide samples of fluids from different parts of your body.

We will offer you Ebola tests until there are two test results in a row where pieces of the Ebola virus are not detected in each of the different body fluids. Receiving your test results is optional and the choice is entirely up to you.

At this time, you do not need to do anything different from what you are doing now. If the test does detect pieces of Ebola virus in any of your bodily fluids, we will give you more information when we go over the test results with you.

When you have two test results in a row where pieces of the Ebola virus are not detected from all the body fluids we will collect and test, we will stop collecting your body fluids. It may be possible that the virus could still be present in the body even if the laboratory cannot detect it in your body fluids.

**Step 3: Condom demonstration:**

I will now perform a condom demonstration so that you know how to properly use a condom if you decide to have sex. We recommend that you use a condom to prevent HIV, other STIs and unwanted pregnancy.

[*Perform condom demonstration and give participant condoms to take home. See Appendix 3*].

**Step 4: HIV testing:**

As part of this study, we are offering HIV testing as well today. All test results are confidential and this testing is completely optional.

*[Please refer to Appendix 4]*

**Step 5: Delivery of pregnancy test results (if test performed)**

[*See Appendix 5*]

**Step 6: BP100 distribution (for pregnant and lactating women)**

[*See Appendix 6*]

**Step 7: Referral to Services:**

*[If the participant raises any medical concerns during the session, fill in the survivor clinic referral form and alert the receptionist to link the participant to the survivor clinic for further care].*

*[If necessary, refer the participant to mental health nurse].*

**Step 8: Concluding Message:**

There have been a very small number of survivors who became seriously ill after they recovered from Ebola. Therefore, if you develop a fever, diarrhea, nausea/vomiting, please call 117 or contact *[doctor 1]* or *[doctor 2]*, the medical doctors at the survivor clinic, by calling the study cell phone. This number is on your study ID card. I can show you now where this number is found. The study team is here to support you and to connect you to the right care.

Thank you for participating in this study. We are so happy that you recovered from Ebola. Being sick with Ebola must have been very difficult, and we thank you for doing your part to help us better understand the virus.

- Do you have any questions for us at this time?

VAGINAL FLUID**,** RECTAL FLUID**,** URINE**,** MENSTRUAL BLOOD

SWEAT, TEARS, SALIVA, & BREAST MILK^1^

# SOP for Ebola Virus Persistence Study – Delivery of Test Results

*[Counselors deliver script in native language]*

**Outline:**

1. Greetings
2. Delivery and Explanation of Test Results

| 1. RT-PCR **detected** test result |
| --- |
| 1. **First** RT-PCR **not detected** test result |
| 1. RT-PCR **indeterminate** test result |
| 1. RT-PCR **no interpretation** test result |
| 1. **Second** RT-PCR **not detected** test result |

1. Discussion of Continued Study Participation
2. Concluding message

**Step 1: Greetings:**

Hello, my name is [*name of counselor*], and I am going to deliver the results of the RT-PCR test we did on the *[body fluid]* sample that you submitted on [*date of sample collection*].

**Step 2: Delivery and Explanation of Test Results:**

**Step 2A: RT-PCR-Detected Result:**

The RT-PCR test detected pieces of the Ebola virus in the *[body fluid]* sample that you provided. Let me explain a little bit more about what your test results mean:

It is possible that you may be able to pass Ebola to a person who comes in contact with your *[body fluid]*.

*[For vaginal fluid, rectal fluid, urine, menstrual blood, and sweat]:* It possible that you may pass Ebola to someone through sexual activity, including manual, oral, vaginal, and anal sex.

*[For saliva]:* It possible that you may pass Ebola to someone through sexual activity, including oral sex and kissing.

So, we ask that you provide another sample to test today. When you receive two test results in a row where pieces of the Ebola virus are not detected, we can say that pieces of the Ebola virus were not found in your *[body fluid]* and you will be able to resume usual sexual activity. However, we still recommend the use of condoms to protect yourself from HIV, other STIs and unwanted pregnancy.

**Step 2B: First RT-PCR not detected test result**

The RT-PCR test did not detect pieces of the Ebola virus in the *[body fluid]* sample that you provided. Let me explain a little bit more about what your test results mean. We did not find pieces of the Ebola virus in your *[body fluid]*, however, we want to make certain that the test results are correct and there are no pieces of the Ebola virus in your *[body fluid]*. So, we ask that you provide another *[body fluid]* sample to test today. When you receive two test results in a row where pieces of the Ebola virus are not detected, we can say that pieces of the Ebola virus were not found in your *[body fluid]*. We do still recommend the use of condoms to protect yourself from HIV, other STIs and unwanted pregnancy.

**Step 2C: RT-PCR indeterminate test result**

The RT-PCR test could not tell if pieces of the Ebola virus could be detected in your *[body fluid]* because it was on the border between detected and not detected.

It is possible that you may be able to pass Ebola to a person who comes in contact with your *[body fluid]*.

*[For vaginal fluid, rectal fluid, urine, menstrual blood, and sweat]:* It possible that you may pass Ebola to someone through sexual activity, including manual, oral, vaginal, and anal sex.

*[For saliva]:* It possible that you may pass Ebola to someone through sexual activity, including oral sex and kissing.

So, we ask that you provide another *[body fluid]* sample to test today. When you receive two test results in a row where pieces of the Ebola virus are not detected, we can say that pieces of the Ebola virus were not found in your *[body fluid]*. We do still recommend the use of condoms to protect yourself from HIV, other STIs and unwanted pregnancy.

**Step 2D: RT-PCR no interpretation test result**

The RT-PCR test could not tell if pieces of the Ebola virus could be detected in the *[body fluid]* sample that you provided because the quality of the sample made it difficult to test. So, we ask that you provide another *[body fluid]* sample to test today. When you receive two test results in a row where pieces of the Ebola virus are not detected, we can say that pieces of the Ebola virus were not found in your *[body fluid]*. We do still recommend the use of condoms to protect yourself from HIV, other STIs and unwanted pregnancy.

**Step 2E: Second RT-PCR not detected test result**

The RT-PCR test did not detect pieces of the Ebola virus in the *[body fluid]* sample that you provided. Let me explain a little bit more about what your test results mean. We have now found evidence two times in a row that pieces of the Ebola virus cannot be detected in your *[body fluid]*. You no longer need to provide *[body fluid]* samples until your 3 and 6-month follow-up visits. You can now engage in usual sexual behavior activity. We would like to remind you that condoms can still be used to protect yourself from HIV, STIs, and unwanted pregnancy. *[Skip to step 4]*

**Step 3: Discussion of Continued Study Participation**

When you have two “not detected” test results in a row from all the body fluids we will collect and test, we will stop collecting your body fluids. It may be possible that the virus could still be present in the body even if the laboratory cannot detect it in your body fluids. There have been a very small number of survivors who became seriously ill after they recovered from Ebola. Therefore, if you develop a fever, diarrhea, nausea/vomiting, please call 117 or contact [*doctor 1*] or [*doctor 2*] by calling the study cell phone. This number is on your study ID card. I can show you now where this number is found [*show the participant the study cell phone number on the study ID card*]. The study team is here to support you and to connect you to the right care.

Later today, you and I will have an opportunity to speak further about these test results and things you can do to reduce the risk of passing Ebola to another person, including sexual activity.

Right now, I can take you to the nurse so you can provide another *[body fluid]* sample today for us to test. You will get the results of this test at your follow-up appointment in two weeks. We will keep testing you until we get two test results in a row where pieces of the Ebola virus are not detected in all your body fluids that we are collecting. Do you feel comfortable continuing?

*[If yes]:*

*[Skip to Step 4]*

*[If no]:*

- *[Ask why the participant does not want to provide a sample. Discuss the importance of knowing whether pieces of the Ebola virus are in or not in his/her body fluid].*
- *[Counselor decides whether mental health referral is needed (see: SOP for Medical/Mental Health Referrals)*].
- [*If referral is refused, ask*]:
  - Can I follow up with you in a few days to see how you are doing?
  - *[Record contact information and call the participant within three days for wellness check-up].*
  - Wellness check-up phone call script:
    - How are you feeling? How are you feeling about the test results you received?
    - How are you feeling about providing an additional sample for us to submit for RT-PCR testing so we can make sure that pieces of the Ebola virus are not in your *[body fluid]*?
    - We have asked two people who have survived Ebola themselves (1 male, 1 female) to talk to participants. Would you like to talk to them about how you are feeling? Can I give them your contact information?

**Step 4: Concluding message**

[*If participant is discharged*]:

Thank you for your participation in this very important study. We are grateful for your services and believe that you have helped to improve the lives of other Ebola survivors.

[*If participant is continuing in the study*]:

Thank you for continuing your participation. I will now take you to the nurse to continue with the study.

VAGINAL FLUID**,** RECTAL FLUID**,**

URINE & MENSTRUAL BLOOD^1^

# SOP for Ebola Virus Persistence Study - Post-test Counselling

*[Counselors deliver script in native language]*

**Outline:**

1. Greetings

**Detected and Indeterminate Test Result**

1. Follow-up on risk behavior conversation

2A: Detected test result

2B: Indeterminate test result

1. Negotiate sexual risk reduction steps
2. Negotiate risk reduction

**Not Detected Test Result**

1. Follow-up on risk behavior conversation

2C. Not detected test result

2D. No interpretation test result

1. Negotiate sexual risk reduction steps
2. Negotiate risk reduction

**No Interpretation Test Result**

1. Follow-up on risk behavior conversation

2D. No Interpretation test result

1. Condom distribution (demonstration if necessary)
2. HIV testing
3. Delivery of pregnancy test results (if test performed)
4. BP100 distribution (for pregnant and lactating women)
5. Referral to services
6. Concluding message

**Step 1: Greetings:**

Hello, and thank you for coming to this counseling session.

**Step 2: Follow-up risk behavior conversation**

**Step 2A: Detected test result**

As you know, the RT-PCR test detected pieces of the Ebola virus in the *[body fluid]* sample that you provided.

The pieces of the Ebola virus will one day leave your *[body fluid]*. We will keep testing you until we get two test results in a row where pieces of the Ebola virus are not detected in your *[body fluid]*. We would like you to continue to participate in this study by coming back in two weeks to give another sample to us. How do you feel about this? Do you have any questions?

*[Pause to give participant chance to discuss their feelings/emotions]*

Let me explain a little bit more about what your test results mean.

Because we found pieces of the Ebola virus in your *[body fluid]*, we recommend you follow some basic guidance to lower the risk of passing Ebola to other people until you get two test results in a row where pieces of the Ebola virus are not detected in your *[body fluid]*.

**Step 2B: Indeterminate test result**

As you know, the RT-PCR test could not tell if the Ebola virus in the *[body fluid]* sample that you provided. This is because your sample was on the border between detected and not detected. We will keep testing you until we get two test results in a row where pieces of the Ebola virus are not detected in your *[body fluid]*. We would like you to continue to participate in this study by coming back in two weeks to give another sample to us. How do you feel about this? Do you have any questions?

*[Pause to give participant chance to discuss their feelings/emotions]*

Let me explain a little bit more about what your test results mean.

We recommend you follow some basic guidance to lower the risk of passing Ebola to other people until you get two test results in a row where pieces of the Ebola virus are not detected in your *[body fluid]*.

*[Please refer to IPC guidance in Appendix 1 and provide guidance to the participant]*

Let’s talk a bit about things you can do to keep you and people you have sex with safe as we wait for your next test results. You do not have to answer any question that you do not want to answer. Everything you tell me today is completely confidential. If you feel uncomfortable at any time, just let me know and we will move on. We are here to support you.

Let us chat a bit about things you can do in your life to reduce the risk of passing Ebola to a partner.

- Are you married, single, divorced, separated? Has anything changed in your relationship since your last visit?
- Have you had sex with anyone since you came in for your last visit? Do you plan on having sex with anyone soon?

**Detected test result**

*[If yes]:*

Thank you for telling me. Because we found pieces of the Ebola virus in your *[body fluid]*, we strongly recommend that you do not have sex (manual, oral, vaginal, and anal) until you receive two test results in a row where pieces of the Ebola virus are not detected in your *[body fluid]*. If you want to have sex, we ask that you or your partner use a male or female condom. This will make certain that the chance of spreading Ebola will be as low as possible. What do you think about this?

*[If no]*:

Thank you for telling me. Because we found pieces of the Ebola virus in your *[body fluid]*, we strongly recommend that you do not have sex (manual, oral, vaginal, and anal) until you receive two test results in a row where pieces of the Ebola virus are not detected in your *[body fluid]*. Not having sex is the safest thing you can do right now. Do you think it will be possible for you to not have sex or use male or female condoms in the future?

**Indeterminate test result**

*[If yes]:*

Thank you for telling me. We strongly recommend that you do not have sex (manual, oral, vaginal, and anal) until you receive two test results in a row where pieces of the Ebola virus are not detected in your *[body fluid]*. If you want to have sex, we ask that you or your partner use a male or female condom. This will make certain that the chance of spreading Ebola will be as low as possible. What do you think about this?

*[If no]*:

Thank you for telling me. We strongly recommend that you do not have sex (manual, oral, vaginal, and anal) until you receive two test results in a row where pieces of the Ebola virus are not detected in your *[body fluid]*. Not having sex is the safest thing you can do right now. Do you think it will be possible for you to not have sex at all, or if you do have sex, to use male or female condoms?

**Step 3: Negotiate Sexual Risk Reduction Steps**

Let’s talk a bit about your life since the last time we talked. I want to remind you that you do not have to answer anything you do not want to answer. Everything you say today will remain confidential.

*[If you had sex]*:

- Who did you have sex with?
- Were you able to use a condom all of the times you had sex? If not, what happened?
- What is your religion? Does your religion allow you to use a condom?
- Tell me about the things that made it more challenging for you to use a condom.
- What was the difference between the times you used a condom and the times you didn’t?
- How did alcohol and other drugs affect your decision to have sex without a condom?
- What was it about some partners that made it more difficult to use a condom?
- Did you have access to condoms?

*[For female participants only]:*

- Are you and your partner trying to have a baby?

*[If yes]:*

I understand that it is important to you and your partner to have a baby. However, not having sex, or using a condom during sex, is the safest thing for you and your partner right now. Because we know that Ebola may live in your *[body fluid]*, we ask that you use a condom every time you have sex until you have two test results in a row where Ebola is not detected.

Are you currently using any form of contraception to protect yourself from pregnancy, like tablets, an injection, condoms, or an implant?

*[If participant is using hormonal contraception only and not barrier contraception, conduct counseling on dual protection.]*

I want to remind you that using contraception like an injection, tablets, or an implant can only protect you from pregnancy and cannot protect you from sexually transmitted infections. Only condoms or abstinence can protect you from sexually transmitted infections. To fully protect yourself and keep yourself safe from both pregnancy and sexually transmitted infections, we encourage you to use condoms even if you are also using other methods to protect yourself from pregnancy.

Now I would like to talk a bit about your relationships. Tell me a bit about how things are going with your partner:

1. How do you feel about your relationship?
2. How do you feel about the way that your partner treats you?

*[Allow the participant ample time to respond]*

- How do you think your partner or other people in your life would react if they knew you received a test result where pieces of the Ebola virus were detected in your *[body fluid]*?

*[Probe further for possible intimate partner violence (IPV)* ***only if suspected based on responses to the above.*** *If IPV is suspected, please refer to IPV script and referral pathway in Appendix 7 & 8].*

**Step 4: Negotiate Risk Reduction**

**If the participant did not have sex (low risk):**

It is great that you chose to wait for your test results before you had sex.

Do you think you will be able to not have sex until you get the next test results? If you are planning to have sex, do you think you will be able to use a condom during sex?

- What might get in the way of you using a condom?
- How could you plan for that challenge and work around it?
- How would you feel if you could complete this step?

**If the participant had sex but did not use a condom (high risk):**

It is great that you are thinking about safe sex. From what you have told me, there were some situations where you did not use a condom.

- While we are waiting for your next test results, do you think you will be able to use a condom during sex?
  - Would you consider not having sex at all if you don’t want to use a condom?
  - Do you feel comfortable using a condom? How can we help make you more comfortable?
  - What might get in the way of you using a condom?
  - How could you plan for that challenge and work around it?
  - How would you feel if you could complete this step?

**If the participant had sex and used a condom (low risk):**

It is great that you used a condom to practice safe sex. You have helped to protect your partner.

- While we are waiting for your next test results, do you think you will be able to continue to use a condom during sex?
- What might get in the way of you using a condom?
- How could you plan for that challenge and work around it?
- How would you feel if you could complete this step?

Changing behavior takes time and practice. You will have done something good for yourself and for this Ebola fight by trying out this step.

**Step 2C: “Not detected” test result**

As you know, the RT-PCR test did not detect pieces of the Ebola virus in the *[body fluid]* sample that you provided. We will keep testing you until we get two test results in a row where pieces of the Ebola virus are not detected in your *[body fluid]*. We would like you to continue to participate in this study by coming back in two weeks to give another sample to us. How do you feel about this? Do you have any questions?

*[Pause to give participant chance to discuss their feelings/emotions]*

Let’s talk a bit about things you can do to keep you and people around you safe as we wait for your next test results. You do not have to answer any question that you do not want to answer. Everything you tell me today is completely confidential. If you feel uncomfortable at any time, just let me know and we will move on. We are here to support you.

Let us chat a bit about things you can do in your life to avoid HIV, other STIs, and unwanted pregnancy.

- Are you married, single, divorced, separated? Has anything changed in your relationship since your last visit?
- Have you had sex with anyone since you came in for your last visit? Do you plan on having sex with anyone soon?

Thank you for telling me. We strongly recommend that you use a condom if you have sex (manual, oral, vaginal, and anal). This will make certain that the chance of spreading HIV and other STIs will be as low as possible. What do you think about this?

**Step 3: Negotiate Sexual Risk Reduction Steps**

Let’s talk a bit about your life since the last time we talked. I want to remind you that you do not have to answer anything you do not want to answer. Everything you say today will remain confidential.

*[If you had sex]*:

- Who did you have sex with?
- Were you able to use a condom all of the times you had sex? If not, what happened?
- What is your religion? Does your religion allow you to use a condom?
- Tell me about the things that made it more challenging for you to use a condom.
- What was the difference between the times you used a condom and the times you didn’t?
- How did alcohol and other drugs affect your decision to have sex without a condom?
- What was it about some partners that made it more difficult to use a condom?
- Did you have access to condoms?

Now I would like to talk a bit about your relationships. Tell me a bit about how things are going with your partner:

- How do you feel about your relationship?
- How do you feel about the way that your partner treats you?

*[Allow the participant ample time to respond]*

- How do you think your partner or other people in your life would react if you had received a test result where pieces of the Ebola virus were detected in your *[body fluid]*?

*[Probe further for possible intimate partner violence (IPV)* ***only if suspected based on responses to the above.*** *Refer to Appendix 7 and 8].*

**Step 4: Negotiate Risk Reduction**

**If the participant did not have sex (low risk):**

It is great that you chose to wait for your test results before you had sex.

Do you think you will be able to not have sex until you get the next test results? If you are planning to have sex, do you think you will be able to use a condom during sex?

- What might get in the way of you using a condom?
- How could you plan for that challenge and work around it?
- How would you feel if you could complete this step?

**If the participant had sex but did not use a condom (high risk):**

It is great that you are thinking about safe sex. From what you have told me, there were some situations where you did not use a condom.

- While we are waiting for your next test results, do you think you will be able to use a condom during sex?
  - Would you consider not having sex at all if you don’t want to use a condom?
  - Do you feel comfortable using a condom? How can we help make you more comfortable?
  - What might get in the way of you using a condom?
  - How could you plan for that challenge and work around it?
  - How would you feel if you could complete this step?

**If the participant had sex and used a condom (low risk):**

It is great that you used a condom to practice safe sex. You have helped to protect your partner.

- While we are waiting for your next test results, do you think you will be able to continue to use a condom during sex?
- What might get in the way of you using a condom?
- How could you plan for that challenge and work around it?
- How would you feel if you could complete this step?

Changing behavior takes time and practice. You will have done something good for yourself and your sexual partner.

**Step 2D: “No interpretation” test result**

As you know, the RT-PCR test could not tell if pieces of the Ebola virus in the *[body fluid]* sample that you provided. This is because the quality of the sample made it difficult to see if we can detect pieces of the Ebola virus. We will keep testing you until we get two test results in a row where pieces of the Ebola virus are not detected in your *[body fluid]*. We would like you to continue to participate in this study by coming back in two weeks to give another sample to us. How do you feel about this? Do you have any questions?

*[Pause to give participant chance to discuss their feelings/emotions]*

*[If this is not the participant’s first test result]:*

Since we could not tell if there are pieces of the Ebola virus in your *[body fluid]*, we recommend that you follow the guidance we provided you at your last visit based on your previous test result.

*[Based on the previous test result, please refer to IPC guidance in Appendix 1 and provide appropriate guidance. In addition, please follow the script for Step 2-4 for the appropriate test result section of the script].*

**Step 5: Condom Demonstration (if necessary)**

*[Offer condom demonstration to participant. Please refer to Appendix 3]*

*[Give each participant 35 condoms]*

**Step 6: HIV testing**

As part of this study, we are offering HIV testing as well today. All results are confidential and this testing is completely optional. Would you like to hear more about the HIV test today?

*[If no, tell them the test will be available next time if they change their mind].*

*[If yes, please refer to Appendix 4]:*

**Step 7: Delivery of pregnancy test results (if test performed)**

[*See Appendix 5*]

**Step 8: BP100 distribution (for pregnant and lactating women)**

[*See Appendix 6*]

**Step 9: Referral to Services**

*[If the participant raises any medical concerns during the session, fill in the survivor clinic referral form and alert the receptionist to link the participant to the survivor clinic for further care].*

*[If is necessary, refer the participant to mental health nurse].*

**Step 10: Concluding message:**

Thank you for participating in this study. We are so happy that you recovered from Ebola. Being sick with Ebola must have been very difficult, and we thank you for doing your part to help us better understand the virus. Do you have any questions for us at this time?

SWEAT

# SOP for Ebola Virus Persistence Study - Post-test Counselling

*[Counselors deliver script in native language]*

**Outline:**

1. Greetings

**Detected and Indeterminate Test Result**

1. Follow-up on risk behavior conversation

2A: Detected test result

2B: Indeterminate test result

1. Negotiate sexual risk reduction steps
2. Negotiate risk reduction

**Not detected Test Result**

1. Follow-up on risk behavior conversation

2C. Not Detected test result

1. Negotiate sexual risk reduction steps
2. Negotiate risk reduction

**No Interpretation Test Result**

1. Follow-up on risk behavior conversation

2D. No Interpretation test result

1. Condom distribution (demonstration if necessary)
2. HIV testing
3. Delivery of pregnancy test results (if test performed)
4. BP100 distribution (for pregnant and lactating women)
5. Referral to services
6. Concluding message

**Step 1: Greetings:**

Hello, and thank you for coming to this counseling session.

**Step 2: Follow-up risk behavior conversation**

**Step 2A: Detected Test Result**

As you know, the RT-PCR test detected pieces of the Ebola virus in the sweat sample that you provided.

The pieces of the Ebola virus will one day leave your sweat. We will keep testing you until we get two test results in a row where pieces of the Ebola virus are not detected in your sweat. We would like you to continue to participate in this study by coming back in two weeks to give another sample to us. How do you feel about this? Do you have any questions?

*[Pause to give participant chance to discuss their feelings/emotions]*

Let me explain a little bit more about what your test results mean.

Because we found pieces of the Ebola virus in your sweat, we recommend you follow some basic guidance to lower the risk of passing Ebola to other people until you get two test results in a row where pieces of the Ebola virus are not detected in your sweat.

**Step 2B: Indeterminate test result**

As you know, the RT-PCR test could not tell if the Ebola virus in the sweat sample that you provided. This is because your sample was on the border between detected and not detected. We will keep testing you until we get two test results in a row where pieces of the Ebola virus are not detected in your sweat. We would like you to continue to participate in this study by coming back in two weeks to give another sample to us. How do you feel about this? Do you have any questions?

*[Pause to give participant chance to discuss their feelings/emotions]*

Let me explain a little bit more about what your test results mean.

We recommend you follow some basic guidance to lower the risk of passing Ebola to other people until you get two test results in a row where pieces of the Ebola virus are not detected in your sweat.

*[Please refer to IPC guidance in Appendix 1 and provide guidance to the participant]*

Let’s talk a bit about things you can do to keep you and people you have sex with safe as we wait for your next test results. You do not have to answer any question that you do not want to answer. Everything you tell me today is completely confidential. If you feel uncomfortable at any time, just let me know and we will move on. We are here to support you.

Let us chat a bit about things you can do in your life to reduce the risk of passing Ebola to a partner.

- Are you married, single, divorced, separated? Has anything changed in your relationship since your last visit?
- Have you had sex with anyone since you came in for your last visit? Do you plan on having sex with anyone soon?

**Detected test result**

*[If yes]:*

Thank you for telling me. Because we found pieces of the Ebola virus in your sweat, we strongly recommend that you do not have sex (manual, oral, vaginal, and anal) until you receive two test results in a row where pieces of the Ebola virus are not detected in your sweat. This will make certain that the chance of spreading Ebola will be as low as possible. What do you think about this?

*[If no]*:

Thank you for telling me. Because we found pieces of the Ebola virus in your sweat, we strongly recommend that you do not have sex (manual, oral, vaginal, and anal) until you receive two test results in a row where pieces of the Ebola virus are not detected in your sweat. Not having sex is the safest thing you can do right now.

**Indeterminate test result**

*[If yes]:*

Thank you for telling me. We strongly recommend that you do not have sex (manual, oral, vaginal, and anal) until you receive two test results in a row where pieces of the Ebola virus are not detected in your sweat. This will make certain that the chance of spreading Ebola will be as low as possible. What do you think about this?

*[If no]*:

Thank you for telling me. We strongly recommend that you do not have sex (manual, oral, vaginal, and anal) until you receive two test results in a row where pieces of the Ebola virus are not detected in your sweat. Not having sex is the safest thing you can do right now.

**Step 3: Negotiate Sexual Risk Reduction Steps**

Let’s talk a bit about your life since the last time we talked. I want to remind you that you do not have to answer anything you do not want to answer. Everything you say today will remain confidential.

*[If you had sex]*:

Who did you have sex with?

Do you think you think you will be able to not have sex with this person?

*[For female participants only]:*

- Are you and your partner trying to have a baby?

*[If yes]:*

I understand that it is important to you and your partner to have a baby. However, not having sex is the safest thing for you and your partner right now. Because we detected pieces of the Ebola virus in your sweat, we ask that you abstain from sex until you have two test results in a row where Ebola virus is not detected.

Now I would like to talk a bit about your relationships. Tell me a bit about how things are going with your partner:

- How do you feel about your relationship?
- How do you feel about the way that your partner treats you?

*[Allow the participant ample time to respond]*

- How do you think your partner or other people in your life would react if they knew you received a test result where pieces of the Ebola virus were detected in your sweat?

*[Probe further for possible intimate partner violence (IPV)* ***only if suspected based on responses to the above.*** *Refer to Appendix 7 and 8****].***

**Step 4B: Negotiate Risk Reduction**

**If the participant did not have sex (low risk):**

It is great that you chose to wait for your test results before you had sex.

- Do you think you will be able to not have sex until you get the next test results?
- How could you plan for that challenge and work around it?
- How would you feel if you could complete this step?

**If the participant had sex (high risk):**

From what you have told me, there were some situations where you recently had sex.

- While we are waiting for your next test results, do you think you will be able to abstain from sex?
  - How could you plan for that challenge and work around it?
  - How would you feel if you could complete this step?

Changing behavior takes time and practice. You will have done something good for yourself and for this Ebola fight by trying out this step.

**“Not Detected Test Result**

**Step 2C: “Not detected” test result**

As you know, the RT-PCR test did not detect pieces of the Ebola virus in the sweat sample that you provided. We will keep testing you until we get two test results in a row where pieces of the Ebola virus are not detected in your sweat. We would like you to continue to participate in this study by coming back in two weeks to give another sample to us. How do you feel about this? Do you have any questions?

*[Pause to give participant chance to discuss their feelings/emotions]*

Let’s talk a bit about things you can do to keep you and people around you safe as we wait for your next test results. You do not have to answer any question that you do not want to answer. Everything you tell me today is completely confidential. If you feel uncomfortable at any time, just let me know and we will move on. We are here to support you.

Let us chat a bit about things you can do in your life to avoid HIV, other STIs, and unwanted pregnancy.

- Are you married, single, divorced, separated? Has anything changed in your relationship since your last visit?
- Have you had sex with anyone since you came in for your last visit? Do you plan on having sex with anyone soon?

Thank you for telling me. We strongly recommend that you use a condom if you have sex (manual, oral, vaginal, and anal). This will make certain that the chance of spreading HIV and other STIs will be as low as possible. What do you think about this?

**Step 3: Negotiate Sexual Risk Reduction Steps**

Let’s talk a bit about your life since the last time we talked. I want to remind you that you do not have to answer anything you do not want to answer. Everything you say today will remain confidential.

*[If you had sex]*:

- Who did you have sex with?
- Were you able to use a condom all of the times you had sex? If not, what happened?
- What is your religion? Does your religion allow you to use a condom?
- Tell me about the things that made it more challenging for you to use a condom.
- What was the difference between the times you used a condom and the times you didn’t?
- How did alcohol and other drugs affect your decision to have sex without a condom?
- What was it about some partners that made it more difficult to use a condom?
- Did you have access to condoms?

Now I would like to talk a bit about your relationships. Tell me a bit about how things are going with your partner:

- How do you feel about your relationship?
- How do you feel about the way that your partner treats you?

*[Allow the participant ample time to respond]*

- How do you think your partner or other people in your life would react if you had received a test result where pieces of the Ebola virus were detected in your sweat?

*[Probe further for possible intimate partner violence (IPV)* ***only if suspected based on responses to the above.*** *Refer to Appendix 7 and 8].*

**Step 4: Negotiate Risk Reduction**

**If the participant did not have sex (low risk):**

It is great that you chose to wait for your test results before you had sex.

Do you think you will be able to not have sex until you get the next test results? If you are planning to have sex, do you think you will be able to use a condom during sex?

- What might get in the way of you using a condom?
- How could you plan for that challenge and work around it?
- How would you feel if you could complete this step?

**If the participant had sex but did not use a condom (high risk):**

It is great that you are thinking about safe sex. From what you have told me, there were some situations where you did not use a condom.

- While we are waiting for your next test results, do you think you will be able to use a condom during sex?
  - Would you consider not having sex at all if you don’t want to use a condom?
  - Do you feel comfortable using a condom? How can we help make you more comfortable?
  - What might get in the way of you using a condom?
  - How could you plan for that challenge and work around it?
  - How would you feel if you could complete this step?

**If the participant had sex and used a condom (low risk):**

It is great that you used a condom to practice safe sex. You have helped to protect your partner.

- While we are waiting for your next test results, do you think you will be able to continue to use a condom during sex?
- What might get in the way of you using a condom?
- How could you plan for that challenge and work around it?
- How would you feel if you could complete this step?

Changing behavior takes time and practice. You will have done something good for yourself and your sexual partner.

**Step 2D: “No interpretation” test result**

As you know, the RT-PCR test could not tell if pieces of the Ebola virus in the sweat sample that you provided. This is because the quality of the sample made it difficult to see if we can detect pieces of the Ebola virus. We will keep testing you until we get two test results in a row where pieces of the Ebola virus are not detected in your sweat. We would like you to continue to participate in this study by coming back in two weeks to give another sample to us. How do you feel about this? Do you have any questions?

*[Pause to give participant chance to discuss their feelings/emotions]*

*[If this is not the participant’s first test result]:*

We recommend that you follow the guidance we provided you at your last visit based on your previous test result.

*[Based on the previous test result, please refer to IPC guidance in Appendix 1 and provide appropriate guidance. In addition, please follow the script for Step 2-4 for the appropriate test result section of the script].*

**Step 5: Condom Demonstration (if necessary)**

*[Offer condom demonstration to participant. Please refer to Appendix 3]*

*[Give each participant 35 condoms]*

**Step 6: HIV testing**

As part of this study, we are offering HIV testing as well today. All results are confidential and this testing is completely optional. Would you like to hear more about the HIV test today?

*[If no, tell them the test will be available next time if they change their mind].*

*[If yes, please refer to Appendix 4]:*

**Step 7: Delivery of pregnancy test results (if test performed)**

[*See Appendix 5*]

**Step 8: BP100 distribution (for pregnant and lactating women)**

[*See Appendix 6*]

**Step 9: Referral to Services**

*[If the participant raises any medical concerns during the session, fill in the survivor clinic referral form and alert the receptionist to link the participant to the survivor clinic for further care].*

*[If is necessary, refer the participant to mental health nurse].*

**Step 10: Concluding message:**

Thank you for participating in this study. We are so happy that you recovered from Ebola. Being sick with Ebola must have been very difficult, and we thank you for doing your part to help us better understand the virus. Do you have any questions for us at this time?

SALIVA

# SOP for Ebola Virus Persistence Study - Post-test Counselling

*[Counselors deliver script in native language]*

**Outline:**

1. Greetings

**Detected and Indeterminate Test Result**

1. Follow-up on risk behavior conversation

2A: Detected test result

2B: Indeterminate test result

1. Negotiate sexual risk reduction steps
2. Negotiate risk reduction

**Not detected Test Result**

1. Follow-up on risk behavior conversation

2C. Not Detected test result

1. Negotiate sexual risk reduction steps
2. Negotiate risk reduction

**No Interpretation Test Result**

1. Follow-up on risk behavior conversation

2D. No Interpretation test result

1. Condom distribution (demonstration if necessary)
2. HIV testing
3. Delivery of pregnancy test results (if test performed)
4. BP100 distribution (for pregnant and lactating women)
5. Referral to services
6. Concluding message

**Step 1: Greetings:**

Hello, and thank you for coming to this counseling session.

**Step 2: Follow-up risk behavior conversation**

**Step 2A: Detected Test Result**

As you know, the RT-PCR test detected pieces of the Ebola virus in the saliva sample that you provided.

The pieces of the Ebola virus will one day leave your saliva. We will keep testing you until we get two test results in a row where pieces of the Ebola virus are not detected in your saliva. We would like you to continue to participate in this study by coming back in two weeks to give another sample to us. How do you feel about this? Do you have any questions?

*[Pause to give participant chance to discuss their feelings/emotions]*

Let me explain a little bit more about what your test results mean.

Because we found pieces of the Ebola virus in your saliva, we recommend you follow some basic guidance to lower the risk of passing Ebola to other people until you get two test results in a row where pieces of the Ebola virus are not detected in your saliva.

**Step 2B: Indeterminate test result**

As you know, the RT-PCR test could not tell if the Ebola virus in the saliva sample that you provided. This is because your sample was on the border between detected and not detected. We will keep testing you until we get two test results in a row where pieces of the Ebola virus are not detected in your saliva. We would like you to continue to participate in this study by coming back in two weeks to give another sample to us. How do you feel about this? Do you have any questions?

*[Pause to give participant chance to discuss their feelings/emotions]*

Let me explain a little bit more about what your test results mean.

We recommend you follow some basic guidance to lower the risk of passing Ebola to other people until you get two test results in a row where pieces of the Ebola virus are not detected in your saliva.

*[Please refer to IPC guidance in Appendix 1 and provide guidance to the participant]*

Let’s talk a bit about things you can do to keep you and your partners safe as we wait for your next test results. You do not have to answer any question that you do not want to answer. Everything you tell me today is completely confidential. If you feel uncomfortable at any time, just let me know and we will move on. We are here to support you.

Let us chat a bit about things you can do in your life to reduce the risk of passing Ebola to someone around you.

- Are you married, single, divorced, separated? Has anything changed in your relationship since your last visit?
- Have you had sex with anyone since you came in for your last visit? Do you plan on having sex with anyone soon?
- Have you had kissed anyone since you came in for your last visit? Do you plan on kissing anyone soon?

**Detected Test Result**

*[If yes]:*

Thank you for telling me. Because we found pieces of the Ebola virus in your saliva, we strongly recommend that you do not have oral sex and do not kiss until you receive two test results in a row where pieces of the Ebola virus are not detected in your saliva. If you want to have oral sex, we ask that you or your partner use a male or female condom. This will make certain that the chance of spreading Ebola will be as low as possible. What do you think about this?

*[If no]*:

Thank you for telling me. Because we found pieces of the Ebola virus in your saliva, we strongly recommend that you do not have oral sex and do not kiss until you receive two test results in a row where pieces of the Ebola virus are not detected in your saliva. Not having oral sex and not kissing is the safest thing you can do right now. Do you think it will be possible for you to not have oral sex or use male or female condoms if you do in the future?

**Indeterminate Test Result**

*[If yes]:*

Thank you for telling me. We strongly recommend that you do not have oral sex and do not kiss until you receive two test results in a row where pieces of the Ebola virus are not detected in your saliva. If you want to have oral sex, we ask that you or your partner use a male or female condom. This will make certain that the chance of spreading Ebola will be as low as possible. What do you think about this?

*[If no]*:

Thank you for telling me. We strongly recommend that you do not have oral sex and do not kiss until you receive two test results in a row where pieces of the Ebola virus are not detected in your saliva. Not having oral sex and not kissing is the safest thing you can do right now. Do you think it will be possible for you to not have oral sex or use male or female condoms if you do in the future?

**Step 3: Negotiate Sexual Risk Reduction Steps**

Let’s talk a bit about your life since the last time we talked. I want to remind you that you do not have to answer anything you do not want to answer. Everything you say today will remain confidential.

*[If you had sex]*:

- Who did you have sex with?
- Were you able to use a condom all of the times you had sex? If not, what happened?
- What is your religion? Does your religion allow you to use a condom?
- Tell me about the things that made it more challenging for you to use a condom.
- What was the difference between the times you used a condom and the times you didn’t?
- How did alcohol and other drugs affect your decision to have sex without a condom?
- What was it about some partners that made it more difficult to use a condom?
- Did you have access to condoms?

Now I would like to talk a bit about your relationships. Tell me a bit about how things are going with your partner:

- How do you feel about your relationship?
- How do you feel about the way that your partner treats you?

*[Allow the participant ample time to respond]*

- How do you think your partner or other people in your life would react if they knew you received a test result where pieces of the Ebola virus were detected in your saliva?

*[Probe further for possible intimate partner violence (IPV)* ***only if suspected based on responses to the above.*** *Refer to Appendix 7 and 8].*

**Step 4: Negotiate Risk Reduction**

**If the participant did not have oral sex (low risk):**

It is great that you chose to wait for your test results before you had sex.

Do you think you will be able to not have oral sex until you get the next test results? If you are planning to have oral sex, do you think you will be able to use a condom during sex?

Do you think you can refrain from kissing or exchanging saliva until you receive two consecutive results where pieces of Ebola are not detected in your saliva?

- What might get in the way of you using a condom or preventing the exchange of saliva?
- How could you plan for that challenge and work around it?
- How would you feel if you could complete this step?

**If the participant had oral sex but did not use a condom (high risk):**

It is great that you are thinking about safe oral sex. From what you have told me, there were some situations where you did not use a condom.

While we are waiting for your next test results, do you think you will be able to use a condom during oral sex? Do you think you can refrain from kissing or exchanging saliva until you receive two consecutive results where pieces of Ebola are not detected in your saliva?

- - Would you consider not having oral sex at all if you don’t want to use a condom?
  - Do you feel comfortable using a condom? How can we help make you more comfortable?
  - What might get in the way of you using a condom or preventing the exchange of saliva?
  - How could you plan for that challenge and work around it?
  - How would you feel if you could complete this step?

**If the participant had oral sex and used a condom (low risk):**

It is great that you used a condom to practice safe oral sex. You have helped to protect your partner.

While we are waiting for your next test results, do you think you will be able to continue to use a condom during oral sex? Do you think you can refrain from kissing or exchanging saliva until you receive two consecutive results where pieces of Ebola are not detected in your saliva?

- What might get in the way of you using a condom or preventing the exchange of saliva?
- How could you plan for that challenge and work around it?
- How would you feel if you could complete this step?

Changing behavior takes time and practice. You will have done something good for yourself and for this Ebola fight by trying out this step.

**“Not Detected” Test Result**

**Step 2C: “Not detected” test result**

As you know, the RT-PCR test did not detect pieces of the Ebola virus in the saliva sample that you provided. We will keep testing you until we get two test results in a row where pieces of the Ebola virus are not detected in your saliva. We would like you to continue to participate in this study by coming back in two weeks to give another sample to us. How do you feel about this? Do you have any questions?

*[Pause to give participant chance to discuss their feelings/emotions]*

Let’s talk a bit about things you can do to keep you and people around you safe as we wait for your next test results. You do not have to answer any question that you do not want to answer. Everything you tell me today is completely confidential. If you feel uncomfortable at any time, just let me know and we will move on. We are here to support you.

Let us chat a bit about things you can do in your life to avoid HIV, other STIs, and unwanted pregnancy.

- Are you married, single, divorced, separated? Has anything changed in your relationship since your last visit?
- Have you had sex with anyone since you came in for your last visit? Do you plan on having sex with anyone soon?

Thank you for telling me. We strongly recommend that you use a condom if you have sex (manual, oral, vaginal, and anal). This will make certain that the chance of spreading HIV and other STIs will be as low as possible. What do you think about this?

**Step 3: Negotiate Sexual Risk Reduction Steps**

Let’s talk a bit about your life since the last time we talked. I want to remind you that you do not have to answer anything you do not want to answer. Everything you say today will remain confidential.

*[If you had sex]*:

- Who did you have sex with?
- Were you able to use a condom all of the times you had sex? If not, what happened?
- What is your religion? Does your religion allow you to use a condom?
- Tell me about the things that made it more challenging for you to use a condom.
- What was the difference between the times you used a condom and the times you didn’t?
- How did alcohol and other drugs affect your decision to have sex without a condom?
- What was it about some partners that made it more difficult to use a condom?
- Did you have access to condoms?

Now I would like to talk a bit about your relationships. Tell me a bit about how things are going with your partner:

- How do you feel about your relationship?
- How do you feel about the way that your partner treats you?

*[Allow the participant ample time to respond]*

- How do you think your partner or other people in your life would react if you had received a test result where pieces of the Ebola virus were detected in your saliva?

*[Probe further for possible intimate partner violence (IPV)* ***only if suspected based on responses to the above.*** *Refer to Appendix 7 and 8].*

**Step 4: Negotiate Risk Reduction**

**If the participant did not have sex (low risk):**

It is great that you chose to wait for your test results before you had sex.

Do you think you will be able to not have sex until you get the next test results? If you are planning to have sex, do you think you will be able to use a condom during sex?

- What might get in the way of you using a condom?
- How could you plan for that challenge and work around it?
- How would you feel if you could complete this step?

**If the participant had sex but did not use a condom (high risk):**

It is great that you are thinking about safe sex. From what you have told me, there were some situations where you did not use a condom.

- While we are waiting for your next test results, do you think you will be able to use a condom during sex?
  - Would you consider not having sex at all if you don’t want to use a condom?
  - Do you feel comfortable using a condom? How can we help make you more comfortable?
  - What might get in the way of you using a condom?
  - How could you plan for that challenge and work around it?
  - How would you feel if you could complete this step?

**If the participant had sex and used a condom (low risk):**

It is great that you used a condom to practice safe sex. You have helped to protect your partner.

- While we are waiting for your next test results, do you think you will be able to continue to use a condom during sex?
- What might get in the way of you using a condom?
- How could you plan for that challenge and work around it?
- How would you feel if you could complete this step?

Changing behavior takes time and practice. You will have done something good for yourself and your sexual partner.

**Step 2D: “No interpretation” test result**

As you know, the RT-PCR test could not tell if pieces of the Ebola virus in the saliva sample that you provided. This is because the quality of the sample made it difficult to see if we can detect pieces of the Ebola virus. We will keep testing you until we get two test results in a row where pieces of the Ebola virus are not detected in your saliva. We would like you to continue to participate in this study by coming back in two weeks to give another sample to us. How do you feel about this? Do you have any questions?

*[Pause to give participant chance to discuss their feelings/emotions]*

*[If this is not the participant’s first test result]:*

We recommend that you follow the guidance we provided you at your last visit based on your previous test result.

*[Based on the previous test result, please refer to IPC guidance in Appendix 1 and provide appropriate guidance. In addition, please follow the script for Step 2-4 for the appropriate test result section of the script].*

**Step 5: Condom Demonstration (if necessary)**

*[Offer condom demonstration to participant. Please refer to Appendix 3]*

*[Give each participant 35 condoms]*

**Step 6: HIV testing**

As part of this study, we are offering HIV testing as well today. All results are confidential and this testing is completely optional. Would you like to hear more about the HIV test today?

*[If no, tell them the test will be available next time if they change their mind].*

*[If yes, please refer to Appendix 4]:*

**Step 7: Delivery of pregnancy test results (if test performed)**

[*See Appendix 5*]

**Step 8: BP100 distribution (for pregnant and lactating women)**

[*See Appendix 6*]

**Step 9: Referral to Services**

*[If the participant raises any medical concerns during the session, fill in the survivor clinic referral form and alert the receptionist to link the participant to the survivor clinic for further care].*

*[If is necessary, refer the participant to mental health nurse].*

**Step 10: Concluding message:**

Thank you for participating in this study. We are so happy that you recovered from Ebola. Being sick with Ebola must have been very difficult, and we thank you for doing your part to help us better understand the virus. Do you have any questions for us at this time?

TEARS

# SOP for Ebola Virus Persistence Study - Post-test Counselling

*[Counselors deliver script in native language]*

**Post-Test Outline:**

1. Greetings
2. Follow-up on risk behavior conversation

2A: Detected test result

2B. Indeterminate test result

2C. Not detected test result

2D. No Interpretation test result

1. Negotiate sexual risk reduction steps
2. Negotiate risk reduction
3. Condom distribution (demonstration if necessary)
4. HIV testing
5. Delivery of pregnancy test results (if test performed)
6. BP100 distribution (for pregnant and lactating women)
7. Referral to services
8. Concluding message

**Step 1: Greetings:**

Hello, and thank you for coming to this counseling session.

**Detected Test Result**

**Step 2: Follow-up risk behavior conversation**

**Step 2A: Detected test result**

As you know, the RT-PCR test detected pieces of the Ebola virus in the tears sample that you provided.

The pieces of the Ebola virus will one day leave your tears. We will keep testing you until we get two test results in a row where pieces of the Ebola virus are not detected in your tears. We would like you to continue to participate in this study by coming back in two weeks to give another sample to us. How do you feel about this? Do you have any questions?

*[Pause to give participant chance to discuss their feelings/emotions]*

Let me explain a little bit more about what your test results mean.

Because we found pieces of the Ebola virus in your tears, we recommend you follow some basic guidance to lower the risk of passing Ebola to other people until you get two test results in a row where pieces of the Ebola virus are not detected in your tears.

*[Please refer to IPC guidance in Appendix 1 and provide guidance to the participant]*

**Step 2B: Indeterminate test result**

As you know, the RT-PCR test could not tell if the Ebola virus was in the tears sample that you provided. This is because your sample was on the border between detected and not detected. We will keep testing you until we get two test results in a row where pieces of the Ebola virus are not detected in your tears. We would like you to continue to participate in this study by coming back in two weeks to give another sample to us. How do you feel about this? Do you have any questions?

*[Pause to give participant chance to discuss their feelings/emotions]*

**Step 2C: “Not detected” test result**

As you know, the RT-PCR test did not detect pieces of the Ebola virus in the tears sample that you provided. We will keep testing you until we get two test results in a row where pieces of the Ebola virus are not detected in your tears. We would like you to continue to participate in this study by coming back in two weeks to give another sample to us. How do you feel about this? Do you have any questions?

*[Pause to give participant chance to discuss their feelings/emotions]*

**Step 2D: “No interpretation” test result**

As you know, the RT-PCR test could not tell if pieces of the Ebola virus in the tears sample that you provided. This is because the quality of the sample made it difficult to see if we can detect pieces of the Ebola virus. We will keep testing you until we get two test results in a row where pieces of the Ebola virus are not detected in your tears. We would like you to continue to participate in this study by coming back in two weeks to give another sample to us. How do you feel about this? Do you have any questions?

*[Pause to give participant chance to discuss their feelings/emotions]*

*[If this is not the participant’s first test result]:*

We recommend that you follow the guidance we provided you at your last visit based on your previous test result.

*[Based on the previous test result, please refer to IPC guidance in Appendix 1 and provide appropriate guidance. In addition, please follow the script for Step 2-4 for the appropriate test result section of the script].*

Let’s talk a bit about things you can do to keep you and people around you safe as we wait for your next test results. You do not have to answer any question that you do not want to answer. Everything you tell me today is completely confidential. If you feel uncomfortable at any time, just let me know and we will move on. We are here to support you.

Let us chat a bit about things you can do in your life to avoid HIV, other STIs, and unwanted pregnancy.

- Are you married, single, divorced, separated? Has anything changed in your relationship since your last visit?
- Have you had sex with anyone since you came in for your last visit? Do you plan on having sex with anyone soon?

Thank you for telling me. We strongly recommend that you use a condom if you have sex (manual, oral, vaginal, and anal). This will make certain that the chance of spreading HIV and other STIs will be as low as possible. What do you think about this?

**Step 3: Negotiate Sexual Risk Reduction Steps**

Let’s talk a bit about your life since the last time we talked. I want to remind you that you do not have to answer anything you do not want to answer. Everything you say today will remain confidential.

*[If you had sex]*:

- Who did you have sex with?
- Were you able to use a condom all of the times you had sex? If not, what happened?
- What is your religion? Does your religion allow you to use a condom?
- Tell me about the things that made it more challenging for you to use a condom.
- What was the difference between the times you used a condom and the times you didn’t?
- How did alcohol and other drugs affect your decision to have sex without a condom?
- What was it about some partners that made it more difficult to use a condom?
- Did you have access to condoms?

Now I would like to talk a bit about your relationships. Tell me a bit about how things are going with your partner:

- How do you feel about your relationship?
- How do you feel about the way that your partner treats you?

*[Allow the participant ample time to respond]*

- How do you think your partner or other people in your life would react if they knew you had received a test result where pieces of the Ebola virus were detected in your tears?

*[Probe further for possible intimate partner violence (IPV)* ***only if suspected based on responses to the above.*** *Refer to Appendix 7 and 8].*

**Step 4: Negotiate Risk Reduction**

**If the participant did not have sex (low risk):**

It is great that you chose to wait for your test results before you had sex.

Do you think you will be able to not have sex until you get the next test results? If you are planning to have sex, do you think you will be able to use a condom during sex?

- What might get in the way of you using a condom?
- How could you plan for that challenge and work around it?
- How would you feel if you could complete this step?

**If the participant had sex but did not use a condom (high risk):**

It is great that you are thinking about safe sex. From what you have told me, there were some situations where you did not use a condom.

- While we are waiting for your next test results, do you think you will be able to use a condom during sex?
  - Would you consider not having sex at all if you don’t want to use a condom?
  - Do you feel comfortable using a condom? How can we help make you more comfortable?
  - What might get in the way of you using a condom?
  - How could you plan for that challenge and work around it?
  - How would you feel if you could complete this step?

**If the participant had sex and used a condom (low risk):**

It is great that you used a condom to practice safe sex. You have helped to protect your partner.

- While we are waiting for your next test results, do you think you will be able to continue to use a condom during sex?
- What might get in the way of you using a condom?
- How could you plan for that challenge and work around it?
- How would you feel if you could complete this step?

Changing behavior takes time and practice. You will have done something good for yourself and your sexual partner.

**Step 5: Condom Demonstration (if necessary)**

*[Offer condom demonstration to participant. Please refer to Appendix 3]*

*[Give each participant 35 condoms]*

**Step 6: HIV testing**

As part of this study, we are offering HIV testing as well today. All results are confidential and this testing is completely optional. Would you like to hear more about the HIV test today?

*[If no, tell them the test will be available next time if they change their mind].*

*[If yes, please refer to Appendix 4]:*

**Step 7: Delivery of pregnancy test results (if test performed)**

[*See Appendix 5*]

**Step 8: BP100 distribution (for pregnant and lactating women)**

[*See Appendix 6*]

**Step 9: Referral to Services**

*[If the participant raises any medical concerns during the session, fill in the survivor clinic referral form and alert the receptionist to link the participant to the survivor clinic for further care].*

*[If is necessary, refer the participant to mental health nurse].*

**Step 10: Concluding message:**

Thank you for participating in this study. We are so happy that you recovered from Ebola. Being sick with Ebola must have been very difficult, and we thank you for doing your part to help us better understand the virus. Do you have any questions for us at this time?

BREAST MILK

# SOP for Ebola Virus Persistence Study - Post-test Counselling

*[Counselors deliver script in native language]*

**Post-Test Outline:**

1. Greetings
2. Follow-up on risk behavior conversation

2A: Detected test result

2B. Indeterminate test result

2C. Not detected test result

2D. No Interpretation test result

1. Negotiate sexual risk reduction steps
2. Negotiate risk reduction
3. Condom distribution (demonstration if necessary)
4. HIV testing
5. Delivery of pregnancy test results (if test performed)
6. BP100 distribution (for pregnant and lactating women)
7. Referral to services

Concluding message

**Step 1: Greetings**

Hello, my name is *[name of counselor]*, and I am going to deliver the results of the RT-PCR test we did on the breast milk that you submitted on [*date of sample collection*].

**Step 2: Follow-up on risk behavior conversation**

**Step 2A: Detected Test Result**

As you know, the RT-PCR test detected pieces of the Ebola virus in the breast milk sample that you provided.

The pieces of the Ebola virus will one day leave your breast milk. We will keep testing you until we get two test results in a row where pieces of the Ebola virus are not detected in your breast milk. We would like you to continue to participate in this study by coming back in three days to give another sample to us. How do you feel about this? Do you have any questions?

*[Pause to give participant chance to discuss their feelings/emotions]*

Let me explain a little bit more about what your test results mean.

It is possible that Ebola virus could be passed to others through breastfeeding or sexual activity that involves the touching or kissing of your breasts. To protect your baby, **you should not breastfeed** until you receive two RT-PCR negative test results in a row where pieces of Ebola virus are not detected in your breast milk.

You will receive your test results within 3 days after the sample collection. You will be offered testing every three days until two consecutive RT-PCR tests in a row do not detected pieces of the Ebola virus in your breast milk. If you have a first “not detected” test result, you will come back after 3 days to do a confirmatory test.

Until these two “not detected” tests, I will arrange a visit with you and a Ministry of Health (MOH) nutrition officer who will give you some safe nutrition resources to feed your baby. These include:

- BP100
- Ready to Use Infant Formula
- Ultra-high temperature processing (UHT) milk for children between 6 and 12 months old

This nutrition officer will help you understand this new way of feeding your baby until you receive two RT-PCR “not detected” test results on your breast milk in a row. Her name is *[name]* and her number is *[number]*.

*[The counsellor should now give the ready to use infant formula to the participant and explain to her how to use the formula. Remind her to* ***exclusively*** *use the formula to feed her baby].*

**Table 1. Amount of prepared RUIF an infant needs per day (120–150 ml/kg/day)^[[2]](#footnote-2)^**

| **Age of infant in months** | **Amount of formula per day** | **Approximate # of flasks (200ml*)** | **Number of feeds per day** | **Size of feed in ml** |
| --- | --- | --- | --- | --- |
| 0–1  1–2  2–3  3–4  4–5  5–6 | 450 ml  600 ml  750 ml  750 ml  900 ml  900 ml | 2  3  4  4  5  5 | 8  7  6  6  6  6 | 60ml  90ml  120ml  120ml  150ml  150ml |
| *100 ml contains on average 70 kcal | | | | |

Do you understand how to use this formula? Do you have any questions?

You may experience some pain in your breasts due to suddenly stopping breastfeeding. You can manually express this milk into an absorbent pad like the ones we will give you today or a plastic cup that can be disposed of. Dispose of the milk in a sanitary sewer or pit latrine and burn the pad or cup. Immediately wash the breast and your hands with water and soap. You may also be able to continue producing breast milk and eventually resume nursing your baby once you have had two “not detected” test results. We can provide counselling support to help you continue producing breast milk by expressing it if you would like to continue breastfeeding once the virus pieces are no longer detectable.

Lactating Ebola survivors whose breast milk is “detected” for Ebola virus by RT-PCR should practice good hand and personal hygiene by immediately and thoroughly washing with soap and water after any contact with breast milk. Any other exposed objects or equipment contaminated with breast milk should be washed with water and soap and then decontaminated by soaking them in a 0.5% chlorine solution for about 15 minutes. Linen or clothing contaminated with breast milk should ideally be safely disposed and incinerated; if laundered, linen should be washed with detergent and water first, rinsed and then soaked in 0.5% chlorine solution for approximately 15 minutes. This may cause damage to some fabrics.^[[3]](#footnote-3)^

Do you understand how to express and safely dispose of your breast milk? Do you understand how to practice safe hygiene with your breast milk? Do you have any questions?

*[Please refer to Appendix 1 for appropriate IPC guidance and provide information to the participant]*

**Step 2B: Indeterminate test result**

As you know, the RT-PCR test could not tell if the Ebola virus in the breast milk sample that you provided. This is because your sample was on the border between detected and not detected. We will keep testing you until we get two test results in a row where pieces of the Ebola virus are not detected in your breast milk. We would like you to continue to participate in this study by coming back in two weeks to give another sample to us. How do you feel about this? Do you have any questions?

*[Pause to give participant chance to discuss their feelings/emotions]*

**Step 2C: “Not detected” test result**

As you know, the RT-PCR test did not detect pieces of the Ebola virus in the breast milk sample that you provided. We will keep testing you until we get two test results in a row where pieces of the Ebola virus are not detected in your breast milk. We would like you to continue to participate in this study by coming back in two weeks to give another sample to us. How do you feel about this? Do you have any questions?

*[Pause to give participant chance to discuss their feelings/emotions]*

**Step 2D: “No interpretation” test result**

As you know, the RT-PCR test could not tell if pieces of the Ebola virus in the breast milk sample that you provided. This is because the quality of the sample made it difficult to detect pieces of the Ebola virus. We will keep testing you until we get two test results in a row where pieces of the Ebola virus are not detected in your breast milk. We would like you to continue to participate in this study by coming back in two weeks to give another sample to us. How do you feel about this? Do you have any questions?

*[Pause to give participant chance to discuss their feelings/emotions]*

*[If this is not the participant’s first test result]:*

Since we could not tell if there are pieces of the Ebola virus in your *[body fluid]*, we recommend that you follow the guidance we provided you at your last visit based on your previous test result.

*[Based on the previous test result, please refer to IPC guidance in Appendix 1 and provide appropriate guidance. In addition, please follow the script for Step 2-4 for the appropriate test result section of the script].*

Let’s talk a bit about things you can do to keep you and people around you safe as we wait for your next test results. You do not have to answer any question that you do not want to answer. Everything you tell me today is completely confidential. If you feel uncomfortable at any time, just let me know and we will move on. We are here to support you.

Let us chat a bit about things you can do in your life to avoid HIV, other STIs, and unwanted pregnancy.

- Are you married, single, divorced, separated? Has anything changed in your relationship since your last visit?
- Have you had sex with anyone since you came in for your last visit? Do you plan on having sex with anyone soon?

Thank you for telling me. We strongly recommend that you use a condom if you have sex (manual, oral, vaginal, and anal). This will make certain that the chance of spreading HIV and other STIs will be as low as possible. What do you think about this?

**Step 3: Negotiate Sexual Risk Reduction Steps**

Let’s talk a bit about your life since the last time we talked. I want to remind you that you do not have to answer anything you do not want to answer. Everything you say today will remain confidential.

*[If you had sex]*:

- Who did you have sex with?
- Were you able to use a condom all of the times you had sex? If not, what happened?
- What is your religion? Does your religion allow you to use a condom?
- Tell me about the things that made it more challenging for you to use a condom.
- What was the difference between the times you used a condom and the times you didn’t?
- How did alcohol and other drugs affect your decision to have sex without a condom?
- What was it about some partners that made it more difficult to use a condom?
- Did you have access to condoms?

Now I would like to talk a bit about your relationships. Tell me a bit about how things are going with your partner:

- How do you feel about your relationship?
- How do you feel about the way that your partner treats you?

*[Allow the participant ample time to respond]*

- How do you think your partner or other people in your life would react if they knew you had received a test result where pieces of the Ebola virus were detected in your *breast milk*?

*[Probe further for possible intimate partner violence (IPV)* ***only if suspected based on responses to the above.*** *Refer to Appendix 7 and 8].*

**Step 4: Negotiate Risk Reduction**

**If the participant did not have sex (low risk):**

It is great that you chose to wait for your test results before you had sex.

Do you think you will be able to not have sex until you get the next test results? If you are planning to have sex, do you think you will be able to use a condom during sex?

- What might get in the way of you using a condom?
- How could you plan for that challenge and work around it?
- How would you feel if you could complete this step?

**If the participant had sex but did not use a condom (high risk):**

It is great that you are thinking about safe sex. From what you have told me, there were some situations where you did not use a condom.

- While we are waiting for your next test results, do you think you will be able to use a condom during sex?
  - Would you consider not having sex at all if you don’t want to use a condom?
  - Do you feel comfortable using a condom? How can we help make you more comfortable?
  - What might get in the way of you using a condom?
  - How could you plan for that challenge and work around it?
  - How would you feel if you could complete this step?

**If the participant had sex and used a condom (low risk):**

It is great that you used a condom to practice safe sex. You have helped to protect your partner.

- While we are waiting for your next test results, do you think you will be able to continue to use a condom during sex?
- What might get in the way of you using a condom?
- How could you plan for that challenge and work around it?
- How would you feel if you could complete this step?

Changing behavior takes time and practice. You will have done something good for yourself and your sexual partner.

**Step 5: Condom Demonstration (if necessary)**

*[Offer condom demonstration to participant. Please refer to Appendix 3]*

*[Give each participant 35 condoms]*

**Step 6: HIV testing**

As part of this study, we are offering HIV testing as well today. All results are confidential and this testing is completely optional. Would you like to hear more about the HIV test today?

*[If no, tell them the test will be available next time if they change their mind].*

*[If yes, please refer to Appendix 4]:*

**Step 7: Delivery of pregnancy test results (if test performed)**

[*See Appendix 5*]

**Step 8: BP100 distribution (for pregnant and lactating women)**

[*See Appendix 6*]

**Step 9: Referral to Services**

*[If the participant raises any medical concerns during the session, fill in the survivor clinic referral form and alert the receptionist to link the participant to the survivor clinic for further care].*

*[If is necessary, refer the participant to mental health nurse].*

**Step 10: Concluding message:**

Thank you for participating in this study. We are so happy that you recovered from Ebola. Being sick with Ebola must have been very difficult, and we thank you for doing your part to help us better understand the virus. Do you have any questions for us at this time?

Appendix 1: IPC Guidance

|  |  |  |  |  |  |  |  |  |  |
| --- | --- | --- | --- | --- | --- | --- | --- | --- | --- |
|  | **Semen** | **Vaginal fluid** | **Menstrual blood** | **Urine** | **Rectal fluid** | **Sweat** | **Tears** | **Saliva** | **Breast milk** |
| Timing of guidance delivery |  |  |  |  |  |  |  |  |  |
| - EBOV persistence pre-test counseling | ✓ |  |  |  |  |  |  |  |  |
| - EBOV persistence post-test counselling | ✓ | ✓ | ✓ | ✓ | ✓ | ✓ | ✓ | ✓ | ✓ |
| Sexual risk reduction guidance |  |  |  |  |  |  |  |  |  |
| - Abstain from manual, oral, vaginal or anal sex. If you have sex, use a male or female condom. | ✓ | ✓ | ✓ | ✓ | ✓ |  |  |  |  |
| - Abstain only |  |  |  |  |  | ✓ |  |  |  |
| - Abstain from kissing and oral sex only |  |  |  |  |  |  |  | ✓ |  |
| - Avoid kissing and touching of breasts during sex |  |  |  |  |  |  |  | ✓ | ✓ |
| Sexual risk reduction guidance if sexual activity occurs |  |  |  |  |  |  |  |  |  |
| - After sex, masturbation, or any genital contact, wash and dry or thoroughly and wipe genital and anal area with a clean towel or tissue but do not use the same towel or tissue on both areas | ✓ | ✓ | ✓ | ✓ | ✓ |  |  |  |  |
| - Safely throw away tissues with body fluids, and/or used condom somewhere where other people cannot touch it, like a covered bin, pit latrine, or burn pit. For towels, wash in hot water before reusing | ✓ | ✓ | ✓ | ✓ | ✓ |  |  |  |  |
| - After sex or any genital contact, wash hands thoroughly with soap and water | ✓ | ✓ | ✓ | ✓ | ✓ |  |  |  |  |
| - No sexual risk reduction guidance |  |  |  |  |  |  | ✓ |  |  |
| General infection prevention and control (IPC) guidance |  |  |  |  |  |  |  |  |  |
| - Wash hands often with soap and water | ✓ | ✓ | ✓ | ✓ | ✓ | ✓ | ✓ | ✓ | ✓ |
| - Try not to touch your eyes, nose, and mouth with unwashed hands. | ✓ | ✓ | ✓ | ✓ | ✓ | ✓ | ✓ | ✓ | ✓ |
| - After urinating or defecating, wash hands well with soap and water. | ✓ | ✓ | ✓ | ✓ | ✓ |  |  |  |  |
| - Cover your mouth and nose when sneezing or coughing. |  |  |  |  |  |  |  | ✓ |  |
| - Do not share personal items such as razors, toothbrushes or eating utensils with others |  |  |  |  |  | ✓ |  | ✓ |  |
| - Use clean tissues or towels to wipe body, hands, mouth, eyes |  |  |  |  |  | ✓ | ✓ | ✓ | ✓ |
| Breastfeeding guidance |  |  |  |  |  |  |  |  |  |
| - Do not breastfeed until pieces of the Ebola virus are not detected in two consecutive specimens |  |  |  |  |  |  |  |  | ✓ |

Appendix 2: Mango Tree Analogy for qRT-PCR Results

This is a picture of a mango tree. The tree has many parts: the leaves, the mangos and the bark. These are pieces which come together and make the mango tree. In the same way, different pieces of the virus together make up the whole virus. The RT-PCR test tries to detect pieces of the Ebola virus and not the whole virus. If we detect a piece of the Ebola virus, we do not know if the virus is alive or not. In the same way, if you find a mango and leaves on the street, you do not know if there is a mango tree that is alive. You only found pieces of the mango tree.

- Do you understand what we mean when we say that the RT-PCR test detects pieces of the Ebola virus?


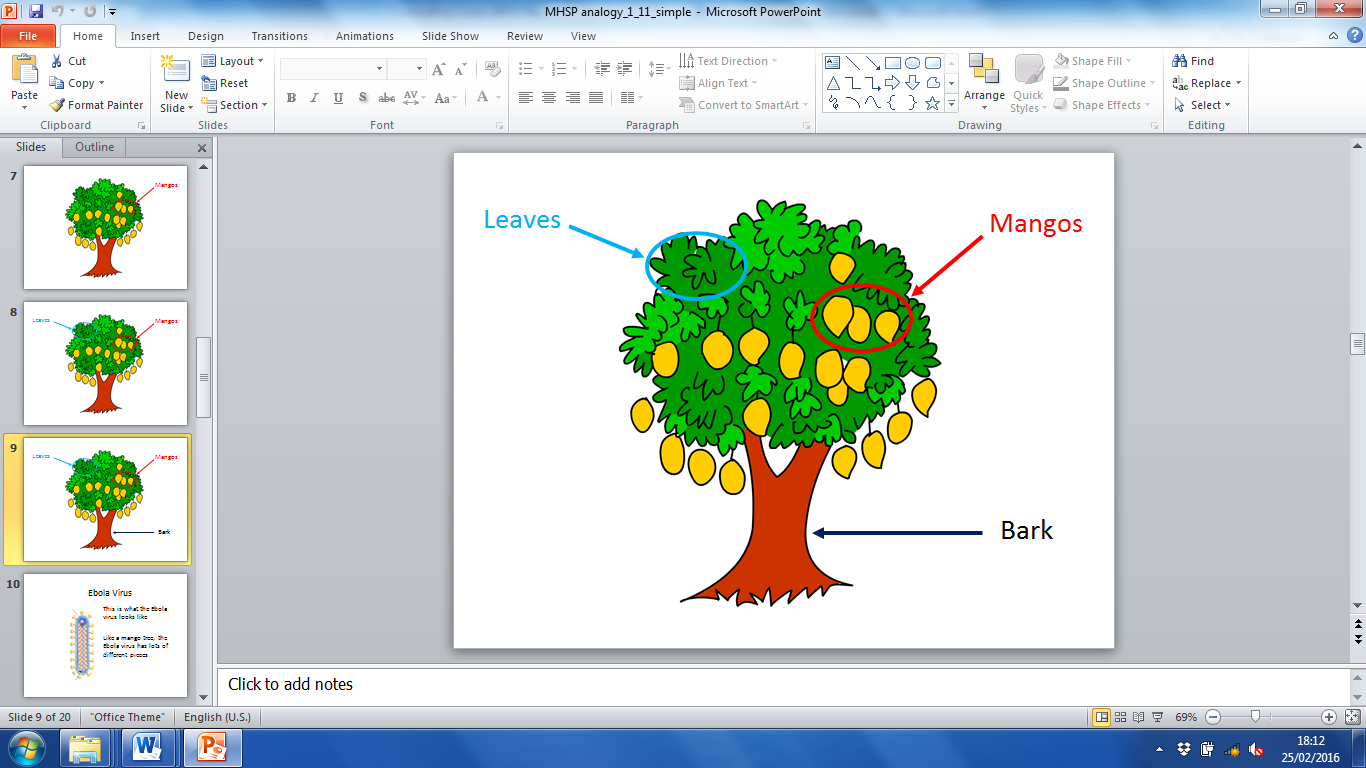


Appendix 3: Condom Demonstration

MALE CONDOM

Now, I will show you how to properly use a male condom. Before opening the condom, you must:

- Check the expiry date of the condom. Do not use an expired condom.
- Press gently on the corners of the condom package and check that the condom moves. If the condom does not move, do not use the condom.

In order to open the condom:

- Do not use your teeth or mouth.
- Look for the edges of the condom that are jagged and rip along this edge.

Then:

- Slide the condom out of the package.
- Place the condom on the erect penis with the tip sticking up.
- Pinch the tip of the condom and roll the side of the condom down with your fingers.

Once the man has ejaculated in the condom:

- While the penis is erect, carefully roll up the condom so that the semen does not leak out of the condom.
- Tie the condom in a knot so that the semen does not leak out.
- Put the condom in a tissue and throw the tissue in a place where other people cannot come into contact with it.
- Male condoms are not reusable- use a new one every time you have sex.

FEMALE CONDOM

Now, I will show you how to properly use a female condom. Before opening the condom, you must:

- Check the expiry date of the condom. Do not use an expired condom.
- Press gently on the corners of the condom package and check that the condom moves. If the condom does not move, do not use the condom.

In order to open the condom:

- Do not use your teeth or mouth.
- Look for the edges of the condom that are jagged and rip along this edge.

Then:

- Slide the condom out of the package.
- Relax and get into a comfortable position.
- Squeeze together the sides of the inner ring at the closed end of the condom and slide it into your vagina. Push the inner ring into your vagina as far as it can go, up to your cervix. Make sure it’s not twisted
- Pull out your finger and let the outer ring hang about an inch outside the vagina
- Guide your partner’s penis into the opening of the condom, making sure it doesn’t slip to the side between the condom and your vaginal walls
- To use the female condom for anal sex, remove the inner ring and insert the condom into your anus with your finger leaving the outer ring hanging out

Once the man has ejaculated in the condom:

- After sex, twist the outer ring to keep semen inside the pouch
- Gently pull it out of your vagina or anus, being careful not to spill any semen
- Put the condom in a tissue and throw the tissue in a place where other people cannot come into contact with it
- Female condoms are not reusable- use a new one every time you have sex

Appendix 4: HIV Testing^1^

# HIV Pre-test Counseling for EVD Survivors

[*If the participant would like to hear more about the HIV test*]

- Have you heard of HIV before?
- Do you know how someone can get HIV?
- Do you know how to prevent yourself and others from getting HIV?
- Do you know that having sex without condoms can also put you at risk for HIV infection, other STIs, and unwanted pregnancy?

[*Correct any misunderstandings about HIV*]

The test offered today is in line with Sierra Leone’s national HIV testing program. It is a rapid test, which means that we will get the initial results in 15 minutes today. All results are confidential and if you test positive for HIV, we will refer you to a doctor. Medication to treat HIV is free in Sierra Leone. This test is completely optional. You do not have to do the test if you do not want to. Not taking the test will not affect your participation in the study.

- Would you like to take the HIV test today?

[*If yes, perform the HIV test. Use Determine. If not, use Insta*]:

[*If participant is not interested in taking the HIV test*]:

- Why do you not want to take the HIV test?

[*Tell the participant that the HIV test will be available at their next visit, if they change their mind*]

# HIV Post-test Counseling for EVD Survivors

Post-test counselling is provided for all people whether they test HIV positive or negative. The aims of post-test counselling are to give the test result to the client, provide emotional support to help the person cope with the result, discuss the physical, emotional and social implications of a positive result, discuss prevention for HIV-positive and HIV-negative individuals, and refer the client for any care or treatment indicated.

**Readiness for results**

Prior to giving the test results, the counsellor should ensure that the client is truly willing and ready to receive their results, and understands what both positive and negative test results mean.

**Giving test results**

The counsellor should give the test results calmly, in a quiet, private setting. The results should be available to the client the same day, and every effort should be made to reduce the waiting time for the client. Every opportunity should be given to allow the client to express their feelings about the test results and any other issue concerning the client. There should be ample time for the client to ask questions about the meaning of the test results and any other issues. One-to-one or couple counselling can be used to give results, depending on the clients’ preference. Clients may specifically request that a family member, friend, or other supportive person be in the room when they receive results, though the counsellor should make sure that this is truly desired by the client.

**Risk Reduction Planning**

Every post-test counselling session should include the development of a risk reduction plan specific to the client’s test results and personal life situation. The counsellor should help the client understand the importance of avoiding future risky exposure to HIV.

**Window period**

There is a time called the **‘window period’** which is from the time of getting infected to the time when the body has produced enough antibodies *to be detected on an HIV antibody test*. This period is usually about three months. This means that a person who has just been infected may test negative for HIV because their body has not produced enough antibodies to be picked up by the test. Such a person *can still pass the virus to others*, if for instance he or she donates blood, has unprotected sex or to her baby if she becomes pregnant.

Clients who test negative but who may have been exposed to HIV through risk-taking behaviour should be encouraged to return for a repeat test in 6-12 weeks. Clients who test negative with no possibility of recent exposure (in the previous 6-12 weeks) to HIV do not need to be encouraged to come for an additional test. Clients who may be in the window period should be encouraged to practice risk reduction behaviours during this period. However, HIV negative clients with no recent possible exposure to HIV do not need to be told to come for confirmatory testing.

**Family Planning, counselling and education**

Information on family planning, its role for both HIV+ and HIV- clients, and how to access services should be included in HCT counselling sessions. If possible, FP services should be provided at HCT sites. If the client is negative, during post-test counselling the counsellor should encourage good reproductive health seeking behaviour. The counsellor should reinforce the client’s use of FP, if already using a method, and additionally counsel for condom use, if the client is at risk of HIV and AIDS. If the HIV negative client is not planning to become pregnant and is not using a method, referral to an appropriate FP service delivery point should be made.

If the client is HIV positive (and male/female) the risks of pregnancy should be clearly explained, and the client made aware of risks to herself and to the unborn child if a pregnancy is carried to term. The counsellor should know where to refer the client for a full range of FP methods, including long-term or permanent methods. It is important to ensure that the client understands the choices available and can make her own decision.

**Condom education and distribution**

Condom education, demonstration, and distribution should be part of every post-test counselling session, and all clients, both HIV+ and HIV-, should be given condoms during the post-test session. The dual protection against HIV and against unwanted pregnancies should be emphasized. However, clients who refuse condoms should not be coerced to receive them.

**Additional counselling sessions**

HCT clients, both HIV+ and HIV-, should be encouraged to return for additional counselling and prevention education. It should be recognized that many HCT clients need time to absorb their results, and additional counselling sessions may be beneficial for all clients. HCT centres should have an “open door” policy for their clients for ongoing supportive counselling. Such additional counselling should deal with both health and non-health issues, including legal and workplace problems the client may have encountered.

**Partner Notification**

The counsellor should encourage the client to bring in their partner(s) for couple counselling and testing.

**Positive Living**

All HIV+ clients should be counselled about “living positively with HIV” which includes maintaining a positive attitude, avoiding additional exposure to the virus and other STIs, adhere to their medication, good nutrition, join PLHIV support and other social support groups.

**Referrals**

The counsellor should make appropriate referrals to additional services as needed, such as medical, social, legal, spiritual, and psychological support if the counsellor determines that these services would be helpful. Especially for clients who are HIV+, post-test support services should include treatment services for TB, opportunistic infections, and other sexually transmitted diseases.

**Participant is HIV-:**

1. Review some of the issues covered in pre-test session, e.g. what HIV positive and negative mean, how the person anticipated coping with results and check the person wishes to receive the result

2. Give the result simply and clearly

3. Give time to let the person consider results

4. Let the person talk about his/her feelings about the results, making sure that the person considers the implications of the result

5. Check with open-ended questions that the person understands the meaning of the result:

S/he is not infected with HIV (majority of people) **or**

S/he is in the window period (a very small minority)

6. Discuss any other immediate concerns the person might raise

7. Discuss the importance of staying negative; provision of condoms and demonstration

8. Make a risk reduction plan, e.g. safer sex, sharing result with partner, partner attending VCCT,

modifying use of drugs and alcohol, health-seeking behavior such as screening for STI

9. Inform person that counselling is available in the future and that s/he may come alone or with a partner, close friend or relative

**Participant is HIV+:**

*1.* Review some of the issues covered in pre-test session, e.g. what HIV positive and negative mean, how the person anticipated coping with results

2. Give the result simply and clearly

3. Give time to let the person consider results

4. Let the person talk about his/her feelings about the results

5. Check with open-ended questions that the person understands the meaning of the result

Refer to difference between HIV and AIDS

Give clear, factual explanations

Acknowledge the shock of the diagnosis; offer support

Encourage hope

6. Discuss benefits of knowing one’s status re PMTCT, IPT, ARVs, social and emotional support services

7. Discuss how the person is going to get through the next hours and days

8. Check to see who is available to give immediate support

9. Discuss immediate concerns the person might raise

10. Discuss prevention of further transmission; provision and demonstration of condoms

11. Arrange to see the person again soon

- When counselling the person with a positive test result, handle the situation very sensitively. Remember that the person is likely very anxious and that the purpose of reviewing pre-test is to prepare the person to receive the result and to remind him/her of some of the coping mechanisms s/he may have thought of to cope with the result. This is not meant to be a quiz to see how much the person remembers.

**Indeterminate Results:**

In the case of indeterminate results, counsellors must not give a result, even if under pressure from the client, explaining the reasons for the need to repeat the test.

*^1^HIV Testing SOPS provided by Joint Programme on HIV/AIDS, Freetown, Sierra Leone*

Appendix 5: Delivery of Pregnancy Test Results

We had performed the pregnancy test in the sample collection tent for you today. I am here now to deliver the results. Remember that this information is confidential and that the study team is here to support you.

[*If the participant is has a positive pregnancy test result*]:

Your pregnancy test shows that you are pregnant.

[*Pause to give the participant some time*].

- How do you feel about this test result?

I want to inform you that there is no evidence to show that women who survive Ebola and then become pregnant can pass Ebola to their baby.

It is very important for all pregnant women to receive antenatal care (ANC) to keep the woman and the baby safe. Are you currently attending ANC?

- - [*If yes*]*:*
    - *Document which ANC the participant is attending by writing it in the ANC log book.*
    - *Inform the operations manager.*
  - [*If no*]*:*
    - We can help you find ANC in your area. We can refer you to the doctor in the survivor clinic today so that they can help find you ANC.
    - Would you like to do that?
    - *Refer the participant to the doctor for referral to ANC.*

[*If the participant expresses the desire to terminate the pregnancy*]:

From what I understand, you would like to terminate your pregnancy. Today, we can refer you to the doctor who can refer you to the appropriate services to terminate your pregnancy?

- Would you like to be referred to the doctor to discuss this with him/her?

Appendix 6: BP100 Distribution for Pregnant and Lactation Women

When you are a pregnant or lactating woman, your body works extra hard so it is important to make sure you are healthy and take good care of yourself. I would like to provide you with some nutritious biscuit called BP100 which will help keep you healthy.

These biscuit can be eaten as biscuits or you can add warm water to the biscuit and eat it as porridge. If you make porridge, it should be eaten within 2 hours. We recommend that you eat a biscuit three times a day. When you eat these biscuits, make sure that you drink plenty of water. If you have difficulty drinking water, do not eat BP100.

Today I will give you 6 boxes of BP100. Each box has 9 biscuits. This supply is enough for 2 weeks. At your next appointment in two weeks, I will give you another 2 week supply of BP100 biscuits. When you are discharged from the study, you are always welcome to come back to the study site every two week to get your supply of BP100, as long as you are pregnant or breastfeeding your baby.

I will also give you a guidance sheet that has more information about the BP100 biscuits.

- Do you have any questions about the BP100 biscuits?

**BP100 Guidance Sheet**

Appendix 7: Intimate Partner Violence (IPV) Script

I’d like to hear a bit more about what you’re telling me about how your partner treats you. These questions are personal, so please remember that this session is completely confidential and we do not have to talk about anything you do not want to talk about.

- - Do you feel controlled or isolated by your partner?
  - Does your partner ever hit, kick, hurt, or threaten you?
  - Every couple rights sometimes. What are your fights like at home? Do your fights ever get physical?
  - Are you ever afraid of your partner? Do you ever feel that you are in danger?
  - Does your partner refuse to practice safe sex?
  - Do you feel you have control over whether you or your partner uses a condom?
  - How would your partner react if you asked him to use a condom? How would you feel if he asked you to use a condom?

*[If a participant indicates prior violence or concern for future violence then we will do the following]:*

Women who disclose any form of violence by an intimate partner (or other family member) or sexual assault by any perpetrator should be offered immediate support. A health-care providers should, as a minimum, offer first-line support when women disclose violence. First-line support includes:

- Being non-judgemental and supportive and validating what the woman is saying
- Providing practical care and support that responds to her concerns, but does not intrude by asking about her history of violence
- Listening carefully, but not pressuring her to talk (care should be taken when discussing sensitive topics when interpreters are involved)
- Helping her access information about resources, including legal and other services that she might think helpful, assisting her to increase safety for herself and her children, where needed
- Providing or mobilizing social support. Providers should ensure: that the consultation is conducted in private, confidentiality^[[4]](#footnote-4)^

Connect the participant to services available in the community in accordance with the Sierra Leone National Referral Protocol (see Appendix 1 for referral pathway). As much as possible should be done during first contact, in case the woman does not return. Follow-up support, care, and the negotiation of safe and accessible means for follow-up consultation should be offered*.^[[5]](#footnote-5)^*

*[The following should be communicated to the participant]:*

It sounds like you are concerned about some aspects of your relationship. We would like to connect you with resources to help you cope with this and to feel safe. To start, we would like the hospital’s psychiatric nurse who has been trained in these issues to meet with you. Do you feel comfortable doing that? Would you like to speak to him/her? We can arrange for you to speak to him/her as soon as possible. We also encourage you to report this information to the Family Support Unit at the local Police Station. We can direct you to the Station. How do you feel about that?”

*[Offer again that the participant can halt participation in the study, or if they wish to continue, work to set a plan for how they can keep themselves safe].*

Appendix 8: Domestic Violence Referral Pathways

Sierra Leone National Referral Protocol on Gender Based Violence

**In doing referral, the importance of minimizing further trauma to the survivors must be emphasized, and the efficiency of professionals who are in place to support them must be maximized.**

9.1 The referral pathway for a child or woman victim of Gender Based Violence and other forms of abuse is noted in diagram form, and attached as Appendix 1. The diagram denotes the referral pathways to be followed at the first point of contact i.e. when the child or woman victim of abuse first presents for help and/or to report an incident of abuse.

9.2 The first point of contact is defined as the person, in either a professional capacity or other position of responsibility/authority (Chief, Religious Leader, Elder etc) to whom the victim discloses details of the abuse.

9.3 A factual account of the abuse, including details of the victim, perpetrator, and what actually happened (e.g. the victim was sexually assaulted by the perpetrator) should be provided in written form by the referring agency to the Sierra Leone Police Family Support Unit, and the Ministry of Social Welfare, Gender and Children’s Affairs.

9.4 If the victim requires urgent medical attention, he or she should in the first instance be referred for medical treatment to the local MoHS service provider (e.g. government medical facilities). Once this has been done the case should be reported to the Family Support Unit at the first available opportunity to ensure prompt investigation of the alleged abuse.

9.5 It is important to note that there is more than one possible referral route when the victim/survivor is first reporting the incident/seeking help. For example, although the incident needs to be reported to the Sierra Leone Police/Family Support Unit as quickly as possible, the victim may require immediate medical attention/treatment.

9.6 Professionals should exercise a ‘common sense approach’ in this regard –for instance if the victim has an open wound, or is in considerable pain and distress, medical care should be the first priority.

9.7 If there is suspicion of, or evidence suggesting victim trafficking, response services should be coordinated via the MSWGCA Trafficking in Persons Focal Point, who will in turn liaise with the International Organisation for Migration. Details will also need to be referred to the Sierra Leone Police Family Support Unit for prompt investigation.

**Referral Path Ways for women and child victims of Sexual and Domestic Violence**

Rape/Unlawful Canal Knowledge and Serious Physical Wounding with Intent or Torture

**C**

**B**

**A**

**Hospital / Health centre**

**Court**

**Police**

**FSU**

**Survivor/ Victim**

**D**

**Protective Care**

Home/community/other

**E**

**Social Services**

NGOs, Line Ministries, local councils, CWCs

| **Legend** | |
| --- | --- |
| A | - Medical examination and report - Medical treatment |
| B | - Investigation - Prosecution - Counselling |
| C | Trial- Punishment of perpetrator   - Protection of victim/survivor - Compensation of victim/survivor |
| D | Protective Care   - Free of attack, discrimination and stigmatization |
| E | - Counselling - Education/skills training/gainful employment - Shelter, medical & other needs - Legal counsel |

**Other Gender based violence that does not involve Physical Intrusion or Wounding**

**e.g. forced/child marriage, fondling, indecent assault, child labor, child trafficking**

**C**

**B**

**A**

**CWC**

**Court**

**Police**

**FSU**

**Survivor/ Victim**

**D**

**Protective Care**

Home/community/other

**E**

**Social Services**

NGOs, Line Ministries, local councils

| **Legend** | |
| --- | --- |
| **A** | - Social Investigation - Mediation - Counselling - Referral |
| **B** | - Investigation - Prosecution - Counselling |

Appendix 9: Delivery of Virus Isolation Results

1. Preparation
2. Greetings
3. Delivery of results
   1. Detected test results
      1. Participant still not discharged from the study
      2. Participant has been discharged from the study
   2. Not detected test results
      1. Participant still not discharged from the study
      2. Participant has been discharged from the study
4. Participant concerned with test results
5. **Preparation**

Research assistant will provide the counselor with the following information:

1. Virus isolation:
   1. Test result
      1. Detected means that the virus was grown
      2. Not detected means that the virus not grown
   2. Date that the specimen was initially tested
   3. Date of shipment of specimen to USA for testing
2. Record of current RT-PCR test results to help customize the counseling messages for the participant

Depending on the virus isolation result and the most recent RT-PCR results, the counselor will choose from the following counseling messages.

1. **Greetings**

Hello, my name is [*counselor’s name*] and I am going to deliver the results of the virus isolation test we did on the [*body fluid*] that you provided on [*date of sample collection*].

First, let me explain about the virus isolation test. The virus isolation test is a test that we can do to see if pieces of the Ebola virus that were detected in your semen on [*date of sample collection*] was alive or dead. Remember that the RT-PCR test cannot tell if the virus is alive or dead, only if pieces of the virus are there. Only the virus isolation test can tell us if the virus is alive or dead.

To do this test, we sent your detected [*body fluid*] samples to Atlanta, USA on *[date sample was sent].* There, we did the virus isolation test that tries to grow Ebola in a laboratory using the [*body fluid*] sample that you provided.

1. **Delivery of Results**

**a. Detected test results**

Using the virus isolation test, we were able to grow Ebola from the sample that you provided on [*date of sample collection*]. This means that the virus we found when we tested your semen was alive.

1. **Participant still not discharged from the study**

It is possible that you may be able to pass Ebola to a person who has contact with your semen through activity like manual, oral, vaginal, or anal sex. We recommend that you to continue to use condoms or abstain from sex and we would like to keep testing your [*body fluid*] samples until you have two test results in a row where pieces of the Ebola virus cannot be detected in your [*body fluid*].

- How do you feel about your virus isolation test results?
- Do you think that you can continue not having sex or use a condom every time you have sex?
- Do you have any questions or concerns?

Thank you for continuing your participation. Your participation is helping us understand more about the Ebola virus and will help Ebola survivors in the future.

1. **Participant has been discharged from the study**

Since the time you gave us this sample where virus was detected, you have received two test results where pieces of the Ebola virus were not detected in your semen. We had told you when you received these test results that you did not need to continue using condoms or abstinence to prevent passing Ebola to a sex partner, and this is still true. However, we encourage you to continue to use condoms to protect yourself from HIV, other STIs and unwanted pregnancy.

Thank you for continuing your participation. Your participation is helping us understand more about the Ebola virus and will help Ebola survivors in the future.

**b. Detected test results**

Using the virus isolation test, we did not grow Ebola from the sample that you provided on [*date of sample collection*]. This means that the pieces of the Ebola virus we found when we tested your [*body fluid*] were dead. Let me explain a little bit more about what your test results mean.

1. **Participant still not discharged from the study**

It is possible that even though we did not grow the virus at that time, it could still be alive in your body and able to infect others. So, we recommend that you to continue to use condoms or abstain from manual, oral, vaginal, or anal sex. We would like to keep testing your samples until you have two test results in a row where pieces of the Ebola virus cannot be detected in your [*body fluid*].

- How do you feel about your virus isolation test results?
- Do you think that you can continue not having sex or using a condom every time you have sex?
- Do you have any questions or concerns?

Thank you for continuing your participation. Your participation is helping us understand more about the Ebola virus and will help Ebola survivors in the future.

1. **Participant has been discharged from the study**

The virus isolation results showed that the virus pieces we detected in your semen on [*date of sample collection*] were dead.

Since the time you gave us this sample, you have received two test results where pieces of the Ebola virus were not detected in your semen. We told you when you received these test results that you did not need to continue using condoms or abstinence to prevent passing Ebola to a sexual partner, and this is still true. However we encourage you to continue to use condoms to protect yourself from HIV, other STIs and unwanted pregnancy.

Thank you for participating in this study.

1. **Participant concerned with test results**

**If the participant has concerns:**

- Counselor decides whether mental health referral is needed (see: SOP for Medical/Mental Health Referrals).
- If referral is refused, ask: Can I follow up with you in a few days to see how you are doing?
- Record contact information and call them within three days for wellness check-up.
- **Wellness Check-up Phone Call Script (to be made within three days of receiving a positive test result if the participant has withdrawn from the study):**
  - How are you feeling? How are you feeling about the virus isolation test results you received?
  - How are you feeling about providing an additional sample for us to submit for RT-PCR testing so we can make sure that pieces of the Ebola virus are not in your semen?
  - We have asked two people who have survived Ebola themselves (1 male, 1 male/female) to talk to participants. Would you like to talk to them about how you are feeling? Can I give them your contact information?

Appendix 10: Talking Points for Male Participants

**Pre-Test**

**Semen**

- Greet the participant:
  - Remember to **express empathy** and happiness that they survived Ebola.
  - Ask how they are doing since they have recovered and if they have any questions.
- Discuss the **purpose** of the visit:
- Discuss the **purpose** of the visit
  - Give information to [the client] about the testing we will do to detect if pieces of the Ebola virus are in their semen.
  - You will also talk with them about things you can do in your life to reduce the risk of passing Ebola to a person you have sex with.
  - Inform the participant that until he receives two “not detected” test results in a row, that he should abstain from sex or use condoms to prevent the possible transmission of Ebola to sexual partners
- Talk about **confidentiality** and **possible discomfort:**
  - Let the participant know that anything they tell you will be confidential.
  - Let the client know that if they feel uncomfortable at any time, they can tell you and you will move on.
  - Inform the client that receiving test results is optional and the choice is entirely up to them.
  - Ask if they would like you to help them strategize how to tell their partner or family members about their test results.
- Tell the survivor it is important that he **monitor his health:**
  - It may be possible that virus could still be present in the body but not able to be found in body fluids.
  - There have been a very small number of survivors who became seriously ill after they recovered, and so we would like to ensure that you are well even after finishing the study.
  - If you feel unwell contact *[doctor 1]* or *[doctor 2]*, the medical doctors at the survivor clinic.
- Talk with client about their **relationships** and their **sexual** **behavior**, **condom** **use**, **condom** **negotiation**, **abstinence**. Give client **condom** **demonstration** and **condoms**.
- **Offer** HIV test
- **Refer** participants with medical or psychological problems to *[doctor 1]* or *[doctor 2]*

**Other Fluids: Rectal, Sweat, Tears, Urine, Saliva**

- Greet the participant:
  - Remember to **express empathy** and happiness that they survived Ebola.
  - Ask how they are doing since they have recovered and if they have any questions.
- Discuss the **purpose** of the visit:
  - Give information to [the client] about the testing we will do to detect if pieces of the Ebola virus are in their fluids.
- Tell participants that at pre-test they do not need to do anything differently right now. If the test does detect pieces of Ebola in any bodily fluid, you will give them more information when you go over the test result with them.
- Talk about **confidentiality** and **possible discomfort:**
  - Let the participant know that anything they tell you will be confidential.
  - Let the client know that if they feel uncomfortable at any time, they can tell you and you will move on.
  - Inform the client that receiving test results is optional and the choice is entirely up to them.
  - Ask if they would like you to help them strategize how to tell their partner or family members about their test results.
- Tell the survivor it is important that he **monitor his health:**
  - Inform participants that it may be possible that virus could still be present in the body but not able to be found in body fluids.
  - There have been a small number of survivors who became seriously ill after they recovered, and so we may ask to follow up with the participant after the study ends to ensure they are well even after finishing the study.
  - If you feel unwell contact *[doctor 1]* or *[doctor 2]*, the medical doctors at the survivor clinic.
- Inform participant that condoms can help prevent against HIV, STIs and unwanted pregnancy. Give client **condom** **demonstration** and **condoms** and discuss **condom negotiation**.
- **Offer** HIV test
- **Refer** participants with medical or psychological problems to *[doctor 1]* or *[doctor 2]*

**Delivery of Results**

**Semen**

**Detected test result:**

- Explain the results:
  - Explain that the RT-PCR test **detected** pieces of the Ebola virus in the sample that they provided.
  - Explain that because of these results, it is possible that they may be able to pass Ebola to a person who has contact with their semen through activity like vaginal, anal, oral, or manual sex.
- Tell the survivor it is important that they **monitor their health:**
  - Inform participants that it may be possible that virus could still be present in the body but not able to be found in body fluids.
  - There have been a small number of survivors who became seriously ill after they recovered, and so we may ask to follow up with the participant after the study ends to ensure they are well even after finishing the study.
  - If you feel unwell contact *[doctor 1]* or *[doctor 2]*, the medical doctors at the survivor clinic.
- Encourage participant to **continue** with the study until they get two “not detected” test results in a row.

**Indeterminate test result:**

- Explain the results:
  - Explain that the RT-PCR test **could not tell** if pieces of the Ebola virus are in the sample that they provided because it was on the border of detected and not detected.
  - Explain that because of these results, it is possible that they may be able to pass Ebola to a person who has contact with their semen through activity like vaginal, anal, oral, or manual sex.
- Tell the survivor it is important that they **monitor their health:**
  - Inform participants that it may be possible that virus could still be present in the body but not able to be found in body fluids.
  - There have been a small number of survivors who became seriously ill after they recovered, and so we may ask to follow up with the participant after the study ends to ensure they are well even after finishing the study.
  - If you feel unwell contact *[doctor 1]* or *[doctor 2]*, the medical doctors at the survivor clinic.
- Encourage participant to **continue** with the study until they get two “not detected” test results in a row.

**1^st^ “Not detected” test result:**

- Explain the results:
  - Explain that the RT-PCR test **did not detect** pieces of the Ebola virus in the sample that they provided.
  - Explain that we would like to test them again to make sure pieces of Ebola are not detected in their fluids.
  - Explain that although no pieces of Ebola were detected, it is important that the participant continues to abstain from sex or use a condom
- Tell the survivor it is important that they **monitor their health:**
  - Inform participants that it may be possible that virus could still be present in the body but not able to be found in body fluids.
  - There have been a small number of survivors who became seriously ill after they recovered, and so we may ask to follow up with the participant after the study ends to ensure they are well even after finishing the study.
  - If you feel unwell contact *[doctor 1]* or *[doctor 2]*, the medical doctors at the survivor clinic.
- Encourage participant to **continue** with the study until they get two “not detected” test results in a row.

**“No interpretation” test result:**

- Explain the results:
  - Explain that the RT-PCR test **could not tell** if pieces of the Ebola virus are in the sample that they provided because the quality made it difficult to see if there were pieces of the Ebola virus.
  - Explain that it is important that the participant continues to abstain from sex or use a condom in order to prevent possible Ebola transmission.
- Tell the survivor it is important that they **monitor their health:**
  - Inform participants that it may be possible that virus could still be present in the body but not able to be found in body fluids.
  - There have been a small number of survivors who became seriously ill after they recovered, and so we may ask to follow up with the participant after the study ends to ensure they are well even after finishing the study.
  - If you feel unwell contact *[doctor 1]* or *[doctor 2]*, the medical doctors at the survivor clinic.
- Encourage participant to **continue** with the study until they get two “not detected” test results in a row.

**2^nd^** **“Not Detected” test result:**

- **Discharge** participant from the study.

**Delivery of Results**

**Urine, Rectal fluid, Sweat, Tears, Saliva**

**Detected test result:**

- Explain the results:
  - Explain that the RT-PCR test **detected** pieces of the Ebola virus in the sample that they provided.

*[For urine, rectal fluid, sweat]:*

- - Explain that because of these results, it is possible that they may be able to pass Ebola to a person who has contact with their *[body fluid]* through activity like vaginal, anal, oral, or manual sex.

*[For saliva]:*

- - Explain that because of these results, it is possible that they may be able to pass Ebola to a person who has contact with their *[body fluid]* through activity like oral sex and kissing

*[For tears]:*

- - Explain that because of these results, it is possible that they may be able to pass Ebola to a person who has contact with their *[body fluid]*.
- Tell the survivor it is important that they **monitor their health:**
  - Inform participants that it may be possible that virus could still be present in the body but not able to be found in body fluids.
  - There have been a small number of survivors who became seriously ill after they recovered, and so we may ask to follow up with the participant after the study ends to ensure they are well even after finishing the study.
  - If you feel unwell contact *[doctor 1]* or *[doctor 2]*, the medical doctors at the survivor clinic.
- Encourage participant to **continue** with the study until they get two “not detected” test results in a row.

**Indeterminate test result:**

- Explain the results:
  - Explain that the RT-PCR test **could not tell** if pieces of the Ebola virus are in the sample that they provided because it was on the border of detected and not detected.

*[For urine, rectal fluid, sweat]:*

- - Explain that because of these results, it is possible that they may be able to pass Ebola to a person who has contact with their *[body fluid]* through activity like vaginal, anal, oral, or manual sex.

*[For saliva]:*

- - Explain that because of these results, it is possible that they may be able to pass Ebola to a person who has contact with their *[body fluid]* through activity like oral sex and kissing

*[For tears]:*

- - Explain that because of these results, it is possible that they may be able to pass Ebola to a person who has contact with their *[body fluid]*.
- Tell the survivor it is important that they **monitor their health:**
  - Inform participants that it may be possible that virus could still be present in the body but not able to be found in body fluids.
  - There have been a small number of survivors who became seriously ill after they recovered, and so we may ask to follow up with the participant after the study ends to ensure they are well even after finishing the study.
  - If you feel unwell contact *[doctor 1]* or *[doctor 2]*, the medical doctors at the survivor clinic.
- Encourage participant to **continue** with the study until they get two “not detected” test results in a row.

**1^st^ “Not detected” test result:**

- Explain the results:
  - Explain that the RT-PCR test **did not detect** pieces of the Ebola virus in the sample that they provided.
  - Explain that we would like to test them again to make sure pieces of Ebola are not detected in their fluids.
- Tell the survivor it is important that they **monitor their health:**
  - Inform participants that it may be possible that virus could still be present in the body but not able to be found in body fluids.
  - There have been a small number of survivors who became seriously ill after they recovered, and so we may ask to follow up with the participant after the study ends to ensure they are well even after finishing the study.
  - If you feel unwell contact *[doctor 1]* or *[doctor 2]*, the medical doctors at the survivor clinic.
- Encourage participant to **continue** with the study until they get two “not detected” test results in a row.

**“No Interpretation” test result**

- Explain the results:
  - Explain that the RT-PCR test **could not tell** if pieces of the Ebola virus are in the sample that they provided because the quality made it difficult to see if there were pieces of the Ebola virus.
- Tell the survivor it is important that they **monitor their health:**
  - Inform participants that it may be possible that virus could still be present in the body but not able to be found in body fluids.
  - There have been a small number of survivors who became seriously ill after they recovered, and so we may ask to follow up with the participant after the study ends to ensure they are well even after finishing the study.
  - If you feel unwell contact *[doctor 1]* or *[doctor 2]*, the medical doctors at the survivor clinic.
- Encourage participant to **continue** with the study until they get two “not detected” test results in a row.

**2^nd^ “Not Detected” test result:**

- **Discharge** participant from the study.

**Post-Test Counseling**

**Semen**

**Positive/Indeterminate/Not detected/No interpretation test result:**

- **Explain** results again, **ask** if participant has any questions.
- Explain to participants that there are basic good health habits they will need to follow to make sure the risk of transmitting Ebola through their *[body fluid]* is as low as possible until they get two “not detected” RT-PCR results in a row.
- *[Refer to IPC guidance in Appendix 1 of the counselling script]*
- **Explain** to participants that they may be able to pass Ebola to a person who has contact with *[body fluid]* through activity like vaginal, anal, oral, or manual sex until they get two “not detected” test results in a row.
- Tell the survivor it is important that they **monitor their health:**
  - Inform participants that it may be possible that virus could still be present in the body but not able to be found in body fluids.
  - There have been a small number of survivors who became seriously ill after they recovered, and so we may ask to follow up with the participant after the study ends to ensure they are well even after finishing the study.
  - If you feel unwell contact *[doctor 1]* or *[doctor 2]*, the medical doctors at the survivor clinic.
- Talk with client about their **relationships** and their **sexual** **behavior**, **condom** **use**, **condom** **negotiation**, **abstinence**.
- Give client **condom** **demonstration** and **condoms**.
- **Refer** participants with medical or psychological problems to *[doctor 1]* or *[doctor 2]*.
- **Encourage** client to continue until they get two “not detected” RT-PCR test results in a row.

**Post-Test Counseling**

**Urine, Rectal fluid**

**Positive/Indeterminate/Not detected/No interpretation test result:**

- **Explain** results again, **ask** if participant has any questions.

*[For detected or indeterminate test result only]:*

- Explain to participants that there are basic good health habits they will need to follow to make sure the risk of transmitting Ebola through their *[body fluid]* is as low as possible until they get two “not detected” RT-PCR results in a row.
- *[Refer to IPC guidance in Appendix 1 of the counselling script]*

*[For detected or indeterminate test result only]:*

- **Explain** to participants that they may be able to pass Ebola to a person who has contact with *[body fluid]* through activity like vaginal, anal, oral, or manual sex until they get two “not detected” test results in a row.
- Tell the survivor it is important that they **monitor their health:**
  - Inform participants that it may be possible that virus could still be present in the body but not able to be found in body fluids.
  - There have been a small number of survivors who became seriously ill after they recovered, and so we may ask to follow up with the participant after the study ends to ensure they are well even after finishing the study.
  - If you feel unwell contact *[doctor 1]* or *[doctor 2]*, the medical doctors at the survivor clinic.
- Talk with client about their **relationships** and their **sexual** **behavior**, **condom** **use**, **condom** **negotiation**, **abstinence**.
- Give client **condom** **demonstration** and **condoms**.
- **Refer** participants with medical or psychological problems to *[doctor 1]* or *[doctor 2]*.
- **Encourage** client to continue until they get two “not detected” RT-PCR test results in a row.

**Post-Test Counseling**

**Sweat**

**Positive/Indeterminate/Not detected/No interpretation test result:**

- **Explain** results again, **ask** if participant has any questions.

*[For detected or indeterminate test result only]:*

- Explain to participants that there are basic good health habits they will need to follow to make sure the risk of transmitting Ebola through their *[body fluid]* is as low as possible until they get two “not detected” RT-PCR results in a row.
- *[Refer to IPC guidance in Appendix 1 of the counselling script]*

*[For detected or indeterminate test result only]:*

- **Explain** to participants that they may be able to pass Ebola to a person who has contact with *[body fluid]* through activity like vaginal, anal, oral, or manual sex until they get two “not detected” test results in a row.
- Tell the survivor it is important that they **monitor their health:**
  - Inform participants that it may be possible that virus could still be present in the body but not able to be found in body fluids.
  - There have been a small number of survivors who became seriously ill after they recovered, and so we may ask to follow up with the participant after the study ends to ensure they are well even after finishing the study.
  - If you feel unwell contact *[doctor 1]* or *[doctor 2]*, the medical doctors at the survivor clinic.
- Talk with client about their **relationships** and their **sexual** **behavior**, **condom** **use**, **condom** **negotiation**, **abstinence**.
- Give client **condom** **demonstration** and **condoms**.
- **Refer** participants with medical or psychological problems to *[doctor 1]* or *[doctor 2]*.
- **Encourage** client to continue until they get two “not detected” RT-PCR test results in a row.

**Post-Test Counseling**

**Saliva**

**Positive/Indeterminate/Not detected/No interpretation test result:**

- **Explain** results again, **ask** if participant has any questions.

*[For detected or indeterminate test result only]:*

- Explain to participants that there are basic good health habits they will need to follow to make sure the risk of transmitting Ebola through their *[body fluid]* is as low as possible until they get two “not detected” RT-PCR results in a row.
- *[Refer to IPC guidance in Appendix 1 of the counselling script]*

*[For detected or indeterminate test result only]:*

- **Explain** to participants that they may be able to pass Ebola to a person who has contact with *[body fluid]* through activity like oral sex and kissing until they get two “not detected” test results in a row.
- Tell the survivor it is important that they **monitor their health:**
  - Inform participants that it may be possible that virus could still be present in the body but not able to be found in body fluids.
  - There have been a small number of survivors who became seriously ill after they recovered, and so we may ask to follow up with the participant after the study ends to ensure they are well even after finishing the study.
  - If you feel unwell contact *[doctor 1]* or *[doctor 2]*, the medical doctors at the survivor clinic.
- Talk with client about their **relationships** and their **sexual** **behavior**, **condom** **use**, **condom** **negotiation**, **abstinence**.
- Give client **condom** **demonstration** and **condoms**.
- **Refer** participants with medical or psychological problems to *[doctor 1]* or *[doctor 2]*.
- **Encourage** client to continue until they get two “not detected” RT-PCR test results in a row.

**Post-Test Counseling**

**Tears**

**Positive/Indeterminate/Not detected/No interpretation test result:**

- **Explain** results again, **ask** if participant has any questions.

*[For detected or indeterminate test result only]:*

- Explain to participants that there are basic good health habits they will need to follow to make sure the risk of transmitting Ebola through their *[body fluid]* is as low as possible until they get two “not detected” RT-PCR results in a row.
- *[Refer to IPC guidance in Appendix 1 of the counselling script]*

*[For detected or indeterminate test result only]:*

- **Explain** to participants that they may be able to pass Ebola to a person who has contact with *[body fluid]*.
- Tell the survivor it is important that they **monitor their health:**
  - Inform participants that it may be possible that virus could still be present in the body but not able to be found in body fluids.
  - There have been a small number of survivors who became seriously ill after they recovered, and so we may ask to follow up with the participant after the study ends to ensure they are well even after finishing the study.
  - If you feel unwell contact *[doctor 1]* or *[doctor 2]*, the medical doctors at the survivor clinic.
- Talk with client about their **relationships** and their **sexual** **behavior**, **condom** **use**, **condom** **negotiation**, **abstinence**.
- Give client **condom** **demonstration** and **condoms**.
- **Refer** participants with medical or psychological problems to *[doctor 1]* or *[doctor 2]*.
- **Encourage** client to continue until they get two “not detected” RT-PCR test results in a row.

Appendix 11: Talking Points for Female Participants

**Pre-Test**

**Vaginal fluid, Menstrual Blood, Breast Milk, Rectal fluid, Saliva, Sweat, Tears, Urine**

- Greet the participant:
  - Remember to **express empathy** and happiness that they survived Ebola.
  - Ask how they are doing since they have recovered and if they have any questions.
- Discuss the **purpose** of the visit:
  - Give information to the client about the testing we will do to detect if pieces of the Ebola virus are in their body fluids.
- Tell participants that at pre-test they do not need to do anything differently. If the test does detect pieces of Ebola in any body fluid, you will give them more information when you go over the test result with them at their next visit.
- Talk about **confidentiality** and **possible discomfort:**
  - Let the participant know that anything they tell you will be confidential.
  - Let the client know that if they feel uncomfortable at any time, they can tell you and you will move on.
  - Inform the client that receiving test results is optional and the choice is entirely up to them.
  - Ask if they would like you to help them strategize how to tell their partner or family members about their test results.
- Tell the survivor it is important that they **monitor their health:**
  - Inform participants that it may be possible that virus could still be present in the body but not able to be found in body fluids.
  - There have been a small number of survivors who became seriously ill after they recovered, and so we may ask to follow up with the participant after the study ends to ensure they are well even after finishing the study.
  - If you feel unwell contact *[doctor 1]* or *[doctor 2]*, the medical doctors at the survivor clinic.
- Inform participant that condoms can help prevent against HIV, STIs and unwanted pregnancy. Give client **condom** **demonstration** and **condoms** and discuss **condom negotiation**.
- **Offer** HIV test
- **Refer** participants who report psychological, sexual, or physical harm by their partners **(intimate partner violence)** to proper services.
- **Refer** participants with medical or psychological problems to *[doctor 1]* or *[doctor 2]*

**For Pregnant Participants only (at Pre-Test Visit)**

- Confirm that participant is currently pregnant.
- Give participant information about Ebola and pregnancy:
  - There is no evidence to show that women who survive Ebola and then become pregnant can pose a risk for Ebola virus transmission
  - It is very important for all pregnant women to receive antenatal care (ANC) to keep the participant and her baby safe.
- Ask participant if she is attending/receiving antenatal care:
  - If Yes
    - *Document which ANC she already attends by writing a note-to-file*
    - *Inform receptionist*
  - If No
    - We can help you find antenatal care in your area. You can speak to the nurses and doctors in the survivor clinic today to be connected to antenatal care. Would you like to do that?
    - *Refer to survivor clinic for subsequent referral to appropriate ANC.*
- Inform the participant that maintaining good health is important in pregnancy.
  - Your body works extra hard when you are pregnant so it is important to take good care of yourself. Part of this is a healthy diet. I would like to provide you with some nutritious biscuits which will help keep you healthy.
    - *Distribute nutritional supplement bars and refer to guidance sheet to give instructions.*

**Delivery of Results**

**Vaginal fluid, Menstrual blood, Breast Milk, Rectal, Sweat, Tears, Saliva, Urine**

**Detected test result:**

- Explain the results:
  - Explain that the RT-PCR test **detected** pieces of the Ebola virus in the sample that they provided.

*[For urine, rectal fluid, sweat]:*

- - Explain that because of these results, it is possible that they may be able to pass Ebola to a person who has contact with their *[body fluid]* through activity like vaginal, anal, oral, or manual sex.

*[For saliva]:*

- - Explain that because of these results, it is possible that they may be able to pass Ebola to a person who has contact with their *[body fluid]* through activity like oral sex and kissing

*[For tears]:*

- - Explain that because of these results, it is possible that they may be able to pass Ebola to a person who has contact with their *[body fluid]*.
- Tell the survivor it is important that they **monitor their health:**
  - Inform participants that it may be possible that virus could still be present in the body but not able to be found in body fluids.
  - There have been a small number of survivors who became seriously ill after they recovered, and so we may ask to follow up with the participant after the study ends to ensure they are well even after finishing the study.
  - If you feel unwell contact *[doctor 1]* or *[doctor 2]*, the medical doctors at the survivor clinic.
- Encourage participant to **continue** with the study until they get two “not detected” test results in a row.

**Indeterminate test result:**

- Explain the results:
  - Explain that the RT-PCR test **could not tell** if pieces of the Ebola virus are in the sample that they provided because it was on the border of detected and not detected.

*[For urine, rectal fluid, sweat]:*

- - Explain that because of these results, it is possible that they may be able to pass Ebola to a person who has contact with their *[body fluid]* through activity like vaginal, anal, oral, or manual sex.

*[For saliva]:*

- - Explain that because of these results, it is possible that they may be able to pass Ebola to a person who has contact with their *[body fluid]* through activity like oral sex and kissing

*[For tears]:*

- - Explain that because of these results, it is possible that they may be able to pass Ebola to a person who has contact with their *[body fluid]*.
- Tell the survivor it is important that they **monitor their health:**
  - Inform participants that it may be possible that virus could still be present in the body but not able to be found in body fluids.
  - There have been a small number of survivors who became seriously ill after they recovered, and so we may ask to follow up with the participant after the study ends to ensure they are well even after finishing the study.
  - If you feel unwell contact *[doctor 1]* or *[doctor 2]*, the medical doctors at the survivor clinic.
- Encourage participant to **continue** with the study until they get two “not detected” test results in a row.

**1^st^ “Not detected” test result:**

- Explain the results:
  - Explain that the RT-PCR test **did not detect** pieces of the Ebola virus in the sample that they provided.
  - Explain that we would like to test them again to make sure pieces of Ebola are not detected in their fluids.
- Tell the survivor it is important that they **monitor their health:**
  - Inform participants that it may be possible that virus could still be present in the body but not able to be found in body fluids.
  - There have been a small number of survivors who became seriously ill after they recovered, and so we may ask to follow up with the participant after the study ends to ensure they are well even after finishing the study.
  - If you feel unwell contact *[doctor 1]* or *[doctor 2]*, the medical doctors at the survivor clinic.
- Encourage participant to **continue** with the study until they get two “not detected” test results in a row.

**“No Interpretation” test result**

- Explain the results:
  - Explain that the RT-PCR test **could not tell** if pieces of the Ebola virus are in the sample that they provided because the quality made it difficult to see if there were pieces of the Ebola virus.
- Tell the survivor it is important that they **monitor their health:**
  - Inform participants that it may be possible that virus could still be present in the body but not able to be found in body fluids.
  - There have been a small number of survivors who became seriously ill after they recovered, and so we may ask to follow up with the participant after the study ends to ensure they are well even after finishing the study.
  - If you feel unwell contact *[doctor 1]* or *[doctor 2]*, the medical doctors at the survivor clinic.
- Encourage participant to **continue** with the study until they get two “not detected” test results in a row.

**2^nd^ “Not Detected” test result:**

- **Discharge** participant from the study.

**Post-Test Counseling**

**Vaginal fluid, Menstrual blood, Rectal fluid, Urine**

**Positive/Indeterminate/Not detected/No interpretation test result:**

- **Explain** RT-PCR results again, **ask** if participant has any questions.

*[For detected or indeterminate test result only]:*

- Explain to participants that there are basic good health habits they will need to follow to make sure the risk of transmitting Ebola through their *[body fluid]* is as low as possible until they get two “not detected” RT-PCR results in a row.
- *[Refer to IPC guidance in Appendix 1 of the counselling script]*

*[For detected or indeterminate test result only]:*

- **Explain** to participants that they may be able to pass Ebola to a person who has contact with *[body fluid]* through activity like vaginal, anal, oral, or manual sex until they get two “not detected” test results in a row.
- Tell the survivor it is important that they **monitor their health:**
  - Inform participants that it may be possible that virus could still be present in the body but not able to be found in body fluids.
  - There have been a small number of survivors who became seriously ill after they recovered, and so we may ask to follow up with the participant after the study ends to ensure they are well even after finishing the study.
  - If you feel unwell contact *[doctor 1]* or *[doctor 2]*, the medical doctors at the survivor clinic.
- Talk with client about their **relationships** and their **sexual** **behavior**, **condom** **use**, **condom** **negotiation**, **abstinence**. Give client **condom** **demonstration** and **condoms**.
- **Refer** participants who report psychological, sexual, or physical harm by their partners **(intimate partner violence)** to proper services.
- **Refer** participants with medical or psychological problems to *[doctor 1]* or *[doctor 2]*.
- **Encourage** client to continue until they get two “not detected” RT-PCR test results in a row.

**Post-Test Counseling**

**Sweat**

**Positive/Indeterminate/Not detected/No interpretation test result:**

- **Explain** RT-PCR results again, **ask** if participant has any questions.

*[For detected or indeterminate test result only]:*

- Explain to participants that there are basic good health habits they will need to follow to make sure the risk of transmitting Ebola through their *[body fluid]* is as low as possible until they get two “not detected” RT-PCR results in a row.
- *[Refer to IPC guidance in Appendix 1 of the counselling script]*

*[For detected or indeterminate test result only]:*

- **Explain** to participants that they may be able to pass Ebola to a person who has contact with *[body fluid]* through activity like vaginal, anal, oral, or manual sex until they get two “not detected” test results in a row.
- Tell the survivor it is important that they **monitor their health:**
  - Inform participants that it may be possible that virus could still be present in the body but not able to be found in body fluids.
  - There have been a small number of survivors who became seriously ill after they recovered, and so we may ask to follow up with the participant after the study ends to ensure they are well even after finishing the study.
  - If you feel unwell contact *[doctor 1]* or *[doctor 2]*, the medical doctors at the survivor clinic.
- Talk with client about their **relationships** and their **sexual** **behavior**, **condom** **use**, **condom** **negotiation**, **abstinence**. Give client **condom** **demonstration** and **condoms**.
- **Refer** participants who report psychological, sexual, or physical harm by their partners **(intimate partner violence)** to proper services.
- **Refer** participants with medical or psychological problems to *[doctor 1]* or *[doctor 2]*.
- **Encourage** client to continue until they get two “not detected” RT-PCR test results in a row.

**Post-Test Counseling**

**Saliva**

**Positive/Indeterminate/Not detected/No interpretation test result:**

- **Explain** RT-PCR results again, **ask** if participant has any questions.

*[For detected or indeterminate test result only]:*

- Explain to participants that there are basic good health habits they will need to follow to make sure the risk of transmitting Ebola through their *[body fluid]* is as low as possible until they get two “not detected” RT-PCR results in a row.
- *[Refer to IPC guidance in Appendix 1 of the counselling script]*

*[For detected or indeterminate test result only]:*

- **Explain** to participants that they may be able to pass Ebola to a person who has contact with *[body fluid]* through activity like oral sex and kissing until they get two “not detected” test results in a row.
- Tell the survivor it is important that they **monitor their health:**
  - Inform participants that it may be possible that virus could still be present in the body but not able to be found in body fluids.
  - There have been a small number of survivors who became seriously ill after they recovered, and so we may ask to follow up with the participant after the study ends to ensure they are well even after finishing the study.
  - If you feel unwell contact *[doctor 1]* or *[doctor 2]*, the medical doctors at the survivor clinic.
- Talk with client about their **relationships** and their **sexual** **behavior**, **condom** **use**, **condom** **negotiation**, **abstinence**. Give client **condom** **demonstration** and **condoms**.
- **Refer** participants who report psychological, sexual, or physical harm by their partners **(intimate partner violence)** to proper services.
- **Refer** participants with medical or psychological problems to *[doctor 1]* or *[doctor 2]*.
- **Encourage** client to continue until they get two “not detected” RT-PCR test results in a row.

**Post-Test Counseling**

**Tears**

**Positive/Indeterminate/Not detected/No interpretation test result:**

- **Explain** RT-PCR results again, **ask** if participant has any questions.

*[For detected or indeterminate test result only]:*

- Explain to participants that there are basic good health habits they will need to follow to make sure the risk of transmitting Ebola through their *[body fluid]* is as low as possible until they get two “not detected” RT-PCR results in a row.
- *[Refer to IPC guidance in Appendix 1 of the counselling script]*

*[For detected or indeterminate test result only]:*

- **Explain** to participants that they may be able to pass Ebola to a person who has contact with *[body fluid]*.
- Tell the survivor it is important that they **monitor their health:**
  - Inform participants that it may be possible that virus could still be present in the body but not able to be found in body fluids.
  - There have been a small number of survivors who became seriously ill after they recovered, and so we may ask to follow up with the participant after the study ends to ensure they are well even after finishing the study.
  - If you feel unwell contact *[doctor 1]* or *[doctor 2]*, the medical doctors at the survivor clinic.
- Talk with client about their **relationships** and their **sexual** **behavior**, **condom** **use**, **condom** **negotiation**, **abstinence**. Give client **condom** **demonstration** and **condoms**.
- **Refer** participants who report psychological, sexual, or physical harm by their partners **(intimate partner violence)** to proper services.
- **Refer** participants with medical or psychological problems to *[doctor 1]* or *[doctor 2]*.
- **Encourage** client to continue until they get two “not detected” RT-PCR test results in a row.

**Post-Test Counseling**

**Breast milk**

**Positive/Indeterminate/Not detected/No interpretation test result:**

- **Explain** RT-PCR results again, **ask** if participant has any questions.

*[For detected or indeterminate test result only]:*

- Explain to participants that there are basic good health habits they will need to follow to make sure the risk of transmitting Ebola through their *[body fluid]* is as low as possible until they get two “not detected” RT-PCR results in a row.
- *[Refer to IPC guidance in Appendix 1 of the counselling script]*

*[For detected or indeterminate test result only]:*

- **Explain** to participants that they may be able to pass Ebola to a person who has contact with *[body fluid]*.
  - During the post-test counseling session with a lactating woman with a RT-PCR “detected” or indeterminate test result ask:
    - Are you currently breastfeeding any baby or child?
    - **If yes**, ask for each baby or child: **What is the age of the baby or child? Is the baby or child an Ebola survivor? Is the child healthy or feeling unwell.**
    - It may be possible to pass Ebola virus through your breast milk to your baby. To protect your baby, please follow the guidance below until you receive at least two RT-PCR test results in a row where pieces of Ebola are not detected in your breast milk:
    - If you are breastfeeding **any baby**:
      - Do not breastfeed until you receive two RT-PCR test results in a row where pieces of Ebola are not detected in your breast milk
      - The study staff will contact the nutrition officer for further assistance with alternative infant feeding support for the study participant and her baby.
      - You may experience some pain in your breasts due to suddenly stopping breastfeeding. We will teach you safe ways to express and dispose of this milk to alleviate this pain and reduce inflammation.
        - Our recommended method is that she express into a plastic cup or disposable absorbent pad, pour the liquid in a latrine or toilet, and burn the cup or pad.
      - Use a condom if you engage in manual, oral, vaginal, or anal sex or choose abstinence.
      - Lactating women will receive their results within 3 days after the collection of the specimen. Women with positive breast milk results will be offered testing every 3 days until 2 consecutive RT-PCR tests are negative. Women with a first negative test will come back after 3 days to do a confirmatory test.
    - You will arrange a visit the participant and an MOH nutrition officer who will give her some safe nutrition resources for her baby. Resources that we will give you include:
      - BP100
      - Ready to Use Infant Formula
      - Ultra-high temperature processing (UHT) milk for children between 6 and 12 months old
    - This nutrition specialist will help the participant understand this new way of feeding her baby until she gets a negative test result for her breast milk.
- Tell the survivor it is important that they **monitor their health:**
  - Inform participants that it may be possible that virus could still be present in the body but not able to be found in body fluids.
  - There have been a small number of survivors who became seriously ill after they recovered, and so we may ask to follow up with the participant after the study ends to ensure they are well even after finishing the study.
  - If you feel unwell contact *[doctor 1]* or *[doctor 2]*, the medical doctors at the survivor clinic.
- Talk with client about their **relationships** and their **sexual** **behavior**, **condom** **use**, **condom** **negotiation**, **abstinence**. Give client **condom** **demonstration** and **condoms**.
- **Refer** participants who report psychological, sexual, or physical harm by their partners **(intimate partner violence)** to proper services.
- **Refer** participants with medical or psychological problems to *[doctor 1]* or *[doctor 2]*.
- **Encourage** client to continue until they get two “not detected” RT-PCR test results in a row.

**For Pregnant Participants only (at Post-Test Visit)**

- The participant will have already received her pregnancy results from the nurse after she took the test.
- Confirm her results with her:
  - “I have the results from the pregnancy test you did with the nurse earlier. It shows you are pregnant. How do you feel about this?” *Probe: ask how she is feeling and whether she wants to discuss anything about the pregnancy with you.*
- Give participant information about Ebola and pregnancy:
  - There is no evidence to show that women who survive Ebola and then become pregnant can pose a risk for Ebola virus transmission
  - It is very important for all pregnant women to receive antenatal care (ANC) to keep the participant and her baby safe.
- Ask participant if she is attending/receiving antenatal care:
  - If Yes:
    - *Document which ANC she already attends by writing a note-to-file*
    - *Inform receptionist*
  - If No:
    - We can help you find antenatal care in your area. You can speak to the nurses and doctors in the M34 survivor clinic next door today to be connected to antenatal care. Would you like to do that?
    - *Refer to survivor clinic for subsequent referral to appropriate ANC.*
- Inform the participant that maintaining good health is important in pregnancy.
  - Your body works extra hard when you are pregnant so it is important to take good care of yourself. Part of this is a healthy diet. I would like to provide you with some nutritious biscuits which will help keep you healthy.
    - *Distribute nutritional supplement bars and refer to guidance sheet to give instructions.*

1. These body fluids were grouped together due to similarity in the structure of the counseling sessions and content of associated behavioral guidance. [↑](#footnote-ref-1)
2. [↑](#footnote-ref-2)
3. World Health Organization. Clinical management of patients with viral haemorrhagic fever: A pocket guide for front-line health workers*. Interim emergency guidance - for country adaptation*. Updated 26-Oct-2015. [↑](#footnote-ref-3)
4. World Health Organization. Responding to intimate partner violence and sexual violence against women: WHO clinical and policy guidelines. 2013. Accessed at http://apps.who.int/iris/bitstream/10665/85240/1/9789241548595_eng.pdf?ua=1 [↑](#footnote-ref-4)
5. World Health Organization. Responding to intimate partner violence and sexual violence against women: WHO clinical and policy guidelines. 2013. Accessed at http://apps.who.int/iris/bitstream/10665/85240/1/9789241548595_eng.pdf?ua=1 [↑](#footnote-ref-5)
